# 27th Annual Computational Neuroscience Meeting (CNS*2018): Part Two

**DOI:** 10.1186/s12868-018-0451-y

**Published:** 2018-10-29

## P200 Robust regulation of neuronal dynamics by the Na/K pump

### Gennady Cymbalyuk^1^, Christian Erxleben^2^, Angela Wenning-Erxleben^2^, Ronald Calabrese^2^

#### ^1^Georgia State University, Neuroscience Institute, Atlanta, GA, United States; ^2^Emory University, Department of Biology, Atlanta, GA, United States

##### **Correspondence:** Gennady Cymbalyuk (gcymbalyuk@gmail.com)

*BMC Neuroscience* 2018, **19(suppl 2):**P200

Reliable function of neuronal networks, especially of those that control vital motor functions, is crucial for the survival and health of organisms. Here, we investigate the role of Na+/K+ pump, which is the basic cellular engine maintaining the physiological gradients of Na+ and K+ ions across the membrane. Growing evidence indicates that the Na+/K+ pump current plays important roles in the electrical activity of neurons, contributing to functional and dysfunctional dynamics [1–6]. To understand how the pump dynamics contribute to neuronal activity, the kinetics of Na+/K+ pump function has to be quantitatively evaluated versus the dynamics of neurons and the stability of their activity regimes. The premise of this study is that the Na+/K+ pump contributes to the dynamics of neurons on the time scale of the period of their rhythmic bursting activity (6–10 s). In the leech heartbeat CPG, the basic building blocks are half center oscillators (HCO), which are pairs of mutually inhibitory HN interneurons producing alternating bursting activity. The neuropeptide myomodulin speeds up the period of the bursting pattern by increasing h-current and decreasing the Na+/K+ pump current in the heart interneurons (HNs), thus implicating the pump in the bursting dynamics of this CPG [6]. A definitive role of the Na+/K+ pump current in the bursting dynamics of HN neurons was revealed by using monensin, which stimulates the pump by diffusively increasing the intracellular Na+ concentration. In the presence of h-current application of monensin decreases the period of a leech heartbeat HCO [4]. To make quantitative analysis possible, we developed a dynamic clamp implementation of a hybrid system with living neuron and mathematical model in real time in each of which we can manipulate pump parameters on the fly (Fig. 1). The implemented model had been described in the appendix of [4]. We explored parameter space of the full model and fine tune the real time model to support functional-like dynamics of HCO under variation of the key biophysical parameters. By focusing our analysis on a common network motif of mutually inhibitory units, we generated insights that generalize from our experimentally and computationally more tractable invertebrate system to less accessible vertebrate nervous systems.

**Fig. 1:**
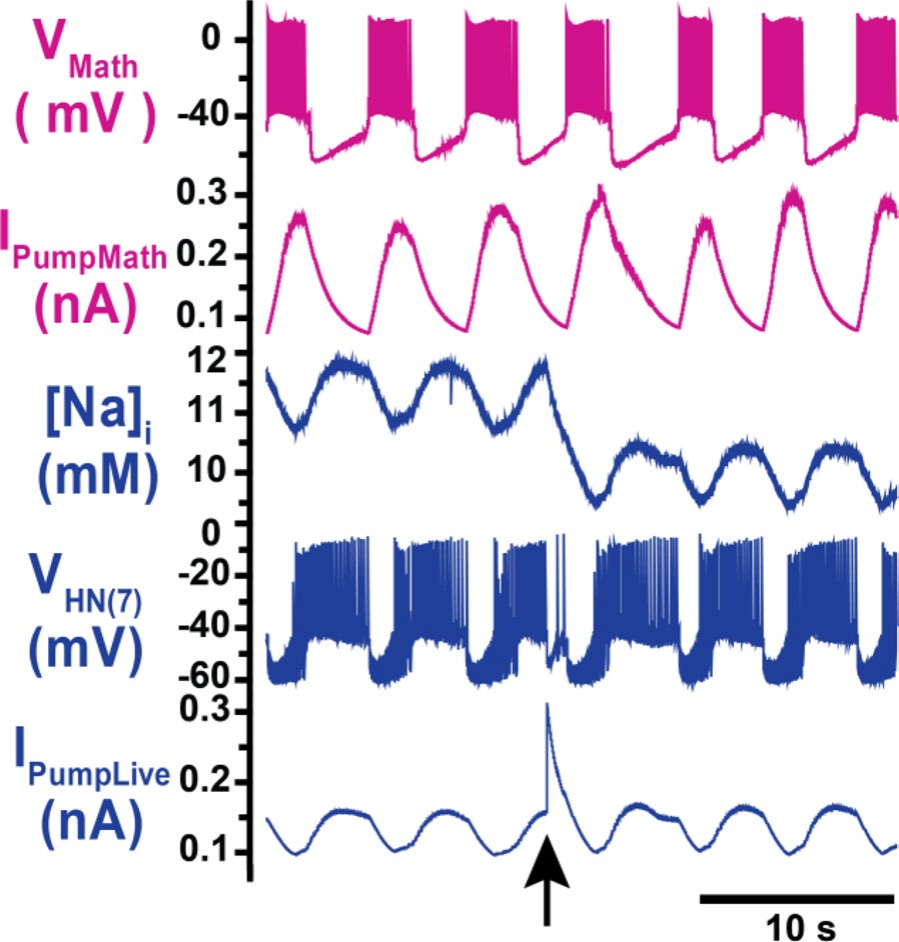
Bursting exhibited by a hybrid system comprised of a real-time HN model (pink) with Na+/K+ pump incorporated and living synaptically isolated HN (blue) from ganglion 7 with dynamic-clamp IPump scaled by parameter I_pump^max. The pump currents grow during bursts, governed by intracellular Na+ concentration [Na]i. In the living cell, the added pump current is controlled by virtual [Na]i. At the beginning of the trace I_(pump Math)^max = 1 nA, and I_(pump Live)^max = 0.5 nA and at the arrow both I_(pump)^max were increased by factor of 2. The change caused decrease of [Na]i., but did not significantly affect the period of the HCO

**Acknowledgements**

Supported by NINDS 1 R01 NS085006 to RLC.

**References**

1. Krishnan GP, et al. Electrogenic properties of the Na(+)/K(+) ATPase control transitions between normal and pathological brain states*. J Neurophysiol*, 2015. 113(9): p. 3356–74.
2. Picton LD, et al. Sodium Pumps Mediate Activity-Dependent Changes in Mammalian Motor Networks. *J Neurosci*, 2017. 37(4): p. 906–921.
3. Picton LD, Zhang H, Sillar KT. Sodium pump regulation of locomotor control circuits. *J Neurophysiol*, 2017. 118(2): p. 1070–1081.
4. Kueh D, et al. Na(+)/K(+) pump interacts with the h-current to control bursting activity in central pattern generator neurons of leeches. *Elife*, 2016. 5.
5. Zhang HY, Sillar KT, Short-term memory of motor network performance via activity-dependent potentiation of Na+/K+ pump function. *Current Biology* : CB, 2012. 22(6): p. 526–31.
6. Tobin AE, Calabrese RL. Myomodulin increases Ih and inhibits the NA/K pump to modulate bursting in leech heart interneurons. *Journal of Neurophysiology*, 2005. 94(6): p. 3938–50.

## P201 Gender differences in intrinsic oscillations of the resting brain following brief mindfulness intervention

### Yi-Yuan Tang^1^, Rongxiang Tang^2^

#### ^1^Texas Tech University, Lubbock, TX, United States; ^2^Washington University in St. Louis, Psychological and Brain Sciences, St. Louis, WA, United States

##### **Correspondence:** Yi-Yuan Tang (yiyuan.tang@ttu.edu)

*BMC Neuroscience* 2018, **19(suppl 2):**P201

Gender differences have been shown in various cognitive domains, brain functions and pathological population. However, the role of gender in responding brief mindfulness intervention remains largely unexplored. We applied fractional amplitude of low-frequency fluctuation (fALFF) to examine gender differences in intrinsic oscillations of the resting brainbefore and after mindfulness intervention. fALFF has been widely used to examine brain differences and abnormalities in healthy and patient populations, it measures the power spectrum intensity of spontaneous brain frequency oscillations and could identify the role of gender-related differences in the resting-state brain activity following intervention. We trained 38 college students (21 males) for 1 month (30 min per session for 20 sessions, 10 h in total). The mindfulness intervention is IBMT which has been used in our series of randomized controlled trails [1–3]. All resting-state fMRI scans were collected at pre- and post- intervention in a 3-Telsa Siemens Skyra. Similar to the procedures of previous literature [2, 4], the time series of each voxel was transformed to a frequency domain after the linear trend was removed without band-pass filtering. The square root was then calculated at each frequency of the power spectrum, and finally the sum of amplitude across 0.01–0.08 Hz was divided by that across the entire frequency range to obtain fALFF. The IBMT fALFF maps before and after the intervention were compared using paired t test. All results were corrected for multiple comparisons (p corrected < 0.05), based on Monte Carlo stimulation. Before intervention there were no significant differences in the brain resting fALFF and behavior (e.g., mood states) between males and females. After the intervention, behaviorally we did not detect any significant difference. However, males and females showed different resting state activities, and specifically, males have higher activity mainly in sensory motor areas, cingulate cortex and insula than females. These results are consistent with previous findings of gender differences in sleep states, cognitive performance and social functioning [5, 6], and indicate that males and females respond to mindfulness intervention differently in the brain resting states. We should take the gender differences into consideration in future intervention studies.

**References**

1. Tang YY, Holzel BK, Posner MI. The neuroscience of mindfulness meditation. *Nat Rev Neurosci* 2015, 16, 213–225
2. Tang YY, Tang R, Posner MI. Brief meditation training induces smoking reduction. *Proc Natl Acad Sci USA* 2013, 110, 13971–13975
3. Tang YY, et al. Central and autonomic nervous system interaction is altered by short term meditation. *Proc Natl Acad Sci USA* 2009, 106, 8865–70
4. Zou QH, et al. An improved approach to detection of amplitude of low-frequency fluctuation (ALFF) for resting-state fMRI: Fractional ALFF. *J Neurosci Methods* 2008, 172, 137–141
5. Dai XJ, et al. Gender differences in brain regional homogeneity of healthy subjects after normal sleep and after sleep deprivation: a resting-state fMRI study. *Sleep Med .*2012, 13, 720
6. Zhang C, Dougherty CC, Baum SA, et al. Functional connectivity predicts gender: Evidence for gender differences in resting brain connectivity. Hum Brain Mapp 2018, 10.1002/hbm.23950. [Epub ahead of print]

## P202 Tonic-to-bursting transitions in synchronous gap junction coupled neurons

### Epaminondas Rosa, Rosangela Follmann

#### Illinois State University, School of Information Technology, Normal, IL, United States

##### **Correspondence:** Epaminondas Rosa (erosa@ilstu.edu)

*BMC Neuroscience* 2018, **19(suppl 2):**P202

Synchronization in neurological systems is critical for the survival of many species. Vital functions such as locomotion and mastication, for example, depend upon mechanisms yielding robust and stable neuronal synchronization. Moreover, pathologies including Parkinson’s disease and sleep disorders, for instance, are associated with neuronal synchrony deficiencies rendering patients incapable of leading a normal life. In this study we describe a transition recently observed in computer simulations of gap junction coupled neurons. The transition is mediated by a period-doubling cascade followed by chaos in synchronous neurons, with their firing regimes evolving from tonic (fast repetitive spiking) to bursting (periods of repetitive fast spiking followed by periods of quiescence) as a coupling parameter is increased. While tonic-to-bursting transitions play important roles, for instance, in thalamocortical neurons at sleeping transition states (Sherman, Trends Neurosci, 2001), and in sensory-motor nuclei that generate the typical tremors in Parkinson’s disease (Llinas and Steriade, J Neurophysiol, 2006), little is known about the mechanisms regulating and controlling theses transitions at the level of the dynamic of the individual networked neurons. We use a Hodgkin-Huxley type model neuron (Rosa et al., Biosystems, 2015) to investigate the transition between tonic and bursting neuronal behaviors in small networks of electrically coupled neurons. Numerical simulations show that distinct neurons, one tonic and the other bursting, reciprocally coupled via gap-junctions, depending upon the individual characteristics of the two neurons, may synchronize either in the tonic or in the bursting regime, remaining in the state in which they first synchonized for extended increments in the strength of their coupling. However, we also found that in some cases, the two neurons synchronize initially in the tonic regime and with increased coupling strength, undergo a period doubling bifurcation cascade in route to chaos, go through chaos and then, still in synchrony, go into the bursting regime. Intriguing, we noticed that some peculiar common features of the independent single neurons are preserved when they are coupled and synchronized. For example, the characteristic firing rate at the border between tonic and bursting regimes for the individual neuron is passed on to the collective, when pairs of distinct neurons synchronize (Shaffer et al. PRE, 2016). Similar results were obtained for the case of triads of gap-junction coupled neurons (Shaffer et al. Eur Phys Journal ST, 2017).

## P203 Resting-state dynamics in a large-scale spiking model of the visual areas of macaque cortex

### Maximilian Schmidt^1^, Rembrandt Bakker^2^, Kelly Shen^3^, Gleb Bezgin^4^, Claus Hilgetag^5^, Markus Diesmann^6^, Sacha van Albada^7^

#### ^1^RIKEN Brain Science Institute, Wako-shi, Germany; ^2^Radboud University, Donders Institute for Brain, Cognition and Behavior, Nijmegen, Netherlands; ^3^Baycrest, Rotman Research Institute, Toronto, Canada; ^4^McGill University, McConnell Brain Imaging Centre, Montreal, Canada; ^5^University Medical Center Eppendorf, Department of Computational Neuroscience, Hamburg, Germany; ^6^Jülich Research Centre, Institute of Neuroscience and Medicine (INM-6) & Institute for Advanced Simulation (IAS-6), Juelich, Germany; ^7^Jülich Research Centre, Institute for Advanced Simulation (IAS-6), Juelich, Germany

##### **Correspondence:** Sacha van Albada (s.van.albada@fz-juelich.de)

*BMC Neuroscience* 2018, **19(suppl 2):**P203

Cortical resting-state dynamics is organized on multiple spatiotemporal scales and involves cell-type-specific spike rates, slow and fast fluctuations, clustered inter-area correlations, and inter-area activity propagation. Simulations of large parts of cortex resolving the individual neurons and synapses enable studying how cortical network structure shapes this multi-scale activity, but have been limited by the available computational resources and simulation technology. Developments in the simulation technology of NEST and access to the JUQUEEN supercomputer have enabled us to overcome this barrier, and simulate a network of 32 vision-related areas of macaque cortex with each area represented by a 1 mm2 microcircuit with the full density of neurons and synapses [1], which avoids distortions due to downscaling [2]. The simulations rely on a recently derived connectivity map for the visual areas of macaque cortex that predicts the connection probability between any two neurons based on their types, areas, and layers [3]. This connectivity map integrates axonal tracing data with predictions from cortical architecture (neuron densities, layer thicknesses), inter-area distances, and neuronal morphologies. In line with models using simplified equations for the individual areas [4], our model predicts that cortex operates in a metastable state where slow activity fluctuations appear. In this regime, the power spectrum of simulated V1 spiking activity and the distribution of spike rates across V1 neurons agree well with those from parallel spike recordings in lightly anesthetized macaque [5]. Furthermore, the inter-area functional connectivity is similar to that from macaque resting-state fMRI [6]. The simulated neuronal activity propagates across areas mainly in the feedback direction, akin to LFP findings during sleep [7]. A mean-field-based analysis [8] shows that the order of activations of the areas is strongly associated with local stability properties, such that the most unstable areas are activated first. Our model reconciles microscopic and macroscopic accounts of cortical neural networks and provides a platform for further developments.

**Acknowledgements**

The European Union Seventh Framework Programme under Grant Agreement No. 604102 (Human Brain Project, HBP) and the European Union’s Horizon 2020 research and innovation programme under Grant Agreement No. 720270 (HBP SGA1), the German Research Council (DFG Grants SFB936/A1,Z1, TRR169/A2, and SPP 2041), and Grant JINB33 for computing time on the JUQUEEN supercomputer.

**References**

1. Schmidt M, Bakker R, Shen K, et al. Full-density multi-scale account of structure and dynamics of macaque visual cortex. *arXiv* 2015, preprint arXiv:1511.09364.
2. Van Albada SJ, Helias M, Diesmann M. Scalability of asynchronous networks is limited by one-to-one mapping between effective connectivity and correlations. *PLoS computational biology* 2015, 11(9), e1004490.
3. Schmidt M, Bakker R, Hilgetag CC, et al. Multi-scale account of the network structure of macaque visual cortex. *Brain Structure and Function* 2018, 223(3), 1409–1435.
4. Cabral J, Kringelbach ML, Deco G. Exploring the network dynamics underlying brain activity during rest. *Progress in neurobiology* 2014, 114, 102–131.
5. Chu CC, Chien PF, Hung CP. Tuning dissimilarity explains short distance decline of spontaneous spike correlation in macaque V1. *Vision research* 2014, 96, 113–132.
6. Everling, S, Babapoor-Farrokhran S, Hutchison RM, Gati JS, Menon RS. Functional connectivity patterns of medial and lateral. *J Neurophysiol* 2013, 109, 2560–2570.
7. Nir Y, Staba RJ, Andrillon T, et al. Regional slow waves and spindles in human sleep. *Neuron* 2011, 70(1), 153–169.
8. Schuecker J, Schmidt M, van Albada SJ, et al. Fundamental activity constraints lead to specific interpretations of the connectome. *PLoS computational biology* 2017, 13(2), e1005179.

## P204 In the footsteps of learning: Changes in network dynamics and dimensionality with task acquisition

### Merav Stern^1^, Shawn Olsen^2^, Eric Shea-Brown^1^, Yulia Oganian^3^, Sahar Manavi^2^

#### ^1^University of Washington, Department of Applied Mathematics, Seattle, WA, United States; ^2^Allen Institute for Brain Science, Modelling, Analysis and Theory, Seattle, WA, United States; ^3^University of California, San Francisco, School of Medicine, San Francisco, CA, United States

##### **Correspondence:** Merav Stern (merav.stern@mail.huji.ac.il)

*BMC Neuroscience* 2018, **19(suppl 2):**P204

When we learn a new task, changes in our neural activity take place in order to accumulate and act upon relevant information. These changes can appear with different magnitudes in multiple brain areas. To understand the dynamics and ultimately the mechanisms of these changes, we follow mice as they learn to perform a visual change detection task and use wide-field GCaMP signaling to record their neural activity across the dorsal surface of the cortex. We also study random neural network models with cortical-resembling high-level area structures; by iteratively training these networks to perform the task we assess the similarities and differences in the mouse cortex and artificial recurrent networks. We find that initially, during the naïve behavioral stage, the visual cortex alone responds to the changing stimuli. As the learning progresses, frontal areas respond as well, and eventually, at the expert level, the whole mouse cortex responds to task-relevant stimuli. Cortical activity becomes correlated across all areas, and responses in general become more stereotyped with precise temporal dynamics. Moreover, the dimension of this activity decreases as training progresses. Our artificial neural networks show similar learning-related phenomena. All together, we identify three cortex-wide phenomena that emerge during learning of a basic sequential task: task-specific engagement of surprisingly widespread areas across cortex, an increase in the temporal precision and stereotypy of cortical activity, and a reduction of its dimensionality. These phenomena occur in both mouse cortex and in trained, minimally structured artificial neural networks, suggesting that they may recur across many learning systems and posing intriguing questions for further theoretical work.

## P205 Implementation of CA1 microcircuits model in NetPyNE and exploration of the effect of neuronal/synaptic loss on memory recall

### Ángeles Tepper^1^, Adam Sugi^2^, William W Lytton^3^, Salvador Dura-Bernal^3^

#### ^1^Pontifical Catholic University of Chile, Santiago, Chile; ^2^Universidade Federal do Paraná, Curitiba, Brazil; ^3^SUNY Downstate Medical Center, Department of Physiology and Pharmacology, Brooklyn, NY, United States

##### **Correspondence:** Ángeles Tepper (angelestepper@gmail.com)

*BMC Neuroscience* 2018, **19(suppl 2):**P205

The hippocampus has a major role in learning and memory, spatial navigation, emotional behavior and regulation of hypothalamic functions [1]. Many models of its circuitry have been developed in order to further understand its functions [2]. CA1 microcircuitry has been proposed to be responsible for the heteroassociative declarative memories [3] and the cycles of storage and recall are supposed to be modulated by theta oscillations [4] Cutsuridis et al. [5] modeled the CA1 microcircuitry using NEURON, the leading simulator in the neural multiscale modeling domain. The purpose was to investigate the biophysical mechanisms by which processes of storage and recall of spatio-temporal input patterns are achieved, employing a detailed biophysical representation of the CA1 microcircuitry. The model included five cell types whose functional roles were evaluated in the simulations. Each neuron had a specific morphology, ionic and synaptic properties, connectivity, and spatial distribution that closely followed experimental evidence. The original model was implemented in NEURON using HOC. The deprecated HOC language and the lack of standardization in NEURON makes it hard to understand, reproduce and manipulate and to run parallel simulations. Such a complex data-driven biologically realistic network would benefit from a separation of model parameters and implementation. To address these issues, we re-implemented the model using NetPyNE (www.netpyne.org), a high-level Python interface to the NEURON simulator, which facilitates the development, parallel simulation and analysis of biological neuronal networks [6]. NetPyNE employs a standardized declarative format to describe the model specifications, and can then generate an efficiently parallelized NEURON model. It also provides a large number of analysis functions that enable further exploration of the model and allows exportation to NeuroML, a standard format for computational models. Our NetPyNE implementation is able to reproduce the results of the original model, but using a clean and powerful declarative language, which makes this complex model accessible to a wider community of neuroscientists. Furthermore, we analyse and explore the model in new ways, including connectivity analysis, computation of LFP spectra and information flow. We also perform novel manipulations to elucidate the relation between neuronal and synaptic loss, involved in Alzheimer’s disease, and memory recall performance.

**References**

1. Anand, KS, Dhikav, V Hippocampus in health and disease: An overview. *Annals of Indian Academy of Neurology.*2012; 15(4), 239
2. Bezaire MJ, Raikov I, Burk K, Vyas D, Soltesz I Interneuronal mechanisms of hippocampal theta oscillations in a full-scale model of the rodent CA1 circuit. *Elife*. 2016;5. 10.7554/elife.18566
3. Treves A, Rolls ET Computational analysis of the role of the hippocampus in memory. *Hippocampus.*1994;4: 374–391
4. Hasselmo ME, Bodelón C, Wyble BP A proposed function for hippocampal theta rhythm: separate phases of encoding and retrieval enhance reversal of prior learning. *Neural Comput.*2002;14: 793–817
5. Cutsuridis V, Cobb S, Graham BP Encoding and retrieval in a model of the hippocampal CA1 microcircuit. *Hippocampus.*2010;20: 423–446
6. Lytton WW, Seidenstein A, Dura-Bernal S, Schurmann F, McDougal RA, Hines ML Simulation neurotechnologies for advancing brain research: Parallelizing large networks in NEURON. *Neural Comput.*2016

## P206 Modular science: Towards online multi application coordination on inhomogeneous high performance computing and neuromorphic hardware systems

### Abigail Morrison, Alexander Peyser, Wouter Klijn, Sandra Diaz-Pier

#### Jülich Research Centre, Institute for Advanced Simulation (IAS-6), Juelich, Germany

##### **Correspondence:** Alexander Peyser (a.peyser@fz-juelich.de)

*BMC Neuroscience* 2018, **19(suppl 2):**P206

Supercomputers are important tools for simulation and data processing. High-performance computing (HPC) workflows in neuroscience are applied in several areas: image processing, simulation, visualization, data storage, and parameter space exploration. Execution of workflows as coupled pipelines (Fig. 1) is desirable due to excessive intermediate data, the need for expert human live-interaction and interaction with multiscale components or live experiments. Launching applications on clusters requires batch scheduling, software setup and environment customization. To provide the scientific community with tools for reliable and efficient execution of complex, interactive workflows on HPC systems, we propose “Modular Science” (MS). MS is a software framework and social interaction contract for the deployment of such workflows. At its core lies the orchestration of scientific applications. The modular science orchestrator decomposes the execution of a workflow into process stages, enhancing robustness, debuggability and the probability of success with minimal effort or loss of resources for increasing number of components. MS will enable monitoring of basic workflow variables including data bandwidth, memory consumption and error tracking. Use case 1 includes interactive generation of neural network models using connectivity based on experimental data and executed with the NEST simulator [1]. Case 2 treats a simulation executed in NEST interacting with an Arbor simulation [2] and output processing with Elephant [3] and local field potential calculation [4]. Case 3 includes generation of a full brain simulation using TVB neural mass models (NMM) based on DTI data [5]. For these cases, simulation results must be compared against functional data to iteratively refine the model, interactively guided by an expert. In case 4, we plan to enable a multiscale full brain simulation combining systemic NMMs coupled to specific high-resolution regions in NEST. Firing rate output from the NMMs is coupled to spiking input for the neuron scale simulations, and output is then processed using Elephant for analysis. MS is in early stages of development. Here we present preliminary results from selected cases as a proof-of-concept for complex workflows deployed on the JSC’s infrastructure. Our framework is open source and deployable on both local clusters and supercomputing centers and is compatible with most batch systems. This framework will support the reproducibility of large workflows and robust but efficient usage of HPC and data storage resources. Such a framework will be crucial to attack new, large scale neuroscientific problems requiring complex/multiscale workflows combining pluggable simulators and analytical tools.

**Fig. 1:**
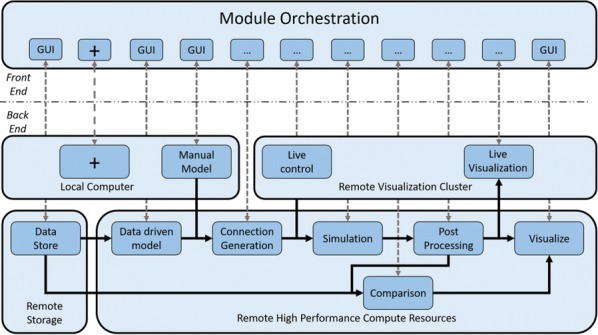
Modular Science: structure of complex workflows combining in situ visualization, scalable storage and complex pipelines including connectivity generation, pluggable simulators, sophisticated analysis and iterative interactive research. MS is a proposal to combine a software framework, social contracts and a formal software engineering language to enable the implementation of such software packages to HPC environments in a robust and debuggable manner

**References**

1. Alexander Peyser et al. NEST 2.14.0, 2017.https://juser.fz-juelich.de/record/838729
2. Wouter Klijn et al. Arbor: neural network simulator for HPC, 2016.https://eth-cscs.github.io/nestmc/
3. Alper Yegenoglu et al. Elephant – Open-Source Tool for the Analysis of Electrophysiological Data Sets. 2015.http://juser.fz-juelich.de/record/255984
4. H Lindén et al. LFPy: A tool for biophysical simulation of extracellular potentials generated by detailed model neurons, 2014.https://www.frontiersin.org/articles/10.3389/fninf.2013.00041/full
5. Sanz Leon et al. The virtual brain: a simulator of primate brain network dynamics. 2013.https://www.frontiersin.org/article/10.3389/fninf.2013.00010

## P207 Characteristic region-specific neuronal plasticity by PrP peptide aggregates in rat organotypic hippocampal slice cultures

### Sang Seong Kim

#### Hanyang University, Department of Pharmacy, Ansan, Republic of Korea

##### **Correspondence:** Sang Seong Kim (talpiot@hanyang.ac.kr)

*BMC Neuroscience* 2018, **19(suppl 2):**P207

Scrapie prion protein (PrPSc), the abnormal conformational isoform of cellular prion protein (PrPC), is tightly associated with prion pathogenesis. The neuronal cell death in the brain is the major pathophysiological consequence of PrPSc aggregates. Growing evidence indicates that brain circuit is important to maintain physiological functions of brain and impairment of a certain circuit across the brain can be translated into malfunction of physiology and behavior, which defines the clinical phenotypes of clinical states. To investigate the impact of PrPSc aggregates on the brain circuit, the amyloidogenic peptide PrP(106–126) derived from PrPC in either aggregated or non-aggregated state was challenged to organ-cultured brain section of wild type mice. The changes occurred in the brain section were monitored at the level of electrophysiology. For the functional connectivity analysis, mutual information were evaluated for each pair of 8 × 8 recording electrodes. For two CSD X = (x_1, x_2, …, x_N) and Y = (y_1, y_2, …, y_N) at each of the two channels under analysis, mutual information(MI) measures the statistical dependence between X and Y [1]. MI is similar in spirit to cross correlation, but is much more general, because MI is capable of capturing the nonlinear dependencies that the cross correlation might have missed. To estimate MI, we use the k nearest-neighbor approach [2]. In our study, Normalized Mutual information (NMI) NMI(X, Y) = (I(X, Y))/(√(I(X, X)) √(I(X, Y))) is measured to scale the results between 0 and 1.

The result indicates that aggregated, but not non-aggregated, PrP(106–126) affected CA2 and CA3 regions of hippocampal circuit with extraordinary activation of CA3 (Fig. 1). The region specific activation implies specific channel expression compared to other areas so pharmacologic study was applied.

**Fig 1:**
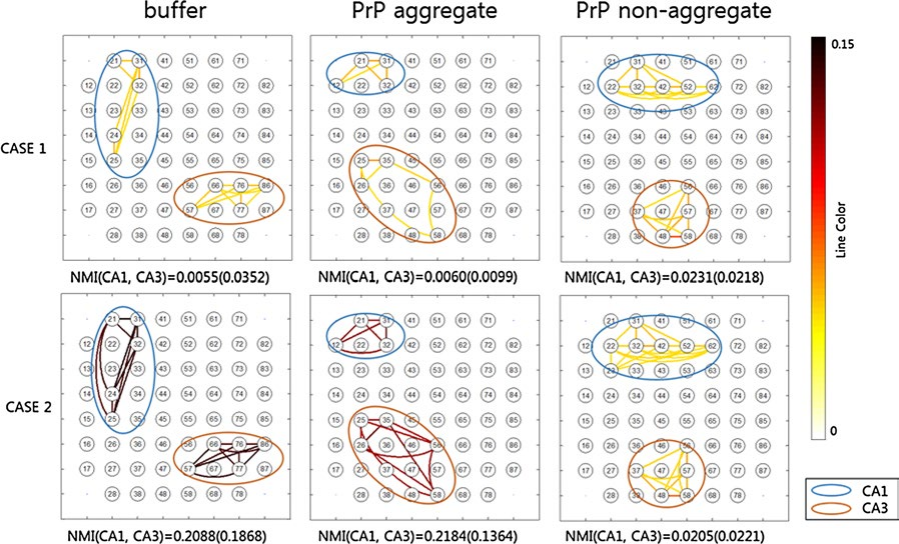
Case1 is under the circumstance when CA3 supposedly iniates the activation in the slice. Case2 presumes both CA1 and CA3 are the origin of the activity

**References**

1. Thomas M, Thomas JA. *Elements of Information Theory*. UK: John Wiley & Sons, 2012
2. Kraskov, A, Stögbauer H, Grassberger P. Estimating mutual information. *Physical review E* 2004, 69(6), 066138.

## P208 Burst control and noise modulation by Nav1.6 persistent and resurgent sodium channel currents in sensory neurons

### Sharmila Venugopal^1^, Soju Seki^2^, David H Terman^3^, Antonios Pantazis^4^, Riccardo Olcese^5^, Martina Wiedau-Pazos^6^, Scott H Chandler^2^

#### ^1^University of Arizona, Phoenix, AZ, United States; ^2^University of California, Los Angeles, Integrative Biology and Physiology, Los Angeles, CA, United States; ^3^The Ohio State University, Department of Mathematics, Columbus, OH, United States; ^4^University of California, Los Angeles, Anesthesiology Department, Los Angeles, CA, United States; ^5^University of California, Los Angeles, Departments of Anesthesiology and Physiology, Los Angeles, CA, United States; ^6^University of California, Los Angeles, Department of Neurology, Los Angeles, CA, United States

##### **Correspondence:** Sharmila Venugopal (svenugopal10@gmail.com)

*BMC Neuroscience* 2018, **19(suppl 2):**P208

**Introduction:** Voltage-gated sodium currents (VGSCs) are critical mediators of subthreshold oscillations and high frequency burst discharge in sensory neurons. The Nav1.6 type sodium channels mediate a complex form of sodium current that has three distinct components, namely, a transient (INaT), a resurgent (INaR) and, a persistent (INaP) current. Here we developed a unique three-component conductance-based model for this VGSC, and studied its role in the control of burst discharge in proprioceptive sensory neurons in the trigeminal Mesencephalic (Mes V) nucleus. Using model simulations, real-time closed-loop dynamic clamp electrophysiology and theoretical analyses of bursting dynamics, we identified a unique push–pull modulation of burst timing by the INaP and INaR components. While INaP enhanced membrane resonance, burst frequency and duration, INaR prolonged the inter-burst intervals and decreased noise sensitivity. We further utilized the model Nav1.6 conductances to restore normal burst patterns using the dynamic-clamp approach, in the abnormally bursting Mes V neurons of a neurodegenerative mouse model. Our results highlight a key role of this VGSC in information processing in these sensory neurons.

**Methods:** The Mes V neuronal model follows a conductance-based Hodgkin-Huxley formalism and incorporates a potassium leak current (Ileak), a slow 4-AP sensitive potassium current (IK), and the complex Nav1.6 VGSC with a fast/transient sodium current (INaT), a slowly inactivating persistent sodium current (INaP), and an unconventional resurgent sodium current (INaR). Model simulations were performed using MATLAB; bifurcation analysis was performed using XPPAUT. Real-time dynamic-clamp electrophysiology was performed in brainstem Mes V neurons in P8-P12 mice.

**Results:** Our results using model simulations and dynamic-clamp approach demonstrate distinct effects of INaR versus INaPcomponents in modulating burst intervals in these neurons. While INaPdecreased inter-burst intervals (IBIs), INaRprolonged the IBIs, and, increased the burst refractoriness. The latter was found to be important for correcting burst irregularities simulated by random noise, as well as, in the SOD1G93A mouse model for Amyotrophic Lateral Sclerosis. Theoretical analyses of the steady-state model behavior revealed a push–pull negative feedback control of sodium resurgence by slow channel inactivation, as an underlying biophysical mechanism of burst duration and interval control. Finally, in a simplified monosynaptic reflex network model, we demonstrated that the burst interval control offered by these sodium currents in the Mes V sensory neurons, can modulate postsynaptic trigeminal motor neuron discharge patterns.

**Conclusions:** Our results highlight a novel role for INaRin burst-timing control and noise modulation, with implications to other sensorimotor systems wherein, the Nav1.6 VGSCs underlie ectopic firing/bursting such as in neuropathic pain and spastic/epileptiform discharges.

## P209 A dynamic resource model for sequential working memory

### Hyeonsu Lee^1^, Woochul Choi^1,2^, Youngjin Park^1^, Se-Bum Paik^1,2^

#### ^1^Korea Advanced Institute of Science and Technology, Department of Bio and Brain Engineering, Daejeon, Republic of Korea; ^2^Korea Advanced Institute of Science and Technology, Program of Brain and Cognitive Engineering, Daejeon, Republic of Korea

##### **Correspondence:** Hyeonsu Lee (hslee9305@kaist.ac.kr)

*BMC Neuroscience* 2018, **19(suppl 2):**P209

Processing of sequential information is crucial to encoding various inputs from the real world, thus working memory used to store sequential information in humans is of great interest. A number of studies have reported that subjects better memorize the first and last items in a sequence than the others, and refer to this as the primacy and recency effects [1–2]. However, the underlying mechanisms of these effects are still elusive. Here, we propose a novel model of these features of sequential memory performance, by introducing the concepts of sequential overwrite and non-uniform allocation of memory resources. First, for a precise investigation of sequential memory characteristics, we performed experiments where subjects were to memorize a series of visual patterns. As previously reported, we confirmed that the correct ratio of the first and last stimuli in the sequence was higher than those for the others. To explain this result, we modified the standard resource model with the assumption that memory resources of previous information are partially replaced by newly introduced information (overwrite effect), and that memory performance for each item is proportional to the amount of resources allocated. From this sequential overwrite model with a non-uniform resource allocation from data fit, we could readily explain the observed U-shaped memory performance in human psychophysical experiments. Next, based on our model that sequential overwrite is a key factor of memory performance, we predicted that memory performance can be affected by the modulation of memory overwrite. To test our idea, we designed an experiment where subjects perform sequential memory tasks under three different conditions: correct information, no information, and wrong information about the item numbers are presented before the task. We expected that these three conditions would vary the degree of memory overwrite of the sequential items and that this would affect the performance. As predicted, correct information improved memory performance while wrong information worsened it, and this effect was most significant in earlier sequences where overwrite was stronger. Model parameters fitted to the observed results suggested that the degree of overwrite was significantly different across conditions and well explained the performance. Our model suggests that sequential overwrite and non-uniform allocation of memory resources can explain the origin of the featured U-shape of sequential memory performance. Furthermore, our model suggests a possible mechanism of optimal memory allocation with prior information of the items to memorize.

**References**

1. Hurlstone MJ, Graham JH, Baddeley AD. Memory for serial order across domains: An overview of the literature and directions for future research. *Psychological Bulletin* 2014, 339
2. Gorgoraptis N, Catalao RFG, Bays PM et al. Dynamic updating of working memory resources for visual objects. *Journal of Neuroscience* 2011, 31,

## P210 Retinal development of cortical functional circuits

### Jaeson Jang^1^, Min Song^1,2^, Se-Bum Paik^1,2^

#### ^1^Korea Advanced Institute of Science and Technology, Department of Bio and Brain Engineering, Daejeon, Republic of Korea; ^2^Korea Advanced Institute of Science and Technology, Program of Brain and Cognitive Engineering, Daejeon, Republic of Korea

##### **Correspondence:** Jaeson Jang (jaesonjang@kaist.ac.kr)

*BMC Neuroscience* 2018, **19(suppl 2):**P210

In higher mammals, the primary visual cortex (V1) is organized into various maps of visual functions such as ocular dominance, preferred orientation, and spatial frequency. It has recently been reported that the topography of these functional maps are geometrically correlated [4], such that the contours of the orientation and the spatial frequency maps intersect orthogonally [5]. This may imply an efficient tiling of processing units, but it is still unclear how this systematic organization can develop in the cortex. Here, we introduce our developmental model to suggest that the topography of the functional maps can be seeded altogether from the regularly structured retinal mosaic and that this shared origin results in topographical correlation among the maps. A previous model provides insight into our model, showing that a quasi-periodic orientation map can be seeded by themoiré interference between hexagonal lattices of ON and OFF retinal ganglion cells (RGCs) [6]. The key assumption was that the orientation tuning of a V1 neuron can be predicted bythe localalignmentof ON and OFF RGCs. This is supported by experimental observations that the structure of cortical functional maps is strongly correlated with the local organization of ON and OFF afferents [1]. Expanding this monocular model to binocular condition,we suggest that the local organization of ON and OFF RGCs in the retinal mosaic can also constrain the ocular dominance and spatial frequency preference. We found that the distance between ON and OFF RGCs could determine the separation of ON and OFF receptive field subregions of the connected V1 neuron, and could also change wiring strength to contralateral and ipsilateral feedforward circuits. With the notion that the ipsilateral connections are later developed to match the orientation preference through two pathways, our model showed that the phase difference between contra- and ipsilateral receptive fields of binocular V1 neurons could induce a preferencefor higher spatial frequency than that in the monocular region [4]. As a result, we successfully reconstructed the orthogonal relationships between orientation, ocular dominance, and spatial frequency maps, as observed in the experimental data [2, 6]. Our results suggest a unified developmental model of various functional maps in visual cortex.

**References**

1. Kremkow J, Jin J, Wang Y, Alonso JM. Principles underlying sensory map topography in primary visual cortex. *Nature* 2016, 533 (7601).
2. Hübener M, Shoham D, Grinvald A, Bonhoeffer TJ. Spatial relationships among three columnar systems in cat area 17. *Journal of Neuroscience* 1997, 17, 9270–9284
3. Issa NP, Trepel C, Stryker MPJ. Spatial frequency maps in cat visual cortex. *Journal of Neuroscience* 2000, 20, 8504–8514
4. Nauhaus I, Nielsen KJ, Callaway EM. Efficient Receptive Field Tiling in Primate V1. *Neuron* 2016, 91, 893–904
5. Nauhaus I, Nielsen KJ, Disney A, Callaway EM. Orthogonal micro-organization of orientation and spatial frequency in primate primary visual cortex. *Nat. Neurosci.* 2012, 15, 1683–1690
6. Paik SB, Ringach DL. Retinal origin of orientation maps in visual cortex. *Nat. Neurosci.* 2011, 14, 919–925

## P211 A hierarchical neural network model for non-Hebbian dynamics of memory ensemble

### Youngjin Park^1^, Se-Bum Paik^1,2^

#### ^1^Korea Advanced Institute of Science and Technology, Department of Bio and Brain Engineering, Daejeon, Republic of Korea; ^2^Korea Advanced Institute of Science and Technology, Program of Brain and Cognitive Engineering, Daejeon, Republic of Korea

##### **Correspondence:** Youngjin Park (yodamaster@kaist.ac.kr)

*BMC Neuroscience* 2018, **19(suppl 2):**P211

The basolateral amygdala (BLA) is known to be a core brain region for emotional function, such as fear memory. Recently, it was reported that observation of a memory ensemble in the BLA revealed unusual neural activities, different from the predictions of the standard Hebbian model (Blair 2001) of synaptic plasticity. Grewe et al. examined neural ensembles for conditioned stimulus (CS) and unconditioned stimulus (US) in the BLA during fear conditioning, and found that the dynamics of the individual neurons observed was contradictory to the global tendency. According to the data, the CS ensemble came to resemble the US ensemble more during the learning, but the activity of individual neurons that simultaneously receive CS and US input tended to decrease. Moreover, only a small portion of the cells with potentiated CS responses were responsive to the US; thus these responses alone cannot explain the global changes in the CS and US ensembles. From this, Grewe et al. concluded that there must be hidden elements, such as a hypothetical neuromodulator, that produce the observed result. Here, we suggest an alternative solution: a hierarchical model with segregated learning and coding layers with standard Hebbian plasticity. Our key idea is that neural populations for information coding and associative learning may be separate. In the previous model for analysis of observed data, it was assumed that learning and coding occur simultaneously in the same neural layer, so bi-directional change in the neural ensembles could not be explained. However, if the output coding layer receives projections from a separate former layer, the observed non-Hebbian behavior of output ensemble might not be paradoxical. To test our idea, we constructed a two-layer feedforward network model for computer simulation. We assumed that the CS and US ensemble patterns were first formed in the input layer, and that their activity patterns were then projected to neurons in the output layer to form the observed CS and US ensemble in the output layer. During the conditioning, we implemented a stochastic change of neural activity in the input CS ensemble following the Hebbian model that neurons that overlap the US ensemble increase their response, and neurons that do not overlap the US ensemble decrease response, both with a constant probability. Under this condition, we could reproduce the experimental observation that the CS/US overlap ratio in the output layer increased. In addition, we also found bi-directional changes in activity within the output layer, similar to those observed in the BLA data. Our result suggests that the observed non-Hebbian ensemble dynamics could originate from the projection of pure Hebbian dynamics, raising an issue about the fundamental organization of memory consolidation circuits.

## P212 Data-driven models of interneurons in the somatosensory thalamus and comparison with gene expression data

### Elisabetta Iavarone^1^, Jane Yi^1^, Ying Shi^1^, Christian O’Reilly^1^, Werner Alfons Hilda Van Geit^1^, Christian A Rössert^1^, Henry Markram^1^, Sean Hill^2^

#### ^1^École Polytechnique Fédérale de Lausanne, Blue Brain Project, Lausanne, Switzerland; ^2^University of Toronto & EPFL, Centre for Addiction and Mental Health and Blue Brain Project, Toronto, Canada

##### **Correspondence:** Elisabetta Iavarone (elisabetta.iavarone@epfl.ch)

*BMC Neuroscience* 2018, **19(suppl 2):**P212

The thalamic reticular nucleus is the major source of inhibition to the thalamus. However, different thalamic nuclei in the rodent brain receive varying degree of inhibition from local interneurons, ranging from 15 to 20% of the neuronal population in the visual thalamus to < 4% in the somatosensory thalamus [1]. Despite the lower abundance of thalamic interneurons compared to excitatory thalamo-cortical (TC) cells, they have been shown to shape visual responses and to dynamically influence the extent of receptive fields [2]. As the morphological and electrophysiological properties of TC cells in first-order thalamic nuclei show high degree of similarity across modalities (e.g., visual and somatosensory systems) [3], we hypothesized that local interneurons in different sensory circuits have similar cellular and synaptic properties, and explored them with the aid of data-driven computational models, in vitro patch-clamp recordings, and gene expression data. We characterized the properties of mice TC neurons of the ventrobasal (VB) nucleus and local interneurons by applying a standardized battery of electrical stimuli, biocytin staining and 3D morphological reconstruction. We qualitatively classified the passive responses and firing properties into different electrical types (e-types) and validated the classification by extracting electrical features from the voltage traces. We then used the 3D morphologies, electrical features, ionic current kinetics and distribution from experimental findings to constrain multi-compartmental models of the different e-types by using a multi-objective optimization strategy [4] and validated them with stimuli not used during model building. We complemented our data analysis and modelling pipeline by comparing the modelled e-types with single cell and synaptic properties systematically curated from the neuroscientific literature [5], along with gene expression data. The result of this analysis suggests that while some thalamic e-types are comparable to interneurons in cortical microcircuits, others are thalamus-specific and comparable to interneurons from the dorsal part of the lateral geniculate nucleus [6].

**References**

1. Arcelli P, Frassoni C, Regondi MC, et al. GABAergic neurons in mammalian thalamus: a marker of thalamic complexity? *Brain Research Bulletin* 1997, 42, 27–37.
2. Heiberg T, Hagen E, Halnes G, Einevoll GT. Different Effects of Triadic and Axonal Inhibition on Visual Responses of Relay Cells. *PLoS Computational Biology* 2016, 12(5), e1004929.
3. Sherman SM, Guillery RW. (2006) *Exploring the Thalamus and its Role in Cortical Function*. Cambridge, MA: MIT Press.
4. Van Geit W, Gevaert M, Chindemi G, et al. H: BluePyOpt: Leveraging open source software and cloud infrastructure to optimise model parameters in neuroscience. *Front. In Neuroinform* 2016, 10
5. O’Reilly C, Iavarone E, and Hill SL. A Framework for Collaborative Curation of Neuroscientific Literature. *Front. In Neuroinform* 2017, 11, 27.
6. Leist M, Datunashvilli M, Kanyshkova T, et al. Two types of interneurons in the mouse lateral geniculate nucleus are characterized by different h-current density. *Scientific Reports* 2016, 6, 24904.

## P213 Network connectivity effects on multisensory integration in neocortex

### Svetlana Gladycheva, Bailey Conrad, Sean Powell

#### Towson University, Department of Physics, Towson, MD, United States

##### **Correspondence:** Svetlana Gladycheva (sgladycheva@towson.edu)

*BMC Neuroscience* 2018, **19(suppl 2):**P213

We investigate the role of synaptic connectivity in the cortical network that may lead to a better understanding of autism spectrum disorder (ASD). We have established a measure and studied properties of the integration of distinct stimuli in the cortical network model and investigated effects of network connectivity on this integration. Taken with ongoing experimental optogenetic studies [1], this model may assist to pave the way toward potential pharmacological targets for the treatment of ASD.

Autism is a neurodevelopmental disorder for which there is no cure. It is characterized by impairments in social cognition and communication. Abnormalities in the ASD brain are not strictly localized, but involve multiple neural networks [2, 3]. Numerous studies suggest a deficit in multisensory integration (MSI) in autism, both in human and animal models [4, 5]. Specifically, it has been shown that ASD patients demonstrate a widened window of audio-visual temporal integration [6]. It has been proposed that deficits in the integration of multisensory cues leading to ASD are likely to be caused by dysfunctional connectivity in the brain [6, 7, 8].

Multisensory integration by neural populations in the cortex has been recently studied in many contexts [9, 10, 11]. Our model is an adaptation of the Traub model [12] of a single-column thalamocortical network, modified to be used in the GENESIS neuronal simulation environment [13]. The model comprises 14 types of cortical neurons each with its own compartmental morphology and electrophysiological properties connected in columnar structure. We apply pulse train stimuli to the cells at two different locations in the column and measure the local field potential (LFP) to characterize network activity.

Our model demonstrates that the multiple distinct stimuli generate superadditive integrated LFP response, where the combined stimuli from two locations produces a larger response than the sum of the two individual ones. We then use this model to investigate the effect of network connectional parameters on temporal aspects of multisensory integration in the cortex. It is believed that the ASD condition may be associated with widened temporal windows of cortical integration. Existing ASD therapies concentrate on behavioral interventions that reduce symptoms and, to date, no drug therapy exists for ASD that would repair or strengthen brain circuits. With our model’s measure of MSI and cholinergic impairment, it may be possible to gauge the efficacy of pharmacological agents whose action would ameliorate the ASD condition.

**References**

1. Yi, Feng et al. Hippocampal ‘cholinergic Interneurons’ Visualized with the Choline Acetyltransferase Promoter: Anatomical Distribution, Intrinsic Membrane Properties, Neurochemical Characteristics, and Capacity for Cholinergic Modulation, *Frontiers in Synaptic Neuroscience* 2015, 7, 4.
2. Muller, RA The study of autism as a distributed disorder, *Ment Retard Disabil Res Rev.* 2007, 13, 85–95.
3. Rippon G et al. Disordered connectivity in the autistic brain: challenges for the “new psychophysiology”. *International Journal of Psychophysiology* 2007, 63, 164–172.
4. Robertson CE and Baron-Cohen S, Sensory perception in Autism. *Nature Reviews Neuroscience* 2017, 18, 671–684.
5. Belmonte MK et al. Autism and abnormal development of brain connectivity. *Journal of Neuroscience* 2004, 24, 9223–9231.
6. Anagiustou E et al. Review of neuroimaging in autism spectrum disorders: what we have learned and where we go from here. *Molecular Autism* 2011, 2, 4.
7. Wass S. Distortions and disconnections: disrupted brain connectivity in autism. *Brain and Cognition* 2011, 75, 18–28.
8. Stevenson RA et al. Identifying and quantifying Multisensory Integration: a Tutorial. *Review Brain Topography* 27, 2014, 707–730.
9. Fetsch CR et al. Bridging the gap between theories of sensory cue integration and the physiology of multisensory neurons. *Nature Reviews Neuroscience* 2013, June 14(6).
10. Ursino M et al. Neurocomputational approaches to modeling multisensory integration in the brain: a review. *Neural Networks* 2014, 60, 141–165.
11. Traub RD et al. Single column thalamocortical network model exhibiting gamma oscillations, sleep spindles and epileptic bursts. *Journal of Neurophysiology* 2005 Apr, 93(4): 2194–232.
12. Boothe DL et al. Impact of neuronal membrane damage ona local field potential in a large scale simulation of the neuronal cortex. *Frontiers in Neurology* 2017, 8, 236.

## P214 A general method to generate artificial spike train populations

### Samira Abbasi^1^, Dieter Jaeger^2^, Selva Maran^2^

#### ^1^Hamedan University of Technology, Biomedical Engineering, Hamedan, Islamic Republic of Iran; ^2^Emory University, Department of Biology, Atlanta, GA, United States

##### **Correspondence:** Dieter Jaeger (djaeger@emory.edu)

*BMC Neuroscience* 2018, **19(suppl 2):**P214

Synaptic decoding of neural population activity at the single cell level presents a challenging question. One method to address this question in a rigorous way is to use detailed single neuron simulations with well-defined input patterns to study the input–output function of biophysically realistic neurons. In previous work we developed method to create artificial spike trains (ASTs) that can match spike train properties of cerebellar Purkinje cells in order to study the cerebellar cortical-nuclear signal transformation [1]. Here we generalize this method to create well defined artificial spike trains (ASTs) made from templates of different types of recorded neurons and further test the method with surrogate data. The basic idea of our method is to use recorded neurons to construct rate templates of their activity using gaussians. Then we can draw gamma distributed spike trains from these rate templates to obtain ASTs with different regularity properties. We can scale templates to different firing rates, add a refractory period to the gamma distributions, and add well defined rate-correlations between multiple ASTs by manipulating the rate template. Here we first tested our method with constant rate templates, sinusoidal rate templates, and zap rate templates. We find that slow rate fluctuations (~ 1 Hz) can be well captured by individual ASTs, but that faster rate fluctuations require a population average of ASTs to recapture the rate template. The ability to capture faster rate fluctuations is a function of the regularity (kappa parameter of the gamma distribution) and the rate of the ASTs that are being generated. These properties parameterize fundamental limits of coding rate fluctuations with noisy spike trains. We then use pyramidal neuron and mossy fiber recordings from the cerebellar to test our algorithms for real data beyond the fast firing Purkinje cell populations previously used. Unlike cerebellar Purkinje cells which exhibit high firing rate and more regular spike trains, most pyramidal neurons and mossy fibers exhibit low firing rates and highly irregular and bursty firing pattern. In spite of these differences in firing rate and pattern, ASTs created using our method were able to match the statistical properties of spike trains in both these cell types. The ability to re-create original rate templates from such ASTs was limited by the same features as seen for our surrogate rate templates, and reveal limitations as to how faithfully low rate/high variability spike trains can communicate a rate code.

**Reference**

1. Abbasi S, et al. Robust transmission of rate coding in the inhibitory Purkinje cell to cerebellar nuclei pathway in awake mice. *PLoS Computational Biology* 2017, 13, e1005578.

## P215 Laminar contributions to auditory feature processing

### Vergil Haynes^1^, Sharon Crook^2^

#### ^1^Arizona State University, College of Mathematical and Statistical Sciences, Tempe, AZ, United States; ^2^Arizona State University, School of Life Sciences, Tempe, AZ, United States

##### **Correspondence:** Vergil Haynes (vrhaynes@asu.edu)

*BMC Neuroscience* 2018, **19(suppl 2):**P215

Understanding the contributions of different layers to cortical processing within the visual and somatosensory systems has led to testable hypotheses about how multiple simple sensory features are combined within cortical columns of these areas. This understanding provides a concrete basis for bridging high-level models of information processing with proposed neurobiological components and recordings of neuronal activity. To complement these studies, insight into cortical oscillations provides a convenient framework for understanding population processing of sensory features. In particular, auditory cortex demonstrates an organizational hierarchy of rhythmic activity and these rhythms effect stimulus encoding [1]. Previous studies further implicate phase-resetting and reciprocal interlaminar interactions in feature selection [2, 3]. Large-scale computational modeling studies of sensory systems have focused primarily on visual and somatosensory cortices. Despite recent advancements in characterizing anatomical and physiological properties of the auditory system, few models reconstruct fundamental differences between the auditory system and other modalities. Here we present a model that incorporates some features unique to auditory cortex and replicates various statistical response properties of a non-primary auditory area [4]. The model is a modification of a biologically realistic model of a thalamocortical network with multiple layers [5] converted for broader usage [6]. Simulations were performed within the NEURON simulation environment [7] using NetPyNe [8] for model handling and analysis.

The model provides predictions about how convergent inputs carrying distinct information are processed within a thalamocortical network and demonstrate relationships between laminar cortical oscillations and interlaminar processing of convergent inputs. We also investigate how phase-resetting reorganizes laminar processing and affects feedforward and feedback outputs. Our model simulations assume auditory processing relies on a hierarchical network structure. Feedforward inputs from earlier auditory populations (in area A1) are provided to the network based on statistical response patterns of multi-unit activity to a repertoire of auditory stimulation found in the same literature used to replicate non-primary auditory responses [4]. The model is modified and tuned to replicate the response properties of later areas under similar stimulation protocols. As the model uses a biophysically based multicompartmental formalism, we demonstrate how convergent extrinsic inputs shape local population activity reflected in both population firing and local field potentials.

**References**

1. Lakatos P, Shah AS, Knuth KH, et al. An Oscillatory Hierarchy Controlling Neuronal Excitability and Stimulus Processing in the Auditory Cortex. *Journal of Neurophysiology* 2005, 94(3)
2. Guo W, Clause AR, Barth-Maron A, Polley DB. A Corticothalamic Circuit for Dynamic Switching between Feature Detection and Discrimination. *Neuron* 2017, 95(1), 180–194.
3. Carracedo LM, Kjeldsen H, Cunnington L, et al. A Neocortical Delta Rhythm Facilitates Reciprocal Interlaminar Interactions via Nested Theta Rhythms. *Journal of Neuroscience* 2013, 33(26), 10750–10761
4. Traub RD, Contreras D, Cunningham MO, et al. Single-column thalamocortical network model exhibiting gamma oscillations, sleep spindles, and epileptogenic bursts. *Journal of Neurophysiology* 2005, 93(4), 2194
5. Kajikawa Y, de le Mothe LA, Blumell S, et al. Coding of FM sweep trains and twitter calls in area CM of marmoset auditory cortex. *Hearing Research* 2008, 239, 107–125.
6. Gleeson P, Steuber V, Silver RA. neuroConstruct: a tool for modeling networks of neurons in 3D space. *Neuron* 2007, 54(2), 219–235.
7. Carnevale NT, Hines ML. *The NEURON Book.* MA: Cambridge University Press, 2006.
8. Lytton WW, Seidenstein AH, Dura-Burnell S, et al. Simulation Neurotechnologies for Advancing Brain Research: Parallelizing Large Networks in NEURON. *Neural Computation* 2016, 28(10), 2063–2090.

## P216 Rapid selection of NeuroML models via NeuroML-DB.org

### Justas Birgiolas^1^, Richard Gerkin^1^, Sharon Crook^2^

#### ^1^Arizona State University, School of Life Sciences, Tempe, AZ, United States; ^2^Arizona State University, School of Mathematical and Statistical Sciences, Tempe, AZ, United States

##### **Correspondence:** Justas Birgiolas (justas@asu.edu)

*BMC Neuroscience* 2018, **19(suppl 2):**P216

Biophysically realistic computational models are ideally suited for simulation of predictions that can be verified experimentally via chemical, surgical, and optogenetic manipulations. NeuroML is a modular, declarative, simulator-independent model language for describing and exchanging such models. NeuroML-DB.org is an online resource for rapidly locating NeuroML models by keyword search or ontological (anatomical or neurotransmitter) relations [1]. Rapidly locating models with desired dynamical and computational properties is an ongoing challenge. While NEURON simulator models can be examined via ModelDB, NEURON-based channel model dynamical properties can be viewed via ICGenealogy project [2], and NeuroML based models can be shared on the OpenSourceBrain.org website, a resource that allows viewing of systematic evaluations of NeuroML models has not been available. We have extended our previous work on NeuroML-DB and enabled interactive, visual inspection of temporal dynamics of channel models. Channel activation, deactivation, and inactivation voltage clamp protocols similar to those used in the ICGenealogy project were utilized to record the current and conductance responses of 133 NeuroML channel models available in NeuroML-DB. The resulting web application, accessible viaNeuroML-DB.org, allows visual inspection of, including zooming and panning, the temporal dynamics of channel currents and conductances during channel activation, inactivation, and deactivation voltage clamp protocols (Fig. 1, model from [3]). For channels with [Ca2+] dependent dynamics, the effect of [Ca2+] on the channel output response can be interactively examined in the interface. Additionally, files that reproduce the plots of each channel response can be downloaded via the interface. Using the jNeuroML library, the files can be converted to several other simulator formats. Finally, channel detail web pages display notes, if any, regarding numerical or simulation issues encountered while running the simulations.

**Fig. 1:**
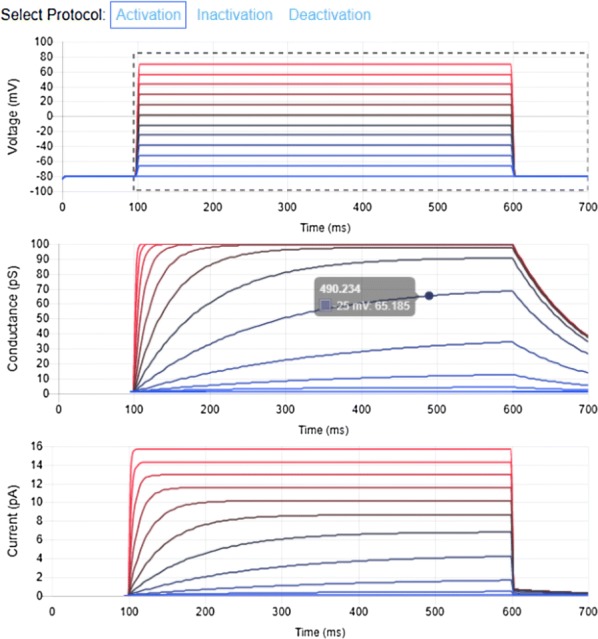
Screen capture of interactive plots of a sodium channel model’s [3] voltage clamp protocol response. Dashed line represents the region of interest that is shown in the middle and bottom plots. Top: voltage clamp waveform used to stimulate the channel. Middle and bottom: conductance and current responses of the channel. Hovering with mouse pointer reveals the values of each curve, while mouse scrolling and mouse dragging actions allow zooming and panning into different regions of the plots

A channel model implemented in NeuroML can be converted to a wide range of formats and included in larger cell and network models, regardless of the original simulator used to implement the channel model. This work allows modelers to rapidly locate and visually inspect the dynamical properties of NeuroML channels and assess their suitability for inclusion in larger models. In ongoing work, we are utilizing the cell stimulation protocols [4] from the Allen Brain Atlas project to characterize the responses of NeuroML cell models. The cell protocols include ramp, step, threshold, and pink noise current injections and assess properties such as resting voltage, rheobase and threshold currents for all NeuroML cell models in NeuroML-DB, as well as quantify model run time complexity.

**Acknowledgments**

JB was supported by NIH grant F31DC016811 to JB and JB, SC, and RG were supported by NIH R01MH1006674 to SC.

**References**

1. Birgiolas J, et al. *Proc 27th Int Conf Sci Stat Db Mgmt* 2015, 37.
2. Podlaski WF, Seeholzer A, Groschner LN, et. al. ICGenealogy: Mapping the function of neuronal ion channels in model and experiment.*bioRxiv*2016. 10.1101/058685.
3. McCormick DA, Wang Z, Huguenard J. Neurotransmitter control of neocortical neuronal activity and excitability. *Cereb Cortex* 1993, 3(5), 387–98.
4. Allen Cell Types Database (Oct. 2017 v.5) Electrophysiology.http://help.brain-map.org/display/celltypes/Documentation.

## P217 Multiscale model validation with SciUnit

### Richard Gerkin^1^, Russell J. Jarvis^1^, Sharon Crook^2^

#### ^1^Arizona State University, School of Life Sciences, Tempe, AZ, United States; ^2^Arizona State University, School of Mathematical and Statistical Sciences, Tempe, AZ, United States

##### **Correspondence:** Richard Gerkin (rgerkin@asu.edu)

*BMC Neuroscience* 2018, **19(suppl 2):**P217

Computational models of biological systems are rarely *formally* tested for agreement between model output and experimental data. *SciUnit*, a software framework for model validation, facilitates such rigorous testing. During model development, models can be continuously subjected to data-driven “unit tests” that quantitatively summarize model-data agreement, identifying modeling progress and highlighting output that fails to adequately reproduce observed data from the corresponding biological system. The OpenWorm Project is an international open-source collaboration to create a multiscale model of the organism *C. elegans*. At every scale, including subcellular, cellular, network, and behavior, this project employs one or more computational models that aim to recapitulate the corresponding biological system at that scale. This requires that the simulated behavior of each model be compared to experimental data both as the model is continuously refined and as new experimental data become available. We present data-driven OpenWorm model validation using*SciUnit*at three model scales: 1) ion channels, 2) neurons, and 3) whole organism motor output. This workflow is publicly visible and accepts community contributions to ensure that modeling goals are transparent and well-informed. Model validation tests are executed continuously as the models are updated and refined, ensuring that development converges towards the ultimate design specification: agreement with the underlying biological system.

## P218 Rivalry with irregular spiking: a comparison of mutual inhibition and random networks

### Benjamin Cohen, Carson Chow, Shashaank Vattikuti

#### National Institute of Health, NIDDK, Lab of Biological Modeling, Bethesda, MD, United States

##### **Correspondence:** Benjamin Cohen (benjapaulcohen@gmail.com)

*BMC Neuroscience* 2018, **19(suppl 2):**P218

Perceptual rivalry is the subjective experience of alternations between competing percepts when an individual is presented with an ambiguous stimulus. Mutual inhibition between pools of neurons encoding different interpretations of the stimulus is thought to underlie this disambiguation computation, where activity in one pool dominates and the corresponding percept is represented. A canonical cortical circuit model with mutual inhibition and fatigue can explain normalization, winner-take-all, rivalry, and various findings from psychophysics experiments [1]. However, this approach has yet to incorporate realistic spiking statistics.

Meanwhile, balanced state theory has been used to explain why cortical neurons fire irregularly [2]. Researchers have modeled computing orientation selectivity and working memory in balanced networks, but competitive networks have only recently been investigated [3]. A recent study showed that alternations resembling rivalry result from random networks receiving stochastic but competitive inputs [4]. Here we explore a model of perceptual rivalry with realistic spiking. First, we show that normalization, winner-take-all, and rivalry behaviors can coexist with a realistic asynchronous-irregular state. Next, we compare the psychophysical properties of this model to those of a random network with stochastic input. Our model can explain Levelt’s second and fourth propositions, a gamma distribution of dominance times, and can maintain a coefficient of variation of dominance times which is stable across changes in the input. By contrast, a random network cannot explain Levelt’s fourth proposition, and does not reproduce a gamma distribution of dominance times.

**References**

1. Vattikuti S, Thangaraj P, Xie HW, et al. Canonical Cortical Circuit Model Explains Rivalry, Intermittent Rivalry, and Rivalry Memory. *PLOS Computational Biology* 2016, 12(5), e1004903
2. van Vreeswijk C, Sompolinsky H. Chaotic Balanced State in a Model of Cortical Circuits. *Neural Computation* 1998, 10: 1321–1371
3. Shaham N, Burak Y, Slow diffusive dynamics in a chaotic balanced neural network. *PLOS Computational Biology* 2017, 13(5): e1005505
4. Rosenbaum R, Smith MA, Kohn A, et al. The Spatial Structure of Correlated Neuronal Variability, *Nature Neuroscience* 2017, 20(1), 107–114

## P219 An ensemble modeling approach to identifying cellular mechanisms in thoracic sympathetic neurons

### Kun Tian^1^, Astrid Prinz^1^, Michael McKinnon^2^, Shawn Hochman^2^

#### ^1^Emory University, Department of Biology, Atlanta, GA, United States; ^2^Emory University, Department of Physiology, Atlanta, GA, United States

##### **Correspondence:** Kun Tian (ktmelody@gmail.com)

*BMC Neuroscience* 2018, **19(suppl 2):**P219

Thoracic sympathetic postganglionic neurons (tSPNs), innervated by preganglionic neurons in the spinal cord, are the last common motor output of the sympathetic nervous system, and directly control the vasculature and other internal organs. Dysfunction of tSPNs, such as hyperexcitability, has been observed after spinal cord injury, yet little is known about the cellular mechanisms that drive the excitability of tSPNs.

Combining electrophysiological data with computational modeling, we built the first physiologically-realistic single neuron model of tSPN in mice, and elucidated several cellular mechanisms that govern the tSPN dynamics. For example, we found that the post-inhibitory rebound that has been observed in tSPNs ex vivo was induced by the sodium and potassium currents (INaand IKd) instead of the T-type calcium current. We also found that both the M-type potassium current (IM) and the calcium-dependent potassium current (IKCa) were necessary to replicate the spike rate adaptation. Together, we reproduced all the essential features of tSPNs ex vivo with eight types of ionic currents, which are INa, IKd, IM, IKCa, a fast transient potassium current (IA), a persistent calcium current (ICaL), a hyperpolarization-activated inward current (Ih), and a leak current (IL). Using this single neuron model, we employed an ensemble modeling approach to build a database of physiologically-realistic tSPN models [1–3], which enables a more comprehensive and rigorous examination of the range of tSPN responses to various synaptic inputs. Overall, this work lays the foundation to examine both the recruitment principles of synaptic inputs at tSPNs and the dysfunction of tSPNs after spinal cord injury in the future.

**Acknowledgements**

Ensemble modeling was performed on the Neuroscience Gateway Portal [4]. This work is supported by the CMBC Interdisciplinary Neuroscience Pilot Research Fund at Emory University.

**References**

1. Prinz AA. Computational approaches to neuronal network analysis. *Biological Sciences* 2010, 365:2397–2405.
2. O’Leary T, Sutton AC, Marder E. Computational models in the age of large datasets. *Current Opinion in Neurobiology* 2015, 32:87–94.
3. Gao P, Ganguli S. On simplicity and complexity in the brave new world of large-scale neuroscience. *Current Opinion in Neurobiology* 2010, 32:148–155.
4. S Sivagnanam, A Majumdar, K Yoshimoto, V Astakhov, A Bandrowski, M. E. Martone, and N. T. Carnevale. Introducing the Neuroscience Gateway, IWSG, volume 993 of CEUR Workshop Proceedings, CEUR-WS.org, 2013

## P220 Functional connectivity in mouse visual cortex revealed by large scale recordings

### Xiaoxuan Jia, Joshua Siegle, Gregg Heller, Séverine Durand, Shawn Olsen

#### Allen Institute for Brain Science, Modelling, Analysis and Theory, Seattle, WA, United States

##### **Correspondence:** Xiaoxuan Jia (jxiaoxuan@gmail.com)

*BMC Neuroscience* 2018, **19(suppl 2):**P220

The mammalian visual cortex is composed of multiple areas that are organized in a hierarchical structure, with feedforward, feedback and horizontal connections. The bottom-up convergent connections generate larger spatial receptive fields and longer temporal integration windows at higher levels of the visual hierarchy. While the structure of these anatomical connections is relatively fixed on short timescales, the structure of functional interactions can rapidly change conformation due to changes in external stimuli and internal brain states. This flexibility is critical for selectively routing signals for perception, cognition, and behavior. Therefore, understanding how neurons form functional networks is fundamental for deciphering brain functions. Studies that investigate functional networks with resting-state fMRI can image the entire brain at once, but, due to the low temporal resolution of this method, they fail to uncover network dynamics at fast timescales that are important for many aspects of perception and decision-making. Studies that attempt to measure functional connectivity with electrophysiological recordings have typically been restricted to recordings from two brain areas at a time, with a limited number of simultaneously neurons in each dataset. Therefore, novel methods are needed for recording large populations of neurons with sufficient temporal resolution to study dynamic functional connectivity at a large scale. Here we make use of the newly developed Neuropixels probe, which contains 384 densely arranged recording sites along a linear shank. We built a platform to simultaneously record from 6 independent probes inserted in the mouse visual cortical areas including primary visual cortex (V1) and 5 higher-order visual cortical areas (LM, RL, AL, PM, and AM). The linear probes are inserted across the layers of the cortex in head-fixed awake mice. The high yields of the Neuropixels probe allow us to record simultaneously from more than 700 well-isolated neurons distributed across cortical layers and areas in the visual cortex of a single mouse. To maximize the probability of finding mono-synaptic functional connections, we mapped the retinotopy of each area with intrinsic signal imaging and specifically target regions with overlapping visual fields. This targeting is validated by receptive field mapping. To compare functional networks under the context of different sensory inputs, we studied activity during drifting gratings and natural movies, in addition to comparing with spontaneous non-stimulus driven activity. We used two methods to measure functional connectivity within the visual cortical network: fine timescale pairwise cross-correlogram (CCG) analysis and Granger causality analysis, both of which can reveal functional relationships of recorded neurons. We found that both the proportion of effective connections and the strength of the functional connection decay as a function of receptive field separation. The time delay of effective connections revealed layer-specific functional sub-networks, based on cortical layers estimated from current source density analysis. We also observed significant differences in functional connectivity between gratings, movies, and spontaneous activity. In sum, our platform provides a unique opportunity to directly study millisecond-timescale functional networks across 6 highly interconnected cortical areas.

## P221 Building individualized dynamic brain models at high spatial resolution using fMRI

### Matthew Singh^1^, Todd Braver^2^, ShiNung Ching^3^

#### ^1^Washington University, St. Louis, Department of Neuroscience, St. Louis, MO, United States; ^2^Washington University, St. Louis, Department of Psychology, St. Louis, MO, United States; ^3^Washington University, St. Louis, Electrical and Systems Engineering, St. Louis, MO, United States

##### **Correspondence:** Matthew Singh (msingh4@vols.utk.edu)

*BMC Neuroscience* 2018, **19(suppl 2):**P221

In the current era of big data and ever improving methods for data acquisition, several initiatives have produced innovative whole-brain dynamical systems models of neural activity. Unfortunately, there remains a gap in (large-scale) systems neuroscience communities between generative models of brain dynamics vs. descriptive or statistical models of brain activity. The latter has proven highly amenable to data-driven characterizations of individual differences, while the former allows for the overt construction of mechanistic hypotheses regarding circuit function. In the present work we attempt to bridge this gap and present a procedure to fit independent large-scale dynamical-systems models to individual subjects using fMRI. Through a novel fitting procedure, we parameterize networks of hundreds to thousands of nonlinear neural masses (brain regions) each with their own intrinsic dynamics and connections for individual human subjects using resting state fMRI. We rigorously assess psychometric properties and sensitivity to preprocessing choices and a variety of nuissance factors. We perform multiple validity and reliability tests. Results demonstrate that our procedure is robust, reliable, and (for the cases tested) valid. We demonstrate that these models provide novel insight into both large-scale and local dynamics including a “hierarchy of intrinsic time scales” and spatial organization in a previously unexplored feature: transfer function curvature. By forward simulating individual subject’s models we recreate individual subject’s “dynamic functional connectivity” (dFC) patterns as well as higher-order phenomena such as dwell-time and transition probability between dFC patterns despite using time-invariant models (Fig. 1).

**Fig. 1:**
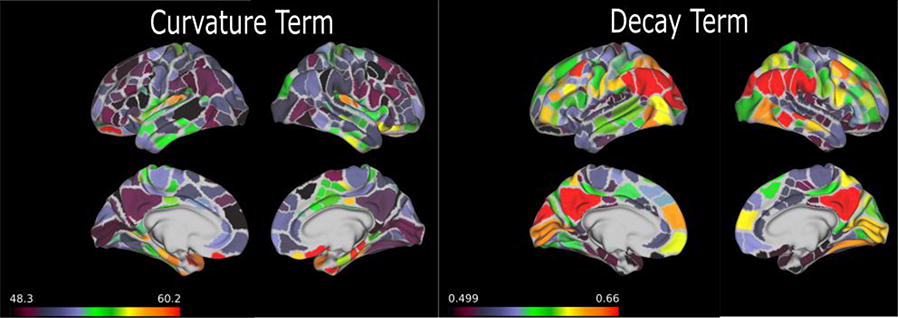
Transfer function curvature (left) and decay (right) parameters reveal a smooth spatial distribution. The modeling procedure thus allows the study of both distributed dynamics (the full model) and local dynamics (decay parameters) at high spatial resolution

## P222 Revisiting efficient coding of natural sounds in the environment: unsupervised learning or task-based optimization?

### Hiroki Terashima, Shigeto Furukawa

#### NTT Communication Science Laboratories, Sagamihara, Japan

##### **Correspondence:** Hiroki Terashima (teratti@teratti.jp)

*BMC Neuroscience* 2018, **19(suppl 2):**P222

Efficient coding has been a leading computational principle for the sensory neuroscience. Following the visual system, Lewicki [1] argued that the auditory periphery can be explained by unsupervised learning of natural sounds. One of the study’s claims is that the basis optimized to code human voice resembles the auditory nerve fibres, whose filter sharpness distribution is preserved across mammals. We were able to reproduce the matched distribution by applying the same algorithm to clean recordings of human voices. However, we also found that an efficient code for human voices recorded in the natural environment shows much sharper tuning than the auditory nerve fibres, even though the environment recording is closer to the sensory signal our ears receive and more natural than a studio recording. Our analysis showed that the waveforms are distorted on a short time scale comparable to the time window of the auditory nerve filters and that the mismatch can be reproduced by simulating environmental reverberations, suggesting that the primary factor in the mismatch is the environmental reverberations. How can we better model the auditory periphery including the environmental modulations? Inspired by a recent work on the visual hierarchy [2], we hypothesized that the auditory periphery is optimized to perform auditory tasks we face in the natural environment instead of unsupervised learning of the entire incoming signal. To test this, we built a deep convolutional neural network that receives reverberated waveform inputs. As a naturalistic task related to voices on a short time scale comparable to the time window of the auditory periphery, we chose phoneme classification. Waveforms and phone labels were taken from the TIMIT database. Each input had the length of 2000 data points, with the target phoneme at the centre. The input waveforms were convolved with an impulse response randomly chosen from a database [3]. After the training, the waveform filters learned in the first layer showed characteristics similar to the auditory nerve fibres, whereas a normal efficient code for the same input did not. This result does not depend on the speech dataset, since we could reproduce a qualitatively similar result by applying a different task, that is, classification of environmental sound recordings. Overall, the results suggest that the auditory periphery efficiently encodes task-related information in a reverberation-resistant manner rather than the entire incoming signal and that our understanding of sensory systems in the natural environment, not only the visual system, can be furthered by using a framework of task-based optimization.

**References**

1. Lewicki MS. Efficient coding of natural sounds. *Nature Neuroscience* 2002, 5, 356–363.
2. Yamins DLK, Hong H, Cadieu CF. Performance-optimized hierarchical models predict neural responses in higher visual cortex. *PNAS* 2014, 111(23), 8619–8624.
3. Traer J, McDermott JH. Statistics of natural reverberation enable perceptual separation of sound and space. *PNAS* 2016, 113(48).

## P223 Emergence of auditory-system-like representation of amplitude modulation in a deep neural network trained for sound classification

### Takuya Koumura, Hiroki Terashima, Shigeto Furukawa

#### NTT Communication Science Laboratories, Atsugi, Japan

##### **Correspondence:** Takuya Koumura (koumura@cycentum.com)

*BMC Neuroscience* 2018, **19(suppl 2):**P223

Amplitude modulation (AM) of a sound contains essential information for auditory perception, and neural representation of AM has been targeted by a number of neurophysiological studies. These studies generally indicate that neurons in the auditory nervous system (ANS) exhibit tuning to AM frequency, usually characterized by the synchronization to the stimulus AM and the average spike rate during the stimulus presentation. The patterns of the tuning systematically change with the processing stages along the path from the periphery to the cortex (Joris et al., 2004, Physiol Rev). However, functional significance of such representation is not well understood. Machine learning is an effective technique for investigating functional significance in sensory nervous systems (Olshausen & Field, 1996, Nature). A model is designed to be optimal for reproducing a function of the sensory nervous system without assuming its anatomical or physiological properties. Thus characteristics emerging in the optimized model should reflect only the nature of the task and the input data. Especially, a deep neural network (DNN) is suitable for this purpose because it can take raw data as inputs and process them for behaviourally relevant tasks (Yamins, et al., 2014, PNAS). The present study attempts to understand the functional significance of the AM representation in the ANS by modelling the ANS with a DNN trained for sound classification. To directly compare the AM representation in the DNN with that in the ANS, we applied neurophysiological methods for analysing the DNN, and characterized the AM tuning of the units in each layer of the DNN. Similarly to the ANS, most units in the trained DNN exhibited low-pass or band-pass AM tuning. The activities of the units in the lower layers synchronized to high AM frequency, whereas those in the higher layers synchronized to low AM frequency. The average activities exhibited AM tuning only in the higher layers. We calculated the pairwise similarities of the layers in the DNN and the regions in the ANS, and found that the tunings in the lower/higher layers in the DNN were similar to the tunings in the peripheral/central regions in the ANS (Fig. 1). Such characteristics were not observed in the DNN before optimization, but gradually emerged during the optimization. By conducting the same analysis in the DNNs with different architectures, we showed that similarity to the ANS significantly correlated with the classification accuracy of the DNN. These results suggest that the AM representation in the ANS also emerged during optimization to natural sound recognition. This work was supported by JSPS KAKENHI Grant Number JP15H05915.

**Fig. 1:**
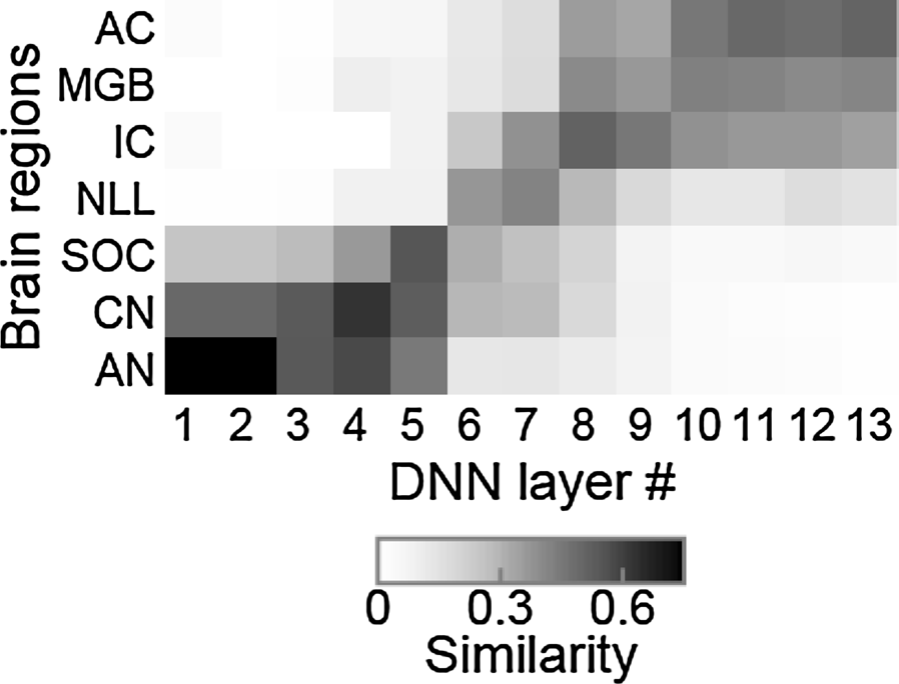
Similarity of the layers in the DNN (abscissa) and the regions in the ANS (ordinate). AN: auditory nerves, CN: cochlear nucleus, SOC: the superior olivary complex, NLL: the nuclei of the lateral lemniscus, IC: the inferior colliculus, MGB: the medial geniculate body, AC: the auditory cortex

**Acknowledgments**

Grant-in-Aid for Scientific Research on Innovative Areas “Innovative SHITSUKSAN Science and Technology.

## P224 Reproducing the cognitive function with the robustness against the brain structure and with the efficient learning algorithm

### Yoshihisa Fujita, Shin Ishii

#### Kyoto University, Graduate School of Informatics, Kyoto, Japan

##### **Correspondence:** Yoshihisa Fujita (fujita-y@sys.i.kyoto-u.ac.jp)

*BMC Neuroscience* 2018, **19(suppl 2):**P224

Computational modeling of biological neural networks which generate cognitive functions has difficulties compared to artificial neural networks. One major problem is the individual variability of the brain structure which includes extensively different connectivity patterns of neurons for the same function. Another problem is that the widely-used learning algorithms in artificial neural networks such as error backpropagation have high computational costs and are not biologically plausible. Therefore, the models of biological neural networks require both robustness against the network structure and an efficient, plausible learning algorithm. How to achieve highly cognitive functions under these constraints is largely unknown. To tackle this issue, we developed a neural network model based on Extreme Learning Machine (ELM), which includes random and fixed connections. We assumed that this randomness corresponds to the structural variability. ELM utilizes its random connections and thus its learning algorithm is quite simple. Using ELM, we tried to implement the function to recognize words from a string of letters. Since this function requires the process to integrate letters into words, we adopt Vector Symbolic Architecture (VSA), in which the patterns of neural activities for recognized objects are expressed as binary vectors and the integrating process is expressed as a vector operation. We developed a new learning model combining ELM and VSA. Unlike ordinary ELM, the balance between excitatory and inhibitory neurons was crucial in our model, which is consistent with biological findings. Within this balanced condition, it could successfully learn the vocabulary with low computational costs. We used this model to examine how neural representations of misspellings are different from those of correct spellings. Our model can provide a clue how the brain achieves cognitive functions efficiently under the structural variability.

## P225 A pipeline for macro-scale connectomics of the common marmoset with global fiber reconstruction from diffusion MRI

### Ken Nakae^1^, Junichi Hata^2^, Henrik Skibbe^1^, Alexander Woodward^3^, Carlos Gutierrez^4^, Hiromichi Tsukada^4^, Gong Rui^3^, Ryo Ito^1^, Hideyuki Okano^2^, Shin Ishii^1^

#### ^1^Kyoto University, Graduate School of Informatics, Kyoto, Japan; ^2^RIKEN BSI, Laboratory for Marmoset Neural Architecture, Wako, Japan; ^3^RIKEN BSI, Neuroinformatics Japan Center, Wako, Japan; ^4^OIST, Neural Computation Unit, Okinawa, Japan

##### **Correspondence:** Ken Nakae (nakae-k@sys.i.kyoto-u.ac.jp)

*BMC Neuroscience* 2018, **19(suppl 2):**P225

Common marmosets (Callithrix jacchus) are non-human primates with a small brain size (~ 8 g), mature quickly, and can be genetically manipulated. These features make them suitable for understanding the complex structure and connectome of the primate brain, and provide another animal for inter-species comparison. The complexities of the primate are also shown through individual structure and connectome differences. The RIKEN BSI team obtain diffusion magnetic resonance imaging (dMRI) data of a large number of marmoset brains (~ 50) to construct macro-scale connectomes and reveal the individuality between brains. Using dMRI, we can observe water diffusion phenomena within small voxels (200 μm isotropic) across the brain and use this to estimate the direction of fiber bundles through voxels. Observing the whole brain with dMRI and connecting these estimated fiber directions between adjoining voxels allows us to estimate long-range fiber bundles through the white matter of the brain. Here, we propose a pipeline for obtaining the macro-scale connectome of RIKEN’s marmoset data and analyze their individual differences of the structure and connectome. The pipeline mainly consists of two components: (1) global reconstruction of fibers, and (2) improved parcellation of brain regions by a deep learning technique. (1) Because the fiber structure of the marmoset brain is complex, we often observe multiple and different directions of fiber bundles through a voxel of dMRI. We focus on the Bayesian method for global fiber reconstruction using dMRI (Reisert, et. al. 2011), which can successfully distinguish these different directions in Human dMRI analysis. (2) To make the connectivity matrix between brain regions, we parcellated individual brain regions and counted the number of fiber bundles between ROIs. We developed a structural registration method with the help of a recent deep learning technique for parcellating the white and gray matter (VoxResNet). We estimated the cortex, white matter, subcortical regions and cerebellum in the individual brains using this method. This rough parcellation of the brain can enhance the precise correspondence between the individual and a standard brain with atlas, in which a detailed anatomical structure was defined. We analyzed RIKEN’s data with our pipeline and obtained the individual and average connectivity matrix of the marmoset brain. The graph structure of the connectivity matrices of marmosets show an exponential decay rule (EDR), which states that the strength of connectivity between two regions decays exponentially with respect to the distance between them. This rule is consistent with the graph structure of the connectivity matrices found in studies of other species (mouse and macaque monkey). We evaluated and visualized the individuality of marmoset connectivity matrices using t-SNE; a low-dimensional manifold mapping method. We found that we could discriminate between all individuals through their connectivity matrices, despite variations in the experimental settings of the dMRI and b-values.

## P226 Top-down influence on V1 responses caused by reinforcement learning of adaptive behavior

### Yoshiki Kashimori^1^, Ryo Tani^1^, Shiro Yano^2^

#### ^1^University of Electro-Communications, Dept. of Engineering Science, Chofu, Tokyo, Japan; ^2^Tokyo University of Agriculture and Technology, Division of Advanced Information Technology and Computer Science, Tokyo, Japan

##### **Correspondence:** Yoshiki Kashimori (kashi@pc.uec.ac.jp)

*BMC Neuroscience* 2018, **19(suppl 2):**P226

We can recognize rapidly and effortlessly complex visual scenes. Such amazing ability in visual recognition needs the effective processing of visual information along the multiple stages of visual pathways. Neurophysiological experiments have provided evidence for a “simple-to-complex” processing model based on a hierarchy of increasing complex image features, performed along the feedforward pathway of the ventral visual system. On the other hand, visual system has abundant feedback connections, whose number is even larger than the feedforward ones. Li et al. [1] showed that top-down signals allowed neurons of the primary visual cortex (V1) to engage stimulus components that are relevant to a perceptional task and to discard influences from components that are irrelevant to the task. They showed that V1 neurons exhibited tuning curves modulated depending on the task context. We demonstrated what kinds of top-down signals generate the tuning curves [2]. However, it remains unclear how top-down signals reflecting task behaviors emerge and how they modulate the tuning curves of V1 neurons. To address this issue, we develop a model of visual system that consists of networks of V1 a higher visual area, and a recognition area. We consider one of the perceptual tasks used by Li et al., or bisection task. Neurons of the higher visual area receive the top-down signal reflecting a decision of the task, as well as the feedforward inputs from V1 neuron, and feed the outputs back to V1 neurons. We also use a reinforcement learning to acquire an adaptive behavior to the task. The synaptic weights from neurons of the higher visual area to those in the recognition area were determined by Mirror Decent (MD) method [3] on the basis of error rate of behavior. The synaptic weights involved in top-down signals in three areas were shaped by Hebbian learning, concurrently with the learning of the feedforward connections. We show here how the feedforward and feedback connections involved in the three areas are formed by the learning of adaptive behavior. Top-down signals are generated concurrently with the acquisition of adaptive behavior. We also show that the tuning modulations of V1 neurons are caused by the change in activity through long-range connections of V1 neurons, elicited by top-down signals from the recognition area to V1 via the higher area. Our model provides the results on the tuning properties of V1 neurons that are compatible with the experimental results by Li et al. These results provide insights into understanding how behavior affects information processing in early sensory areas.

**References**

1. Li W, Piech V, and Gilbert CD, Perceptual learning and top-down influences in primary visual cortex. *Nat Neurosci* 2004, 13(3): 900–913.
2. Kamiyama A, Fujita K, Kashimori Y, A neural mechanism of dynamic gating of task-relevant information by top-down influence in primary visual cortex. *BioSystems* 2016, 150:138–148.
3. Miyashita M, Yano S, and Kondo T, Mirror decent and acceleration. 2017, arXiv:1709.02535.

## P227 Uncertainpy: A Python toolbox for uncertainty quantification and sensitivity analysis of computational neuroscience models

### Geir Halnes^1^, Gaute Einevoll^1^, Simen Tennøe^2^

#### ^1^Norwegian University of Life Sciences, Faculty of Science and Technology, Aas, Norway; ^2^University of Oslo, Department of Informatics, Oslo, Norway

##### **Correspondence:** Geir Halnes (geih@nmbu.no)

*BMC Neuroscience* 2018, **19(suppl 2):**P227

Computational models in neuroscience typically contain a number of parameters that are uncertain, either because they vary between cells or dynamically within a cell, or because they are difficult to measure accurately. Uncertainty quantification is a means to quantify the uncertainty in the model output that arise from uncertainty in the model parameters, while sensitivity analysis is the process of quantifying how much of the output uncertainty each parameter is responsible for. Unfortunately, uncertainty quantification and sensitivity analysis are not standard practices in the field of neuroscience, and models are commonly presented without any form of uncertainty quantification. To help alleviate this problem we have created Uncertainpy (https://github.com/simetenn/uncertainpy), an open-source Python toolbox, tailored to perform uncertainty quantification and sensitivity analysis of neuroscience models. Uncertainpy aims to make it easy for users to perform uncertainty quantification and sensitivity analysis without requiring detailed prior knowledge. The toolbox allows uncertainty quantification and sensitivity analysis to be performed on already existing models, and does not require changes to be made to the model implementation. Uncertainpy primarily bases its analysis on polynomial chaos expansions [1], which are faster than the more standard Monte-Carlo based approaches. Polynomial Chaos expansions are obtained from the previously developed package Chaospy [2]. Uncertainpy does not merely perform an uncertainty analysis of the “raw” model output (e.g. membrane voltage traces), but is tailored for neuroscience applications by an built-in capability of identifying characteristic features in the model output. Uncertainpy then performs an uncertainty analysis of these features. For example, the toolbox can quantify the uncertainty and sensitivity of salient model response features such as spike timing, action potential width, mean interspike interval, and other features relevant for various neural and neural network models. Uncertainpy comes with several common neuroscience models and features built in, and including custom models and new features is easy. We here present Uncertainpy, and demonstrate its broad applicability by performing an uncertainty quantification and sensitivity analysis of three case studies relevant for neuroscience: the original Hodgkin-Huxley point-neuron model [3], a multi-compartmental model of a thalamic interneuron [4] implemented in the NEURON simulator, and a sparsely connected recurrent network model [5] implemented in the NEST simulator. A preprint of this work is available at bioRxiv [6].

**References**

1. Xiu, D, Hesthaven JS. High-Order Collocation Methods for Differential Equations with Random Inputs. *SIAM Journal on Scientific Computing* 2005, *27*, 1118–1139.
2. Feinberg J, Langtangen HP. Chaospy: An open source tool for designing methods of uncertainty quantification. *Journal of Computational Science* 2015, 11, 46–57.
3. Hodgkin AL, Huxley AF. A quantitative description of membrane current and its application to conduction and excitation in nerve. *J Physiol* 1952, 117, 500–544.
4. Halnes G, Augustinaite S, Heggelund P, Einevoll GT, Migliore M. A multi-compartment model for interneurons in the dorsal lateral geniculate nucleus. *PLoS Computational Biology* 2011, 7, 1–12.
5. Brunel, N. Dynamics of Sparsely Connected Networks of Excitatory and Inhibitory Spiking Neurons. *Journal of Computational Neuroscience* 2000, 8, 183–208.
6. Tennøe S, Halnes G, Einevoll GT. Uncertainpy: A Python toolbox for uncertainty quantification and sensitivity analysis in computational neuroscience. *bioRxiv* 2018, 274779.

## P228 The emergence of spatiotemporal spike patterns and feature binding relations within a spiking neural network model of the primate visual cortex: a cortical implementation of capsule networks

### James Isbister, Simon Stringer

#### University of Oxford, Department of Experimental Psychology, Oxford, United Kingdom

##### **Correspondence:** James Isbister (isbisterjb@gmail.com)

*BMC Neuroscience* 2018, **19(suppl 2):**P228

The feed forward propagation of visual information in rate-coded neural networks discards information about which low-level features are driving high-level transformation invariant features. In particular, when multiple visual stimuli are present, the network has no way of assigning which low-level features belong to high-level features. Information about the configuration of high-level features, i.e. the spatial composition of the high-level feature in terms of its low-level features, is therefore lost. In visual psychology, this is known as the feature binding problem. Capsule networks [1] demonstrate a new type of artificial neuron called capsules. The activity of a capsule is represented by a vector rather than a “firing rate” value. The magnitude of the vector represents the probability that its preferred feature is present, whilst the direction of the vector represents the configuration of the feature transform. Capsules therefore provide a simultaneous representation of the presence of a feature and its configuration in terms of lower level features, somewhat analogously to feature binding. But these are not a plausible model of brain function. We show how a spiking neural network can give rise to emergent spatiotemporal spike patterns and feature binding representations. An earlier modelling study [2] showed how synchronized activity can emerge over a series of layers. Building on this work, we show how incorporating randomized axonal delays leads to the emergence of spatiotemporal patterns of spikes (polychronization). This is an inductive process over a series of layers. Such spike patterns emerge even when the input neurons have randomized spike times. These spatiotemporal spike patterns carry information relating to the hierarchical binding relations between lower and higher features. Our simulations demonstrate that neurons can learn to respond invariantly over a range of transformations of a high-level feature, whilst simultaneously representing the configuration of the feature transform using the precise timings of their spikes. The relative timings between the spikes of neurons representing a high-level feature vary continuously and monotonically as the high-level feature undergoes transformation. Such a representation may be how the brain forms a similar representation to that of capsules and could be part of the brain’s solution to the feature-binding problem. Our spiking network models can also represent the hierarchical binding relations between lower and higher level features through the emergence of binding neurons [3], which fire if and only if a neuron encoding a lower-level feature is participating in firing a neuron representing a higher-level feature. This implies that the low level feature is part of the high level feature. Such binding neurons develop through visually guided learning with STDP.

**References**

1. Sabour, S. Frosst, N, Hinton GE. Dynamic routing between capsules. In *Advances in Neural Information Processing Systems* 2017 (pp. 3859–3869).
2. Diesmann M, Gewaltig M, Aertsen A. Stable propagation of synchronous spiking in cortical neural networks. *Nature* 1999, 402(6761), 529–533.
3. Eguchi A, Isbister J, Ahmed N, Stringer SM. (in press) The emergence of polychronization and feature binding in a spiking neural network model of the primate ventral visual system, *Psychological Review*, in press.

## P229 Inhibitory plasticity moulding excitatory spatio-temporal receptive fields in a spiking neural network model

### Nasir Ahmad^1^, Kerry Walker^2^, Simon Stringer^1^

#### ^1^University of Oxford, Department of Experimental Psychology, Oxford, United Kingdom; ^2^University of Oxford, Department of Physiology, Anatomy and Genetics, Oxford, United Kingdom

##### **Correspondence:** Nasir Ahmad (nasiryahm@gmail.com)

*BMC Neuroscience* 2018, **19(suppl 2):**P229

Excitatory plasticity has long been the focus of learning in spiking neural networks. From the earliest pairwise Spike-Timing Dependent Plasticity (STDP) rules to triplet STDP rules and beyond, excitatory learning rules have been explored both experimentally and theoretically. Inhibitory plasticity has only more recently been appreciated and shows promise in network stabilisation, homeostasis, and predictive coding [1, 2]. Very recently, investigations into the properties of inhibitory plasticity in decorrelating excitatory responses and shaping excitatory synaptic weights have emerged in both experimental and theoretical studies [3, 4]. This study aims to highlight these effects and make the claim that under a given inhibitory plasticity rule [1], inhibitory spatio-temporal receptive fields play a crucial role in the development of excitatory receptive field structures. Decorrelation of excitatory cells has been identified as a function of inhibitory neurons and plasticity [3]. This description can be misleading. In fact, excitatory neurons in a network with a correlative inhibitory plasticity rule have activity which is decorrelated with their inhibitory input activity. The result is two-fold. First, excitatory receptive fields can only cover those stimuli which are not predicted/explained by their inhibitory inputs. In the case of balance, inhibitory inputs can “explain away” all incoming excitatory stimulation. However, if this balance is incomplete, it leads to excitatory cells responding to only those stimuli which its inhibitory inputs do not cover. The connectivity (and receptive field tuning widths) of inhibitory cells therefore determine the corresponding excitatory cell response characteristics. Curious effects also occur if excitatory and inhibitory cells are active on different input time scales. This is most clear when we consider dynamic stimuli with inhibitory neurons and inhibitory synapses acting on a timescale significantly faster or slower than excitatory cells/synapses. Under these conditions, excitatory cells compete to form a receptive field on the timescale of the incoming inhibition. The excitatory receptive field forms a peak at this timescale (e.g. close to or far from stimulus onset) and learning at all other timescales reflect features that predict activation at that peak.

These effects are studied in a spiking neural network model. In particular, this study shows the emergence of structure in the inhibitory weights (related to the correlation of pre and post synaptic cell responses) and how this affects the emergence of excitatory spatio-temporal receptive fields.

**References**

1. Vogels TP, Sprekeler H, Zenke F, et al. Inhibitory plasticity balances excitation and inhibition in sensory pathways and memory networks. *Science* 2011, 334, 1569–1573.
2. Boerlin M, Machens CK, Denève S. Predictive coding of dynamical variables in balanced spiking networks. *PLoS Comput Biol*. 2013, 9, e1003258.
3. Clopath C, Vogels TP, Froemke RC, Sprekeler H. Receptive field formation by interacting excitatory and inhibitory synaptic plasticity [Internet]. *bioRxiv*. 2016. p. 066589.10.1101/066589
4. Sprekeler H. Functional consequences of inhibitory plasticity: homeostasis, the excitation-inhibition balance and beyond. *Curr Opin Neurobiol.* 2017, 43, 198–203.

## P230 Learning to be modular: Interplay between dynamics of synaptic strengths and neuronal activity in the brain results in its modular connection topology

### Janaki Raghavan^1^, Sitabhra Sinha^2^

#### ^1^University of Madras & The Institute of Mathematical Sciences, Department of Physics, Chennai, India; ^2^The Institute of Mathematical Sciences, Theoretical Physics, Chennai, India

##### **Correspondence:** Janaki Raghavan (janaki.phys@gmail.com)

*BMC Neuroscience* 2018, **19(suppl 2):**P230

Neurons in the brain are connected in intricate arrangements, communicating with each other mostly through chemical synapses. The collective dynamics of neuronal networks—responsible for the cognitive and other functions of the brain—is strongly influenced by their connection topology. Motivated by reports [1, 2] that at least some parts of the brain may be wired in a modular fashion, we first show that the occurrence of modularity promotes the dynamical balance between excitation and inhibition in neuronal networks. In other words, the range of values for the fraction of inhibitory neurons in the network that will result in a high probability of persistent activity is considerably amplified by modular organization of the connection topology. We then explore the possibility that this modularity arises in a self-organized manner when a set of neurons interacting with each other spontaneously organize themselves into densely inter-connected communities with sparser connections to other communities. For this purpose, in parallel with action potential generating models of individual neurons, we investigate spike-time dependent plasticity (STDP) synaptic dynamics [3] for evolving the connectivity organization between neurons. Note that STDP is considered to be the principal mechanism by which neurons strengthen or weaken connections, and thereby “learn” according to the Hebbian paradigm (“neurons that fire together, wire together”). We show that, beginning from a homogeneous globally connected network, the interplay of neuronal dynamics and STDP results in the spontaneous emergence of modules in the network. Furthermore, along with synapses, neurons are also connected through gap junctions in real systems. The precise role played by the gap junctions in a network is not yet fully understood, although it is believed to be important for synchronization and rhythmic activity. We consider a system of neurons with two distinct types of interactions (synapse and gap junctions) and such systems can be better described using a multiplex network [4] (Fig. 1). Using this network paradigm, we aim to uncover new emergent dynamical behaviors that arise due to coexistence of two types of connections and get further insights into how gap-junctions enhance the robustness and efficiency of real neuronal systems in general.

**Fig. 1:**
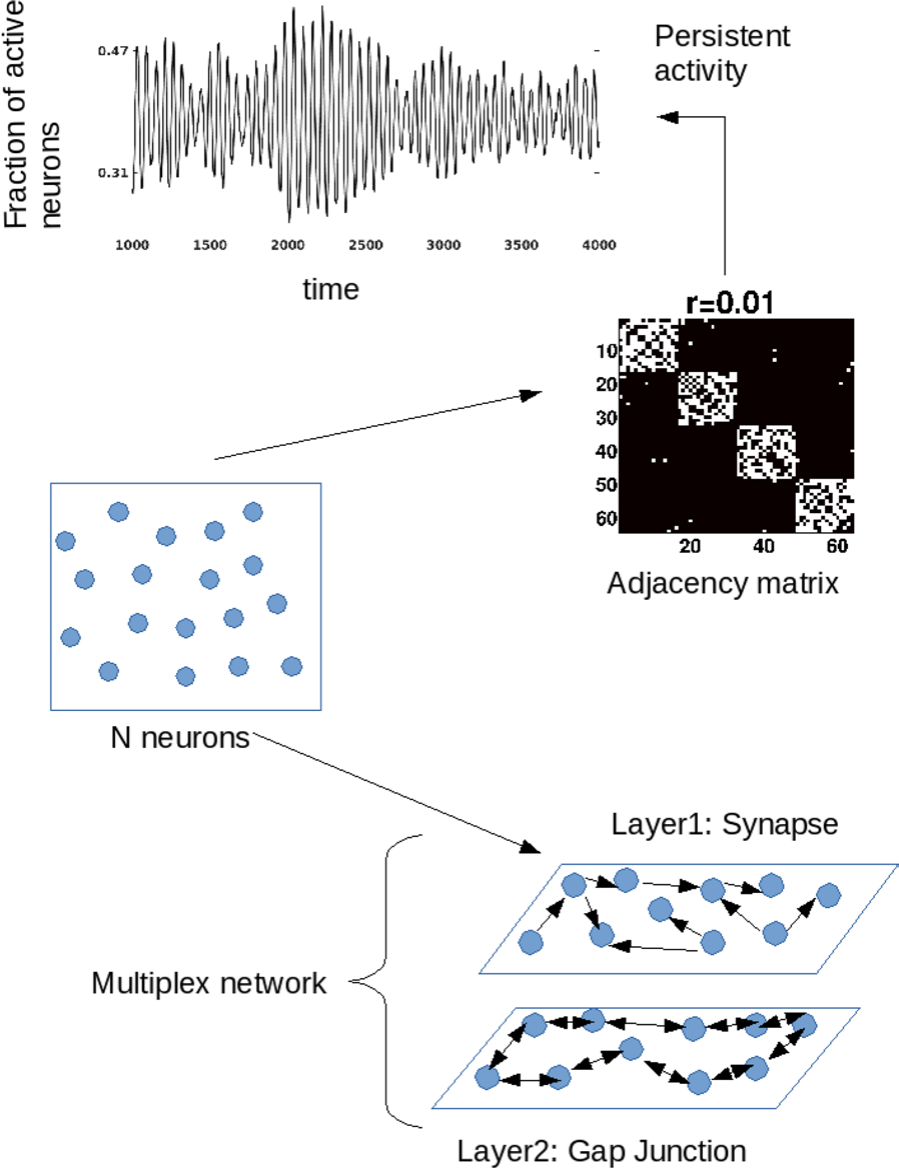
Schematic representation of the neuronal system as studied in this work

**References**

1. Olaf Sporns, Dante R. Chialvo, Marcus Kaiser, Claus C. Hilgetag, Organization, development and function of complex brain networks, *Trends in Cognitive Sciences* 2004, 8, 9.
2. Raj Kumar Pan, Sitabhra Sinha, Nivedita Chatterjee, Mesoscopic organization reveals the constraints governing Caenorhabditis elegans nervous system, 2010, Vol 5, Issue 2, e9240.
3. Guo-qiang Bi and Mu-ming Poo, Synaptic Modifications in Cultured Hippocampal Neurons: Dependence on Spike Timing, Synaptic Strength, and Postsynaptic Cell Type*, J. Neuroscience* 1998, 18 (24) 10464 10472.
4. Federico Battiston, Vincenzo Nicosia and Vito Latora, Structural measures for multiplex networks, *Physical Review* 2014, E 89 (3), 032804.

## P231 Uncovering the mesoscopic organisation of the macaque brain

### Anand Pathak, Shakti N. Menon, Sitabhra Sinha

#### The Institute of Mathematical Sciences, Theoretical Physics, Chennai, India

##### **Correspondence:** Anand Pathak (anandpathak31@gmail.com)

*BMC Neuroscience* 2018, **19(suppl 2):**P231

In order to unravel how the brains of higher organisms carry out cognitive and motor functions, it is crucial to understand the structural organization of the neurons at different levels of hierarchy. At an anatomical level, the mammalian brain is compartmentalized into different regions (lobes, gyri, nuclei etc.) Brain regions, each having millions of neurons, are connected to each other through axonal bundles projecting out from their respective neurons. One approach to study the structural connectome of the brain is to consider the brain network at the scale of brain regions. This approach has become possible to implement in brains of higher mammals, e.g., the macaque monkey, for which a large amount of data on different brain areas and their connectivity have been collated in the online repository CoCoMac. Using as our starting point a previous study [1] that organized the data available from the CoCoMac database of macaque brain connectivity, we have reconstructed an unambiguous and comprehensive brain network with regions covering the entire brain cortex as well as subcortical regions (Fig. 1). Mesoscopic analysis of a network pertains to those substructures that occur at a level much higher than few nodes but at a scale lower than whole network. In a brain network, knowing the mesoscopic organization can characterize its basic structural and functional make up. In particular we consider the modular and hierarchical organization of the macaque brain. Modular networks consist of sub-networks called modules that are densely connected within themselves but have sparse inter-modular connections. A high degree of structural modularity in a brain network reveals functional compartmentalisation and co-ordination among the brain regions. A hierarchical network on the other hand is organized into layers that have dense connections between consecutive layers and relatively sparse connections between non-consecutive layers. Uncovering the hierarchical organization of a brain network not only illuminates the structural and functional compartmentalization but also the directionality of information flow. Analysing these two distinct types of mesoscopic organization of the macaque brain network reveals the larger plan of the macaque brain architecture.

**Fig 1:**
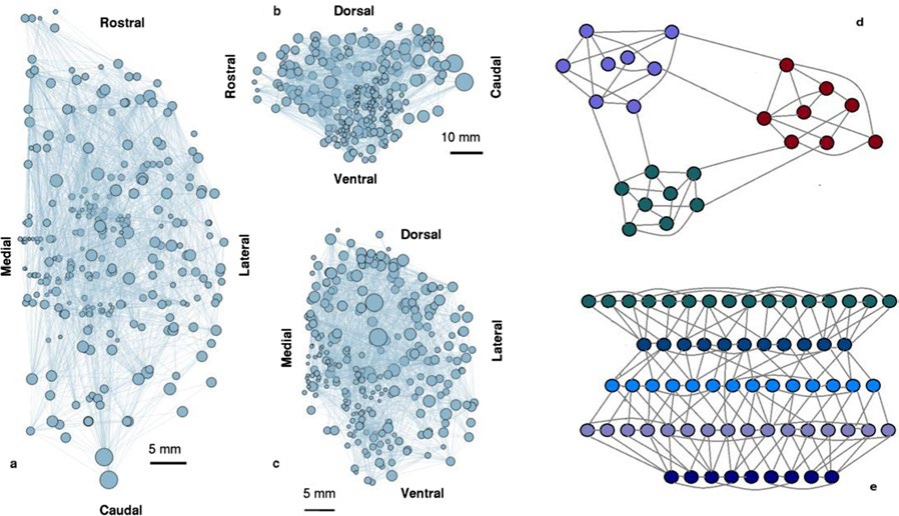
(a) to (c) Spatial representation of the macaque brain network as viewed from three different axes. Nodes represent the brain regions and the links represent connections between brain regions. Their relative positions are according to their actual locations in brain and the circle sizes are proportional to actual volumes of the respective brain areas. (d) Schematic representation of modular organisation in a network. (e) Schematic representation of hierarchical organisation in a network

Our analysis reveals that the macaque brain network exhibits a highly modular structure, which in spite of being in accordance with the known functional organization, provides interesting new insights and implications about the functioning of many less studied brain regions. The arrangement of modules is spatially contiguous except one saliently fragmented module that is particularly intriguing and suggestive in its functionality. This study strongly suggests that even though macaque brain connectivity is clearly governed by the spatial configuration of the brain regions, the modular structure is essentially independent of the geometry and shape of the brain, and hence the emergence of modules seems to be a more fundamental attribute. An even more surprising observation is that the macaque brain network also has a highly hierarchical structure. Our new original approach for determining the hidden hierarchical structures in a network opens up a whole range of possible analysis to further understand the structure and function of neuronal networks in general.

**Reference**

1. Modha D, Singh R. Network architecture of the long-distance pathways in the macaque brain. *PNAS* 2010, 107(30) 13485–13490

## P232 Multimodal modeling of neural network activity: computing LFP, ECoG, EEG and MEG signals with LFPy2.0

### Espen Hagen^1^, Torbjørn V Ness^2^, Gaute Einevoll^2^, Solveig Næss^3^

#### ^1^University of Oslo, Department of Physics, Oslo, Norway; ^2^Norwegian University of Life Sciences, Faculty of Science and Technology, Ås, Norway; ^3^University of Oslo, Department of Informatics, Oslo, Norway

##### **Correspondence:** Espen Hagen (espen.hagen@fys.uio.no)

*BMC Neuroscience* 2018, **19(suppl 2):**P233

Recordings of extracellular electrical, and later also magnetic, brain signals have been the dominant technique for measuring brain activity for decades. The interpretation of such signals is however nontrivial [1], as the measured signals result from both local and distant neuronal activity. In volume-conductor theory the extracellular potentials can be calculated from a distance-weighted sum of contributions from transmembrane currents of neurons. Further, given the same transmembrane currents, the contributions to the magnetic field recorded both inside and outside the brain can also be computed [2]. This allows for the development of computational tools implementing forward models grounded in the biophysics underlying the different measurement modalities [1]. LFPy ([3], LFPy.github.io) incorporated a now well-established scheme for predicting extracellular potentials of individual neurons with arbitrary levels of biological detail. It relies on NEURON ([4], neuron.yale.edu) to compute transmembrane currents of multicompartment neurons which is then used in conjunction with an electrostatic forward model [5]. We have now extended its functionality to populations and networks of multicompartment neurons with concurrent calculations of extracellular potentials and current dipole moments. The current-dipole moments are used to compute non-invasive measures of neuronal activity, like magnetoencephalographic (MEG) signals [2, 6] and, when combined with an appropriate head-model, electroencephalogram (EEG) scalp potentials. One such built-in head-model is the 4-sphere model including the different electric conductivities of brain, cerebral spinal fluid, skull and scalp [6, 7]. The version of LFPy presented here is thus a true multi-scale simulator, capable of simulating electric neuronal activity at the level of cell-membrane dynamics, individual synapses, neurons, networks, extracellular potentials within neuronal populations and macroscopic EEG and MEG signals. The present implementation is equally suitable for execution on laptops and in parallel on high-performance computing (HPC) facilities. The code is free, open source, and available from GitHub (https://github.com/LFPy/LFPy).

**References**

1. Einevoll GT, Kayser, C, Logothetis NK, Panzeri S. Modelling and analysis of local field potentials for studying the function of cortical circuits. *Nat Rev Neurosci* 2013. 14:770–785. 10.1038/nrn3599
2. Hämäläinen M, Hari R, Ilmoniemi RJ, Knuutila J, Lounasmaa OV. Magnetoencephalography—theory, instrumentation, and applications to noninvasive studies of the working human brain. *Rev Mod Phys* 1993. 65:413–487. 10.1103/revmodphys.65.413
3. Lindén H., Hagen E., Leski S., Norheim E., Pettersen K., Einevoll GT. LFPy: a tool for biophysical simulation of extracellular potentials generated by detailed model neurons. *Front Neuroinform* 2014, 7(41):1–15. 10.3389/fninf.2013.00041
4. Hines M, Davison A, Muller E. NEURON and Python. *Front Neuroinform* 2009. 3(1):1–12. 10.3389/neuro.11.001.2009
5. Holt G, Koch C. Electrical Interactions via the Extracellular Potential Near Cell Bodies. *J Comp Neurosci* 1999. 6:169–184. 10.1023/a:100883270
6. Nunez PL & Srinivasan R. *Electric Fields of the Brain*. Oxford University Press 2006. ISBN: 9780195050387
7. Næss S, Chintaluri C, Ness TV, Dale AM, Einevoll GT, Wójcik DK. Corrected Four-Sphere Head Model for EEG Signals. *Front Hum Neurosci* 2017. 11:490. 10.3389/fnhum.2017.00490

## P233 Quantitative comparison of a mesocircuit model with motor cortical resting state activity in the macaque monkey

### Michael von Papen^1^, Nicole Voges^1^, Paulina Dabrowska^1^, Johanna Senk^1^, Espen Hagen^2^, Markus Diesmann^1^, David Dahmen^1^, Lukas Deutz^3^, Moritz Helias^1^, Thomas Brochier^3^, Alexa Riehle^3^, Sonja Gruen^1^

#### ^1^Jülich Research Centre, Institute of Neuroscience and Medicine (INM-6) and Institute for Advanced Simulation (IAS-6), Juelich, Germany; ^2^University of Oslo, Department of Physics, Oslo, Norway; ^3^CNRS - Aix-Marseille Université, Institut de Neurosciences de la Timone (INT), Marseille, France

##### **Correspondence:** Michael von Papen (m.von.papen@fz-juelich.de)

*BMC Neuroscience* 2018, **19(suppl 2):**P234

Modeling studies of cortical network dynamics frequently aim to include realistic assumptions on structural and effective connectivity [4, 6] to achieve a qualitative reproduction of experimentally observed neuronal activity. Here, we develop a quantitative validation approach where mean-field theory [2] guides the adaptation of a generic point-neuron network model to macaque motor cortex. We describe the characteristics of the experimental data extracted and used for comparison and present preliminary results for the generic network model. The underlying network model is an upscaled version of the Potjans &Diesmann [4] layered spiking network model extended to a size of 4x4mm2and a total of ~ 1.2 million leaky integrate-and-fire neurons [3]. In contrast to the original model this mesocircuit model uses lateral distance-dependent connection probabilities derived from cortical neuroanatomical data. To compare the output with observations we subsample single unit activities from the corresponding layer in the simulated network with the same number of neurons and with the same spatial arrangement of the recording array as in the experimental data. The model describes a system in ground, idle or resting state with uncorrelated input. In order to perform a quantitative comparison with experimental data we therefore conducted a resting state experiment with macaque monkeys not given any specific task or stimulus. We recorded neuronal activity from premotor and motor cortex using a chronically implanted 4x4mm2Utah array with 100 electrodes [1, 5]. A video of the monkey was used to differentiate between periods of rest and spontaneous movements.

The experimental single unit activities (~ 140 neurons) are subdivided into putative excitatory and inhibitory neurons based on their spike widths. We find that a) putative inhibitory and excitatory activity is in a balanced state, b) spike counts increase during movement, c) inhibitory units contribute more strongly to firing rate modulations than excitatory units, d) they also tend to be more strongly correlated among each other and e) the dimensionality of cortical activity is decreased during movement. Our results are to a large degree in accordance with mean-field theoretic predictions and may thus allow us to infer constraints on the parameter space of the mesocircuit model.

**References**

1. Brochier T, et al. Massively parallel recordings in macaque motor cortex during an instructed delayed reach-to-grasp task. (Data publication) *Scientific Data* 2018(accepted)
2. Dahmen D, et al. Two types of criticality in the brain, *arXiv* 2017:1711.10930 [cond-mat.dis-nn]
3. Hagen E, et al. Local field potentials in a 4 × 4 mm2multi-layered network model. CNS-2016, *BMC Neurosci*. 2016, 17(Suppl 1)
4. Potjans, Diesmann M. The cell-type specific cortical microcircuit: Relating structure and activity in a full-scale spiking network model. *Cereb. Cort*. 2014, 24(3)
5. Riehle A, et al. Mapping the spatio-temporal structure of motor cortical LFP and spiking activities during reach-to-grasp movements. *Front. Neural Circuit* 2013, 7(48)
6. Voges N, Perrinet. Complex dynamics in recurrent cortical networks based on spatially realistic connectivities, *Front. Comput. Neurosc*. 2012, 6.

## P234 Generalized phase resetting and phase-locked mode prediction in biologically-relevant neural networks

### Dave Austin, Sorinel Oprisan

#### College of Charleston, Department of Physics and Astronomy, Charleston, SC, United States

##### **Correspondence:** Dave Austin (austindi@g.cofc.edu)

*BMC Neuroscience* 2018, **19(suppl 2):**P235

Environment stimuli are continuously processed by the central nervous system (CNS) to better adjust, adapt, and learn new responses that optimize our benefits. At neural level, the external stimuli are coded as spikes of electric activity, called action potentials (APs). Neurons respond to changes in the environment by altering their firing speed, or phase, which means that instead of firing at a regular pace the neuron starts firing faster or they slow down. The amount of change, or resetting, in their firing period is determined by the timing, duration, and strength of the external stimulus. However, neurons connect with each other and create large networks capable of elaborated firing patterns that drive the response of the organism. We modeled the neural network as hierarchically-organized layers of neurons and in each layer the neurons’ response is dictated by its own phase resetting behavior. We successfully generalized mathematically and then checked numerically that knowledge of how one isolated neuron responds to a stimulus can help predicting the response of a larger network to complex stimuli.

## P235 Recruitment of neurons into neural ensembles based on dendritic plateau potentials

### Peng Gao^1^, Joe Graham^2^, Sergio Angulo^2^, Salvador Dura-Bernal^2^, Michael Hines^3^, William W Lytton^2^, Srdjan Antic^4^

#### ^1^UCONN Health, Department of Neuroscience, Farmington, CT, United States; ^2^SUNY Downstate Medical Center, Department of Physiology and Pharmacology, Brooklyn, NY, United States; ^3^Yale University, Department of Neuroscience, CT, United States; ^4^University of Connecticut Health Center, Department of Neuroscience, Farmington, CT, United States

##### **Correspondence:** Peng Gao (penggao.1987@gmail.com)

*BMC Neuroscience* 2018, **19(suppl 2):**P236

Prefrontal cortex plays a crucial role in advanced cognitive functions. Previous experimental observation has shown that glutamatergic inputs to the basal dendrites of cortical pyramidal neurons activate AMPA and NMDA receptors which can bring the dendrites into a long-lasting depolarized state: a dendritic plateau potential. These sustained depolarizations push the cell body towards spike threshold and reduce the membrane time constant. In such a “Prepared” state, the pyramidal cells can respond to other sparse synaptic inputs more quickly and easily, facilitating synchronization of firing. During the plateau depolarization, a neuron can tune into ongoing network activity and synchronize spiking with other neurons to provide a coordinated “Active” state (robust firing of somatic action potentials), which would permit “binding” of signals through coordination of neural activity across a population. Under this scenario, Active cells are recruited from cells in the Prepared state, and therefore the transient Active ensemble is embedded in the longer-lasting Prepared ensemble of neurons. We hypothesize that “embedded ensemble encoding” may be an important organizing principle in networks of neurons, explaining how electrical signaling endows central nervous system with capacity to form large number of neural ensembles. Also, embedded ensemble encoding pulls together two concepts (rate coding vs. temporal coding) that are typically seen to be in opposition. We have developed a morphologically-detailed model reconstructed from a cortical Layer 5 prefrontal pyramidal neuron in the NEURON simulator. Both synaptic AMPA/NMDA and extrasynaptic NMDA inputs are placed on basal dendrites to model the induction of plateau potentials (Fig. 1a–d). The active properties of the cell are tuned to match the amplitude and duration of experimentally observed plateau potentials utilizing voltage-sensitive dyes in dendrites and whole-cell patch recording in soma (Fig. 1e). In addition, the effects of input location, receptor conductance, calcium-activated potassium channels and voltage-activated calcium channels are explored in detail. These findings help us to better understand the implications of dendritic plateaus at the cellular and network level. In the future, this detailed individual cell model can be used to develop cortical meso-scale network models for exploring the hypotheses pertaining to the recruitment of neurons into neural ensembles.

**Fig. 1:**
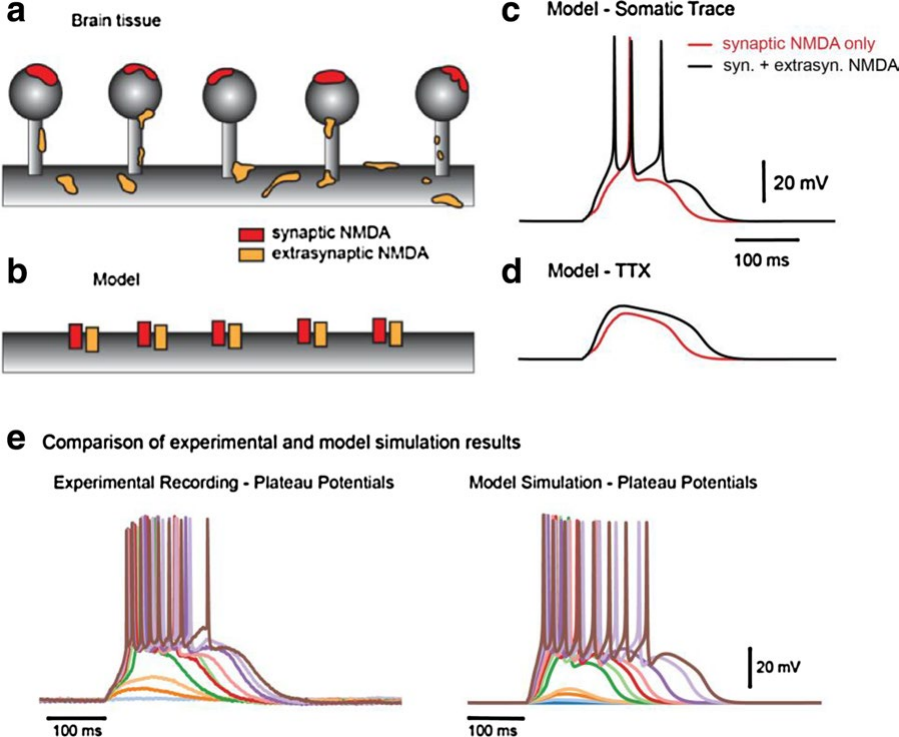
Modeling of the glutamate evoked dendritic plateau potentials

## P236 Integrating large brain networks and network analysis to understand the epileptogenic zone

### Adam Li^1^, Marmaduke Woodman^2^, Viktor Jirsa^2^, Sridevi Sarma^1^

#### ^1^Johns Hopkins University, Biomedical Engineering, Baltimore, CA, United States; ^2^Aix-Marseille Universite, Institute de Neurosciences, Marseille, France

##### **Correspondence:** Adam Li (adam2392@gmail.com)

*BMC Neuroscience* 2018, **19(suppl 2):**P237

Over 20 million people in the world suffer from medically refractory epilepsy (MRE). Approximately 50% of MRE patients have focal MRE, meaning that a small focal region in the brain, the epileptogenic zone (EZ), is the source of the seizures. For patients with focal MRE, treatment by surgical resection of the EZ can be effective, provided the EZ is reliably identified and entirely removed. Identification of the EZ often requires a surgical implantation of subdural grid or stereotactic depth electrodes (SEEG) electrodes, followed by a visual inspection of hundreds of EEG signals during seizure events that occur over several days to weeks. Clearly, surgical outcome relies heavily on precise localization of the EZ. We aim to integrate efforts from computational modeling and data analysis of SEEG recordings to better localize the EZ in an epilepsy patient. The Virtual Brain (TVB) is a computational platform that can integrate patient-specific information, such as brain connectivity derived from MRI and clinician’s EZ hypotheses, to form personalized brain models capable of simulating realistic functional signals (i.e. SEEG). From a data analysis perspective, we used a novel network-based algorithm, coined the “fragility algorithm”, that has demonstrated capabilities of localizing the EZ by analyzing network stability with respect to nodal perturbations. The fragility algorithm, unlike single channel frequency analysis, looks at the SEEG as a network and can efficiently analyze a SEEG network to create a heatmap of predictions on the EZ. The fragility algorithm determines which nodes within the epileptic network (i.e. SEEG channel) are the most fragile, i.e., nodes that if connections from it are perturbed slightly will destabilize the network. Fragility weights for each node are then used to predict the EZ. We built personalized brain models for two temporal focal MRE patients to determine the algorithm’s predictions in two different situations. We simulated for each patient in silico: (1) inside: placement of the EZ at the clinical hypothesis and (2) outside: placement of the EZ outside the resection region. We then applied the fragility algorithm on the simulated and actual SEEG data from each patient to see if the fragility maps of the simulated EZ scenarios resemble the fragility maps derived from actual recordings. With TVB integrated with fragility analysis, we can hypothesize where the true EZ might be for a given MRE patient and whether or not it was correctly identified by clinicians using standard visualization methods. In one patient who had a successful surgery, we assume the EZ lies within the resected region and we found that the predicted EZ in the real SEEG and simulated data as identified by the algorithm, matched the clinically annotated EZ. In contrast, the patient with failed surgery, we assume the EZ lies outside the resected region and we found that the predicted EZ in the real SEEG data and the simulated data does not match the clinical EZ. These results suggest that the failed epilepsy surgery was due to the fact that the EZ was not within the resected region, while in the success case it was. These results outline how personalized brain models can help determine sensitivity of EZ localization algorithms to locations of the EZ and it can integrate with data analysis to validate whether the EZ is properly localized in a surgical resection.

## P237 A high resolution data-driven model of the mouse connectome

### Joseph Knox^1^, Kameron Decker Harris^2^, Nile Graddis^1^, Jennifer Whitesell^1^, Julie Harris^1^, Hongkui Zeng^1^, Eric Shea-Brown^3^, Stefan Mihalas^1^

#### ^1^Allen Institute for Brain Science, Modelling, Analysis and Theory, Seattle, WA, United States; ^2^University of Washington, Department of Computer Science, Seattle, WA, United States; ^3^University of Washington, Department of Applied Mathematics, Seattle, WA, United States

##### **Correspondence:** Joseph Knox (josephk@alleninstitute.org)

*BMC Neuroscience* 2018, **19(suppl 2):**P238

Knowledge of mesoscopic brain connectivity is important in understanding inter-region communication and information processing. Models of structural connectivity have been used to investigate the relationship with functional connectivity, to compare brain structures across species, and more [5, 2, 4, 6]. Early models of connectivity were constructed with the assumption that the regions are homogeneous [3]. This assumption is useful, but it depends on predefined regional parcellations and describe connectivity at a region-limited level of resolution. Here, we go beyond the regional approach and construct a model of the whole brain connectivity at the scale of 100 micron voxels, extending the previous work of [1]. We use the Allen Mouse Brain Connectivity Atlas, a large scale dataset measuring with two photon tomography the projections of sets of neurons infected with a viral tracer to approximately 5x10^5 target voxels [3]. While this dataset is large, the 429 sources we have in wild type C57BL/6 mice pale in comparison to the 2.5 × 10^5 source voxels (all experiments were conducted in one hemisphere). To meet this challenge, we propose a model which relaxes the assumption of homogeneity of connections within a region and instead assumes smoothness across major brain divisions. It is a simplification of the structured regression model proposed in [1], which outperformed a regional model in the visual cortex. We model the connectivity at each source voxel as the kernel-weighted average of the projection patterns of nearby injections (Fig. 1). The kernel is a radial basis function with bandwidth hyperparameter fit in the inner loop of a nested K-fold cross validation procedure. The voxel-scale model strongly outpredicts the previous regional modeling both in relative mean squared error in cross-validation, and when compared to a human-curated dataset. We plan to release the code to perform construct this network. As our model performs an interpolation, it inherently produces a dense connectivity. While the true sparsity of the mouse connectome is debated [7], a manual analysis of true positive and true negative signals in the data [3], poses the question of the relevance of very weak connections. Thus we plan to use this model to examine the whole brain distribution of connection weights, and the importance of very weak connections on common graph theoretical models.

**Fig. 1:**
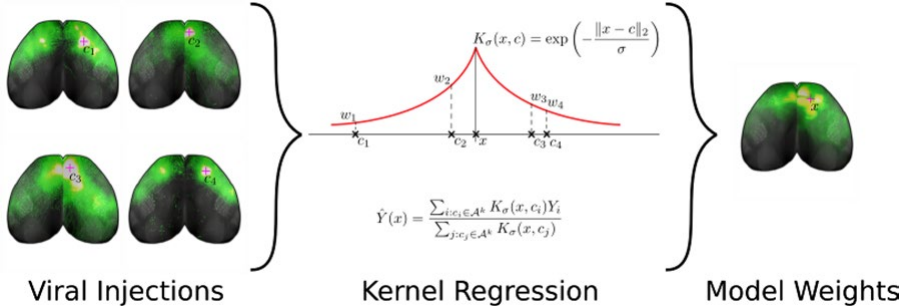
An illustration of the voxel-scale model projected into a cortical surface view. We predict the connectivity at each voxel in the brain using a kernel weighted average of the injections in each major brain division

The new voxel-scale model has been used in several applications including analysis of the modularity of the mouse cortical network. This new voxel-scale model of the mouse connectome permits researchers to extend their previous analyses of structural connectivity to unprecedented levels of resolution, and allows for natural comparison with functional imaging and other datasets.**References**

1. Harris KD, Mihalas S, Shea-Brown E. High resolution neural connectivity from incomplete tracing data using nonnegative spline regression. *In Proc. NIPS* 2016
2. Laramée ME, Boire D. Visual cortical areas of the mouse: comparison of parcellation and network structure with primates. *Frontiers in Neural Circuits* 2015
3. S. W. Oh et al. A mesoscale connectome of the mouse brain. *Nature* 2014
4. S. S. Sethi et al. Structural connectome topology relates to regional BOLD signal dynamics in the mouse brain. *Chaos* 2017
5. J. M. Stafford et al. Immunosignature system for diagnosis of cancer. *Proceedings of the National Academy of Sciences* 2014
6. Wang XJ, Kennedy H. Brain structure and dynamics across scales: in search of rules. *Current Opinion in Neurobiology* 2016
7. R. Gămănuţ et al. The Mouse Cortical Connectome, Characterized by an Ultra-Dense Cortical Graph, Maintains Specificity by Distinct Connectivity Profiles. *Neuron* 2018

## P238 Convolutional neuronal networks with extra-classical receptive fields

### Brian Hu, Stefan Mihalas

#### Allen Institute for Brain Science, Modelling, Analysis and Theory, Seattle, WA, United States

##### **Correspondence:** Brian Hu (brianh@alleninstitute.org)

*BMC Neuroscience* 2018, **19(suppl 2):**P238

Convolutional neuronal networks have had great success in many applications [1] as well as in describing neural responses in the ventral stream of primates [2]. However such networks require labeled data to train and are quite brittle: a single pixel change can with high confidence change the category prediction of the classifier [3]. In contrast to what is known from biology, these networks focus on feedforward visual processing, largely ignoring the influence of lateral connections. They also focus on supervised rather than unsupervised learning. Can we use knowledge from biology to improve the robustness of these systems by including recurrent lateral connections learned in an unsupervised manner? Visual processing in the brain makes use of recurrent lateral connections. Surround suppression, contour integration, and figure-ground segmentation are all examples of forms of contextual modulation that cannot be simply explained by feedforward mechanisms, and are often included as part of the extra-classical receptive field. Experimental and modeling studies suggest different excitatory and inhibitory cell types are important for mediating these lateral connections. The brain is also able to learn structured representations in a largely unsupervised manner with little to no labeled data. Here, we explore how representations learned in an unsupervised way can be used in conjunction with features learned in a supervised manner to improve the robustness of convolutional neuronal networks. We leverage recent work [4] demonstrating how optimal lateral connections can be learned through a modified Hebbian learning rule. We combine optimal context integration with deep learning to not only learn different features within a convolutional neuronal network, but also the optimal lateral connections between the neurons encoding these features. These connections can be implemented using a collection of cell types and lateral connections which match well biological observations in cortical circuits. We demonstrate the influence of lateral connections by testing our model on two standard image datasets: MNIST and CIFAR-10. In additional to the original images, we also generate noisy versions of the images by adding additive white gaussian noise and salt-and-pepper noise in increasing levels. These noisy images are used to test the robustness of the trained convolutional neural networks. In contrast to traditional deep learning approaches, we combine supervised and unsupervised training in our model. We then test models with and without optimal lateral connections on the original images in each dataset, as well as on the noise-corrupted images. As expected, increasing levels of noise degrades model performance for both the MNIST and CIFAR-10 datasets; however, models with optimal lateral connections are more robust to this noise and can achieve higher classification accuracies on both datasets, especially at higher noise levels. This result demonstrates the potential usefulness of combining supervised and unsupervised learning techniques in real-world vision tasks. Although the natural scene statistics of MNIST and CIFAR-10 differ greatly, the same unsupervised learning rule worked for both systems. Our results suggest that the integration of lateral connections into convolutional neural networks is an exciting avenue of future research (Fig. 1).

**Fig. 1:**
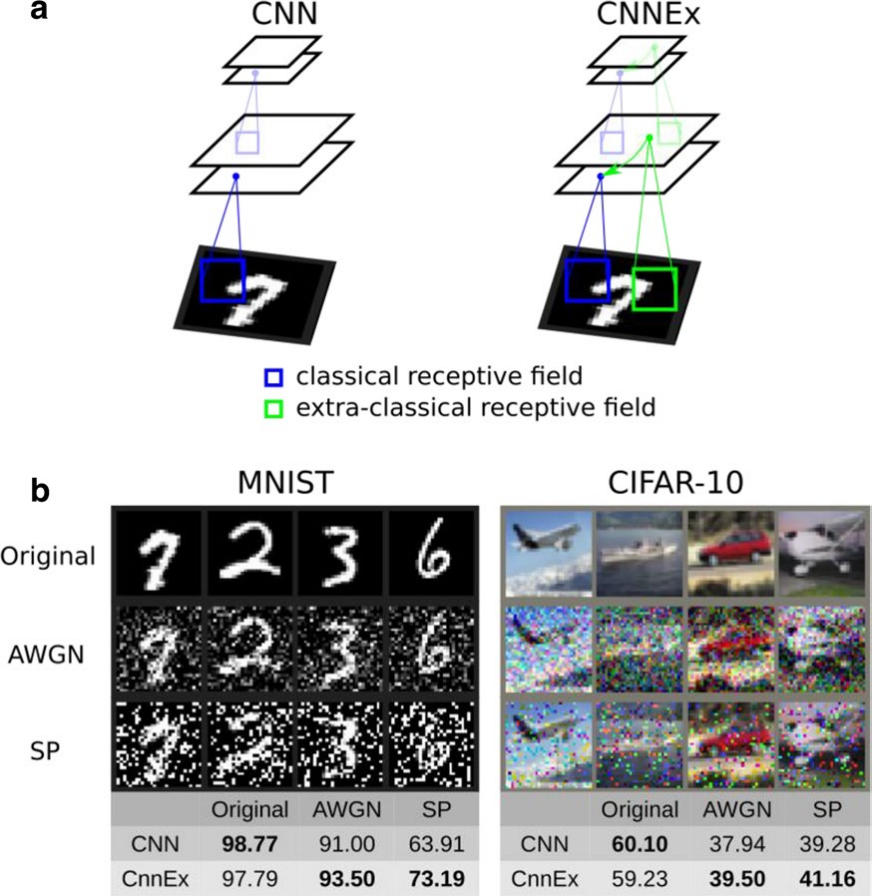
Convolutional neuronal networks with extra-classical receptive fields (CNNEx). (a) Schematic of network architectures with and without extra-classical receptive fields, termed CNNEx and CNN respectively. The classical receptive field is shown in blue and the extra-classical receptive field is shown in green. In CNNEx, neurons from the extra-classical receptive field can modulate the activity of other neurons via recurrent lateral connections learned in an unsupervised manner. (b) The MNIST and CIFAR-10 datasets used to evaluate our models. Example images, along with images corrupted with additive white gaussian noise (AWGN) and salt-and-pepper noise (SP) are shown. The tables below each set of images correspond to model performance (percent accuracy) on the different image sets

## P239 Cortical circuits implement optimal context integration

### Ramakrishnan Iyer, Stefan Mihalas

#### Allen Institute for Brain Science, Modelling, Analysis and Theory, Seattle, WA, United States

##### **Correspondence:** Ramakrishnan Iyer (rami@alleninstitute.org)

*BMC Neuroscience* 2018, **19(suppl 2):**P239

Neuronal responses in early visual cortex are primarily driven by inputs to the classical receptive field and are influenced by stimuli in the receptive field surround. This type of spatial contextual effect is thought to arise due to the statistical structure present in natural scenes, with the surround providing context for the information in the classical receptive field. Lateral connections between neurons in the same cortical area are generally thought to be responsible for transmitting information in the near surround. In a number of recent experimental studies, excitatory neurons have been demonstrated to have like-to-like connectivity with neurons coding for the same feature (e.g. orientation) preferentially connecting to each other with higher probability and/or strength and specific rules for connectivity of inhibitory neuron types have been described. On the other hand, normative models of lateral interactions, relying on sparsity and saliency in the optimal representation of natural images, predict functional inhibition between excitatory neurons. Starting from the assumption that each excitatory neuron represents the probability of a feature being present in the sensory stimulus, we hypothesize that lateral connections serve to optimally (in a Bayesian sense) integrate evidence from the surround. We show that such optimal integration of contextual information can be implemented by a neuronal network. Using natural scene statistics obtained from the Berkeley Segmentation DataSet and in vivo electrophysiological data from awake mouse V1 neurons, we compute the synaptic weights between neurons in our network resulting from the optimal integration of contextual information. We show that this network has like-to-like connectivity between excitatory neurons, in agreement with experimental observations. However, in our model, even neurons with non-overlapping classical receptive fields can have strong connections if they code for features which often co-occur in natural scenes. The distance dependence of connections is similar to those observed experimentally and is found to be heavier-tailed than an exponential decay. These results generalize to other classes of receptive fields including gabors and sharp on–off band-like receptive fields. The network also needs multiple types of inhibition - local normalization, surround inhibition and gating of inhibition from the surround - which we map to the parvalbumin (PV), somatostatin (SST and vasoactive intestinal peptide (VIP) expressing interneuron cell classes respectively. We compared our local circuit model with a correlation-based model, in which the lateral connectivity between cortical neurons is determined by the correlation between their classical receptive fields. We find that the correlation-based model results in a much steeper decay of both like-to-like connectivity and distance dependence, compared to our model. We also show that, compared to a feedforward network, the presence of this local network structure increases the capacity to reconstruct a natural scene from the activities of neurons in the network under noisy conditions. We hypothesize that optimal integration of context is a general computation of cortical circuits, and the local network rules constructed for mouse V1 generalize to other areas and species.

## P240 Identifying the constraints and redundancies shaping the retinal code with a deep network simulation

### Jack Lindsey, Surya Ganguli, Stephane Deny

#### Stanford University, Department of Applied Physics, Stanford, CA, United States

##### **Correspondence:** Jack Lindsey (jacklindsey@stanford.edu)

*BMC Neuroscience* 2018, **19(suppl 2):**P240

Retinal ganglion cells, which transmit the output of the retina to the brain, typically have a concentric center-surround receptive field and consist of ON and OFF types. Although the retinal responses to natural [3] and artificial [1] scenes have been well characterized, it remains unclear how efficient the retinal code is at transmitting visual information. Here we seek to identify the biological constraints of the visual system and the redundancies in natural scene statistics that have shaped the retinal code, by varying respectively the architectural constraints of and statistics of inputs to artificial neural networks trained to classify objects in natural images. We find that when we allow an overcomplete representation in the early layers (i.e. many more neurons in each layer than pixels in the image), the trained network exhibits oriented receptive fields in these layers. However, when we severely restrict the number of neurons in these early layers (guided by the intuition that the optic nerve can only contain a limited number of fibers), concentric center-surround receptive fields emerge. We also find that the response patterns of these neurons naturally cluster in two functional groups analogous to ON and OFF cells in the retina. Moreover, the receptive fields of the first overcomplete layers downstream of our artificial retina are oriented, like receptive fields of V1. Examining the connection weights between the two layers, we find that these oriented receptive fields are generated by drawing from a few center-surround neurons along an axis, mirroring Hubel and Wiesel’s hypothesis about simple cells in visual cortex. These results suggest that the retinal code, with its two main cell populations and its concentric receptive fields, could be optimized to transmit relevant visual information to the brain with a limited number of neurons. This interpretation of the constraints underlying the retinal code is an alternative to that of Karklin and Simoncelli [2], which suggests that the retinal code is optimized for metabolic and noise constraints. Finally we provide evidence that the utility of center-surround encoding for compression arises from covariances among orientation-selective neuron activations in response to natural scenes.

**References**

1. Baden T, Berens P, Franke K, et al. The functional diversity of retinal ganglion cells in the mouse. *Nature* 2016, 529(7586):345.
2. Karklin Y, Simoncelli EP. Efficient coding of natural images with a population of noisy linear-nonlinear neurons. *In Advances in neural information processing systems* 2011, 999–1007.
3. McIntosh L, Maheswaranathan N, Nayebi A, et al. Deep learning models of the retinal response to natural scenes. *In Advances in neural information processing systems* 2016, 1369–1377.

## P241 Biophysical modeling of human MEG reveals two mechanisms effected by bandlimited transients in perceiving weak stimuli

### Robert Law, Hyeyoung Shin, Shane Lee, Christopher Moore, Stephanie Jones

#### Brown University, Department of Neuroscience, Providence, RI, United States

##### **Correspondence:** Robert Law (nosimpler@gmail.com)

*BMC Neuroscience* 2018, **19(suppl 2):**P241

Bandlimited power in cerebral cortex occurs in the form of transient events, a fact often obscured by spectral averaging in classical empirical studies of brain rhythms. Events in the beta band (15–29 Hz) may be locally rhythmic but are brief and sparse, while occurring in scalp-level and mesoscale electrophysiology across diverse neocortical regions in humans, nonhuman primates and rodents. Beta is implicated in nearly every aspect of cortical function, varying during attention, perception and decision-making as well as motor control. However, we presently lack a*mechanistic*model that might afford beta events a functional role susceptible to pharmacological, electromagnetic or genetic controls. We are motivated by studies in primary somatosensory cortex (SI), where beta is consistently reported to have suppressive effects on the perception of subsequent tactile stimulation—even a full second after an event occurs. To investigate the underlying mechanisms, we use a geometrically reduced biophysical circuit model consisting of motifs universal to neocortex, which fits both perceptual and mean magnetoencephalographic (MEG) tactile evoked responses (ERs) after event occurrence in SI, while offering unprecedented predictivity of ER features. Previous modeling indicates that beta events are generated by calcium-mediated bursts incident on L1 with one primary source in nonlemniscal thalamus. Recent studies show that such inputs act simultaneously on superficial pyramidal and interneuron subnetworks. In our model, incoming bursts prime pyramidal dendrites while recruiting neurogliaform (NGF) cells either directly or through electrical coupling after interneuron synchronization. Modeling shows how both mechanisms act in sequence, with NGF suppression occurring more often due to the slow timescale of GABABinhibition (> 250 ms), explaining the net behavioral effect. Before NGF is recruited, however, incoming bursts*amplify*stimuli through subthreshold facilitation - similar to previous oscillation models but with significant improvements in precision. Our model predicts the correct beta phase at stimulus onset in “hit” trials, and also makes two precise predictions of poststimulus bandlimited phase coherences verified at 160–200 Hz at 40 ms (from model L2/3) and 90–110 Hz at 60 ms (from model L5). After this priming phase, L2/3 NGF cells act through GABAB channels on pyramidal somata (in L2/3) and on L5 middle apical dendrites. This mutes L5 pyramidal bursts and clamps L2/3 pyramidal somatic voltages to the potassium reversal. The latter effect is essential for a conspicuous ER feature generated by a completely novel mechanism, where high-voltage downward-propagating dendritic spikes collide with a soma fixed at its minimum voltage. This causes a biophysically maximal local current ideal for driving signaling cations into the soma, raising the possibility that GABAB suppression gates learning after early-prediction error.

Our modeling circumscribes nearly the entire known phenomenology on beta, generating predictions verified at the scalp level with invasively testable analogues. In the process, we unite previously unlinked findings among theoretical, electrophysiological, and anatomical domains. Our results shed particular light on neurogliaform GABABaction in neural computation, with concrete manipulations accessible through a suggestive pharmacopeia including alcohol, opiates, serotonin and neuropeptide Y.

## P242 Multiplexed coding using differentially synchronized spikes: Part 2, experiments

### Dhekra Al-Basha^1^, Milad Lankarany^1^, Stephanie Ratté^1^, Steve Prescott^2^

#### ^1^The Hospital for Sick Children, Neurosciences and Mental Health, Toronto, Canada; ^2^University of Toronto & The Hospital for Sick Children, Neurosciences and Mental Health & Dept. Physiology, Institute of Biomaterials and Biomedical Eng, Toronto, Canada

##### **Correspondence:** Dhekra Al-Basha (dhekra.al.basha@mail.utoronto.ca)

*BMC Neuroscience* 2018, **19(suppl 2):**P242

In our companion poster, Multiplexed coding using differentially synchronized spikes: Part 1, theory and simulations, we demonstrated in silico that the rate of asynchronous spikes can encode the intensity of a slow signal while the timing of synchronous spikes simultaneously encodes abrupt changes in stimulus intensity. This suggests that a single set of neurons can represent distinct features of an external stimulus using differentially synchronized spikes. Here we tested in vivo whether this occurs in real neurons. To this end, we recorded extracellularly from single units in the primary somatosensory cortex of sedated rats. Computer-controlled mechanical stimuli were applied to the whisker pad as discrete steps of increasing force. We asked whether we could decode (1) the force during the sustained phase of the step based on the rate of asynchronous spiking and (2) the timing of the onset and offset of the step based on the timing of synchronous spikes. Using responses from 17 neurons deemed to be responsive to our mechanosensory input, we constructed a firing rate histogram (FRH) in two ways (Fig. 1). By constructing the FRH with a broad Gaussian kernel (500 ms), we found that the magnitude of the firing rate tracks the intensity of the force. By constructing the FRH with a narrow Gaussian kernel (5 ms), we found that abrupt changes in the force were reflected in abrupt increases in the firing rate caused by transient synchronization. Applying a synchrony threshold to the latter FRH yielded 86% sensitive and 100% specific detection of the stimulus transients. These findings definitively demonstrate that synchrony-division multiplexing occurs in somatosensory cortex. Specifically, we have shown that stimulus intensity (a first-order stimulus feature) is encoded by the rate of asynchronous spiking while abrupt variations in stimulus intensity (a second-order stimulus feature) are simultaneously encoded by the timing of synchronous spikes. The results help reconcile apparently contradictory evidence for rate or temporal coding by showing how these coding strategies can operate together.

**Fig. 1:**
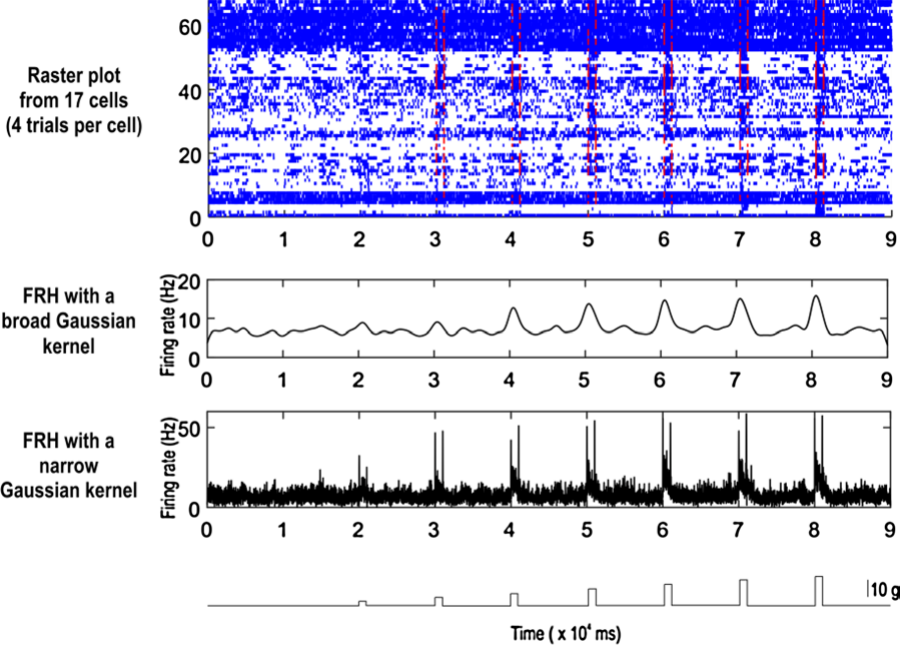
Neural multiplexed coding. Stimulus intensity is encoded by the rate of asynchronous spiking while abrupt variations in stimulus intensity are simultaneously encoded by the timing of synchronous spikes

## P243 The degenerate basis for excitability: Interpreting the pairwise correlation of parameter values in randomly generated model neurons with equivalent excitability

### Arjun Balachandar^1^, Steve Prescott^2^

#### ^1^University of Toronto, Faculty of Medicine, Toronto, Canada; ^2^University of Toronto & The Hospital for Sick Children, Neurosciences and Mental Health & Dept. Physiology, Institute of Biomaterials and Biomedical Eng, Toronto, Canada

##### **Correspondence:** Arjun Balachandar (arjun.balachandar@mail.utoronto.ca)

*BMC Neuroscience* 2018, **19(suppl 2):**P243

Neurons use action potentials, or spikes, to encode information. Proper neural coding thus relies on the proper control of spike initiation. The spike initiation process reflects the highly nonlinear interaction between different ion channels, which means that subtle variations in ion channel expression or function can dramatically impact excitability. Yet excitability is normally very stable, which raises the question of how excitability is regulated so robustly. Emerging data argue that the biophysical basis for excitability is highly degenerate, meaning that many different combinations of ion channel conductances can yield equivalent excitability. This degeneracy is thought to facilitate the robust regulation of excitability by allowing changes in any one ion channel to be compensated for by changes in many other ion channels. We hypothesized that parameters that are able to compensate for one another will be correlated. We further hypothesized that the strength of pairwise correlations will be weakened as the degree of degeneracy (i.e. the number of parameters that can compensate for one another) increases. To test these hypotheses, large sets of conductance-based Morris-Lecar models were generated with randomly chosen parameter values describing different conductance densities or activation properties. A different number of parameters was allowed to vary for each set of models; all other parameters were held constant at their baseline values. From these sets, we identified model neurons with comparable excitability and, using only those models, determined the pairwise correlation between parameters that had been randomly determined. Correlations were observed between some parameters and, as further predicted, the strength of correlation decreased as the number of randomly varied parameters increased. Based on these results, we expect that highly degenerate systems will exhibit only weak pairwise correlations in their parameter values. Conversely, strong correlations may suggest that a system has only modest degeneracy and that it may, therefore, be less able to compensate in the face of strong perturbations.

## P244 Multiplexed coding using differentially synchronized spikes: Part 1, theory and simulations

### Milad Lankarany^1^, Steve Prescott^2^

#### ^1^University of Toronto & The Hospital for Sick Children, Neurosciences & Mental Health, Toronto, Canada; ^2^University of Toronto & The Hospital for Sick Children, Neurosciences and Mental Health & Dept. Physiology, Institute of Biomaterials and Biomedical Eng, Toronto, Canada

##### **Correspondence:** Milad Lankarany (milad.lankarany@gmail.com)

*BMC Neuroscience* 2018, **19(suppl 2):**P244

Multiplexing refers to the simultaneous transmission of multiple signals through a single communication channel. In engineered systems, multiplexing is often implemented by partitioning different signals to different frequency bands (frequency-division multiplexing) or to different temporal epochs (time-division multiplexing). Mounting evidence suggests that the brain also multiplexes but it remains unclear how this might occur. We hypothesized that the brain can form multiplexed representations of first- and second-order stimulus features (i.e. stimulus intensity and abrupt variations therein, such as occur at edges) using spikes that are differentially synchronized across a set of neurons receiving common input. To test our hypothesis, we built a feed-forward neural network comprising Morris-Lecar (ML) model neurons. All neurons received a common mixed input constructed from two distinct signals, slow and fast, plus uncorrelated fast noise. The slow and fast signals represent input from upstream sensory neurons tuned to first- or second-order stimulus features based on their low- or high-pass filter properties, respectively. The two sensory streams converge on the ML model neurons. According to our hypothesis, slow and fast signals are independently encoded by different types of spikes. Specifically, the rate of asynchronous (Async) spikes encode the slow signal whereas the timing of synchronous (Sync) spikes encode the fast signal. To assess the feasibility of the multiplexed coding scheme, we fit linear-nonlinear (LN) rate models to PSTHs from our conductance-based spiking models. In a conventional LN model, input passes through a linear filter and then through a static nonlinearity whose output is firing rate. We constructed a multiplexing LN model with two parallel streams; the same mixed signal is presented to both filters but the output of each filter passes through a different nonlinearity. Unlike the two input streams, which represent input from two differently specialized sets of sensory neurons, the two streams within the LN model represent two operating modes—integration (low-pass filtering) and coincidence detection (high-pass filtering)—used by a set of neurons operating in a hybrid mode. The two-stream LN model more accurately predicted true firing rate (using the PSTH of spiking models as reference) than the one-stream LN model, especially for synchronous spikes. Our results demonstrate that a set of cortical pyramidal cells can implement multiplexing by simultaneously encoding slow and fast features of a mixed signal through a multi-modal filter. These results are further validated experimentally, as presented in our companion poster Multiplexed coding using differentially synchronized spikes: Part 2, experiments.

## P245 An efficient neuron conductance modelling approach using dynamic action potential clamp data

### Yadeesha Deerasooriya^1^, Géza Berecki^2^, David Kaplan^2^, Saman Halgamuge^3^, Steven Petrou^2^

#### ^1^The University of Melbourne, Mechanical Engineering, Melbourne, Australia; ^2^The University of Melbourne, The Florey Institute of Neuroscience and Mental Health, Melbourne, Australia; ^3^The Australian National University, College of Engineering & Computer Science, Canberra, Australia

##### **Correspondence:** Yadeesha Deerasooriya (ydeerasooriy@student.unimelb.edu.au)

*BMC Neuroscience* 2018, **19(suppl 2):**P245

Mathematical modelling significantly contributes to our understanding of the mechanisms underlying neuron and network behaviour. Hodgkin-Huxley (HH) equations are frequently used to model neuron conductances. The majority of the existing HH conductance modelling workflows are based on data acquired under current clamp or voltage clamp (VC) recording conditions. While current clamp provides phenotypically rich information on a neuron’s firing properties, it is often difficult to disentangle the influence of the multiple conductances at play in a real neuron. Isolation of a single conductance by pharmacological or heterologous expression simplifies analysis; however, exhaustive exploration of the kinetic and voltage properties using VC is needed for HH parameterisation that requires weeks of recording time. A more experimental time efficient approach would not only benefit current efforts to develop specific HH models for the entire set of voltage gated channels expressed in the brain, but also free resources to explore these ion channels under pathological or pharmacological conditions. We present an improved HH conductance modelling workflow that uses data derived from dynamic action potential clamp (DAPC) recordings to extract conductance model parameters. A major difference in DAPC over VC recording is that DAPC is an action potential (AP) weighted systems identification approach and is more aligned to the common final use of HH models, which is the building of single neuron and network AP firing models. In this study, first, using fully simulated conditions, we show that with as little as one second of DC recording time, we can produce parameters with an average error of less than 4%. When deployed into simple neuron models, these parameters produced firing rates that approached 100% accuracy in fully simulated experiments. Second, we undertake a real-world test using NaV1.2 channels and show that training our model with five or fewer APs could produce a HH conductance model that predicted the subsequent AP firing with 97% firing rate accuracy. Further, the AP traces overlapped with 94% accuracy. We conclude that DAPC based workflows can be as, or even more, accurate than VC based workflows for extracting HH conductance model parameters. Importantly, this accuracy can be obtained with considerably less recording time and effort providing additional opportunity for exploration of HH conductance equations in different experimental conditions and positioning this approach to have the efficiency to exploit current advances in ion channel genetics and precision drug discovery.

## P246 Unsupervised learning of relative landmark locations using grid cells

### Scott Purdy, Subutai Ahmad

#### Numenta, Redwood City, CA, United States

##### **Correspondence:** Subutai Ahmad (sahmad@numenta.com)

*BMC Neuroscience* 2018, **19(suppl 2):**P246

The ability to rapidly learn new objects and environments is a critical task for the brain and the locations of landmarks is crucial to learning new environments. Grid cells encode local position information through regular, tiled firing fields that are anchored to each learned environment [1]. Grid cells are organized in modules of cells that share the same scale and orientation. Individual modules are ambiguous over larger spaces but sets of grid cell modules with varied scale and orientation accurately and unambiguously encode locations over many large environments [3]. Experimental results show that landmarks and sensory cues are critical for anchoring grid cells and learning new environments, but the exact mechanisms are unclear. It has been proposed that each grid cell module operates like a residue number system [2] and can encode relationships between layers [4]. In this paper we exploit a core property of residue number systems that allows a small, fixed set of connections to perform arithmetic operations. We propose a biologically-plausible model with two grid cell layers that encodes the relative locations of landmarks in environments and show that it reliably encodes environments in the presence of noise. Grid cells in each layer are organized into modules, where each module represents different relative scales and orientations. The “location layer” encodes the location of each sensed landmark. Grid cell modules in the “relative location layer” are paired with a corresponding module in the location layer. Relative location operations are performed only between these paired modules without access to other modules in the layers. The difference (subtraction) between the phase of currently active neurons and the neurons corresponding to landmarks seen in a recent period is encoded in the relative location layer. Locations for recently active landmarks are updated at each step in the location layer with a motor command. However, the relative location layer always represents the relative positions of each landmark pair and is invariant to absolute position within the environment. The model thus learns a set of representations of relative positions of landmarks that is stable for a given environment, and enables efficient disambiguation of previously seen environments (Fig. 1).

**Fig. 1:**
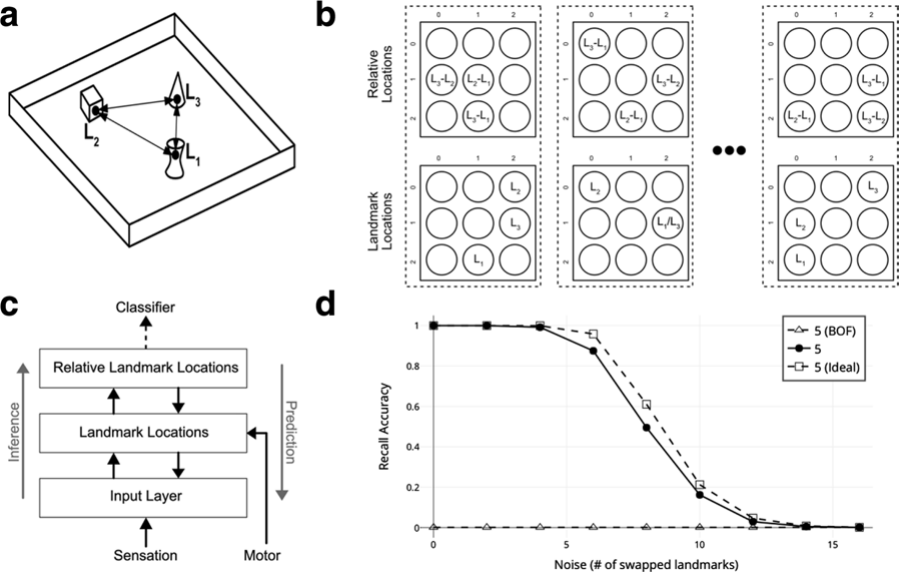
(a) Sample environment with three landmarks. The arrows indicate the relationships encoded by the model after traversing the environment. (b) Illustration of per-module computation of relative locations as the subtraction of pairwise landmark locations after visiting all three locations. (c) A diagram showing the model layers and information flow. (d) Chart of the accuracy of the model on 1000 randomly generated environments as a function of noise (noise is measured as the number of landmarks replaced with a random landmark from the pool). Accuracy is compared against an ideal model and a bag-of-features (BOF) model

Our model is compared with two other models: (1) a bag-of-features model that only compares landmarks without locations and, (2) an ideal model that exhaustively examines all environments to find the best match. Using the relative positions of landmarks, our model is able to achieve perfect accuracy when there is little noise and lags the ideal model slightly for very noisy test cases. The bag-of-features model is no better than chance when a small pool of five landmarks is used. Further research will explore the generalization ability of the model and the addition of an unsupervised temporal clustering layer that can reinstate learned relative location representations in order to predict input sensations that have not recently been seen.

**References**

1. Hafting T, Fyhn M, Molden S, et al. Microstructure of a spatial map in the entorhinal cortex. *Nature,* 2005, 436(7052), 801–806. 10.1038/nature03721
2. Fiete IR, Burak Y, Brookings T. What Grid Cells Convey about Rat Location. *Journal of Neuroscience* 2008, 28(27), 6858–6871. 10.1523/JNEUROSCI.5684-07.2008
3. Sreenivasan S, Fiete I. Grid cells generate an analog error-correcting code for singularly precise neural computation. *Nature Neuroscience* 2011, 14(10), 1330–1337. 10.1038/nn.2901
4. Lewis M, Hawkins J. A neural mechanism for determining allocentric locations of sensed features. *Cosyne Abstracts* 2018, Denver, CO, USA.
5. Hawkins J, Ahmad S, Cui Y. A Theory of How Columns in the Neocortex Enable Learning the Structure of the World. *Frontiers in Neural Circuits* 2017, 11(81), 1–18. 10.3389/fncir.2017.00081

## P247 Development of direction selectivity via a synergistic interaction between short-term and long-term synaptic plasticity

### Nareg Berberian, Matt Ross, Jean-Philippe Thivierge, Sylvain Chartier

#### University of Ottawa, Department of Psychology, Ottawa, Canada

##### **Correspondence:** Nareg Berberian (nareg.berberian@gmail.com)

*BMC Neuroscience* 2018, **19(suppl 2):**P247

Recognizing and tracking the direction of visual objects from the environment is crucial to the control of many animal behaviors. During development, the synaptic circuitry of V1 requires anatomical and functional refinement essential for the emergence of direction selectivity (DS). Under such refinement, synapses strengthen or weaken depending on the relative timing of spikes, a phenomenon known as spike timing-dependent plasticity (STDP). In addition to STDP, a ubiquitous feature in neural circuits is short-term plasticity (STP), where the strength of synapses varies from milliseconds to seconds as a result of recent activity. Early in development, Layer2/3 (L2/3) neurons recorded in V1 of ferrets exhibit spatial clustering, where neighboring neurons exhibit a weak preference for direction [1]. When the network achieves functional maturity, weak direction biases (WDBs) acquired early in development become accurate predictors of strong DS. Despite the crucial role of visual experience needed for proper establishment of DS maps, circuit mechanisms underlying the development of DS remain elusive. In studies of developing neural circuits, STDP has featured prominently in explaining DS through the formation of asymmetric intracortical connections, with weak connections outnumbering strong ones [2]. Amongst infrequent but strong connections, neurons exhibit correlated responses to visual stimuli moving in a particular direction of motion. Given the implication of STDP in explaining DS, what potential mechanism may set the initial condition in driving the onset of WDBs? Using a bio-inspired LIF model, we examine the role of STP as a complementary mechanism to STDP in explaining DS. We simulate a network of excitatory connections with initial weights reflecting the distribution of early L2/3 connectivity. The model captures responses of DS neurons during circuit refinement observed in V1 data (Fig. 1a). While the network receives inputs, the STP mechanism introduces direction-dependent modifications in synaptic strength, which in turn breaks the symmetry of the excitatory connections, introducing an asymmetric connectivity scheme that drives the onset of WDBs. In turn, STDP refines these connections by progressively increasing the asymmetry, making weak connections more prominent, and strong connections a rare commodity, as observed in L2/3 neurons (Fig. 1b). This connectivity scheme reflects preference for direction that matches the WDB introduced by STP. In combining both STP-STDP, and by treating both mechanisms in isolation, we compare versions of the model to analyses of V1 data [3], where increases in DS during subsequent days are primarily due to decreases in spiking responses to the null direction and orthogonal orientation. Learning-related changes in responses are quantified by direction circular variance (Fig. 1c). Overall, results highlight the role of STP as a complementary mechanism to STDP in explaining DS, and propose that STDP is necessary but insufficient to account for the predictive basis of DS.

**Fig. 1:**
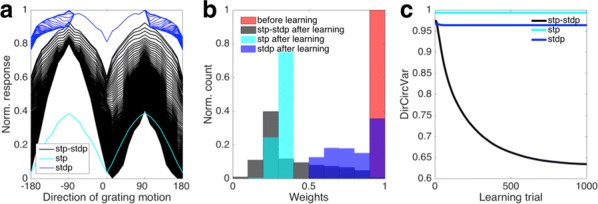
(a). Normalized tuning curve of a single unit in the network mediated by STP-STDP, STP and STDP respectively. Input to the network is comprised of 8 different orientation gratings moving in bidirectional motion along a single axis. (b) Distribution of synaptic weights in response to an orientation grating moving in bidirectional motion along a single axis. (c) Direction circular variance, as a function of learning trial number. Values range from 0 (strong direction selectivity) to 1 (no direction selectivity)

**References**

1. S. D. Van Hooser et al. Initial Neighborhood Biases and the Quality of Motion Stimulation Jointly Influence the Rapid Emergence of Direction Preference in Visual Cortex. *J.Neurosci* 2012
2. L. Cossell et al. Functional organization of excitatory synaptic strength in primary visual cortex. *Nature* 2015
3. J. M. Clemens et al. The Laminar Development of Direction Selectivity in Ferret Visual Cortex. *J. Neurosci.* 2012

## P248 A Bayesian psychophysics model of sense of agency

### Roberto Legaspi^1,2^, Taro Toyoizumi^1,2^

#### ^1^Laboratory for Neural Computation and Adaption, RIKEN Center for Brain Science, Saitama, Japan; ^2^RIKEN CBS-OMRON Collaboration Center

##### **Correspondence:** Roberto Legaspi (toyoizumilab@ml.riken.jp)

*BMC Neuroscience* 2018, **19(suppl 2):**P248

*Sense of agency*, i.e., the feeling that oneself caused something to happen, is fundamental to the experience of volition, self-consciousness and responsibility for one’s own actions, and the degradation of this experience characterizes certain psychiatric disorders. Despite its irrefutable significance, the literature still lacks a mathematical exposition of the computational principles that underlie it. We theorize sense of agency as the*confidence*in one’s perception of action-outcome effect to be consistent with the hypothesis that the self is the*common source*behind this effect. We adapted the Bayesian inference model of Sato, Toyoizumi and Aihara [1] that was originally used to explain the *ventriloquism effect* as a Bayesian estimate of a common source behind the audio-visual stimuli. Formalizing sense of agency by this Bayesian principle distinguishes our theory from existing works. *Intentional binding*, i.e., the perceived shortening of the time interval between voluntary action and its outcome, has been reported as an implicit measure of sense of agency. Yet, the exact nature of this link is far from comprehension [2]. Our Bayesian model gives a simple coherent account of this link: the shorter perceived interval between the action-outcome timings is more consistent with the causal role of one’s action in producing the immediate outcome, and thus increases the confidence of the Bayesian estimate, modeled as sense of agency. We compared the predictions of our model to the results of two pertinent intentional binding studies. The first follows the seminal experiment reported by Haggard, Clark & Kalogeras [3] that showed voluntary actions produced intentional binding effects but involuntary actions produced the prolonged opposite perception of the action-outcome interval. The second case follows the study of Wolpe, Haggard, Siebner & Rowe [4] that investigated the contribution of sensory uncertainty to intentional binding by manipulating the intensity of outcome tones. They showed that when the outcome reliability was reduced, action binding was diminished and tone binding was increased. Our Bayesian psychophysics model reproduces these empirical results based on a computational principle.

**References**

1. Sato Y, Toyoizumi T, Aihara K. Bayesian inference explains perception of unity and ventriloquism after effect: identification of common sources of audiovisual stimuli. *Neural Computation* 2007, 19, 3335–3355
2. Moore JW, Obhi SS. Intentional binding and the sense of agency: A review. *Consciousness & Cognition* 2012, 21(1), 546–561.
3. Haggard P, Clark S, Kalogeras J. Voluntary action and conscious awareness. *Nature Neuroscience* 2002, 5(4), 383–385
4. Wolpe N, Haggard P, Siebner HR, Rowe JB. Cue integration and the perception of action in intentional binding. *Experimental Brain Research* 2013, 229, 467–474.

## P249 On and off responses in auditory cortex may arise from a two-layer network with variable excitatory and inhibitory connections

### Shih-Cheng Chien^1^, Burkhard Maess^1^, Thomas Knoesche^2^

#### ^1^Max Planck Institute for Human Cognitive and Brain Sciences, Leipzig, Germany; ^2^MPI for Human Cognitive and Brain Sciences, Department of Neurophysics, Leipzig, Germany

##### **Correspondence:** Shih-Cheng Chien (vchien@cbs.mpg.de)

*BMC Neuroscience* 2018, **19(suppl 2):**P249

This study investigates dynamical network models designed to reproduce the electrophysiological responses of the human auditory cortex to mismatch negativity eliciting stimulations. To this end, we first focused on modeling On and Off responses of tonal stimuli via recurrent circuits as they exist in the auditory cortex [1–3]. In the simulation, the recurrent circuits are represented by a two-layer network, where the input stimuli from the thalamus reach the 1stlayer and indirectly affect the 2nd-layer activities through recurrent inter-layer connections. With a stream of stimuli fed to the 1stlayer (input), various types of On/Off responses can be reproduced in the 2ndlayer (observation) given proper inter-layer connections. The simulation results account for relevant properties of cortical On/Off responses and provide thereby clues about the underlying physiological mechanisms. (1) A subtle change in inter-layer connections switches the response type between On, On and Off, and Off. Furthermore, it can also switch between ‘sustained’ and ‘suppressed’ activity during the stimulus presentation. Hence, the diverse On/Off responses observed at different locations in auditory cortex [2] may reflect diverse inter-layer connections between the input and the observation layer. Interestingly, symmetric inter-layer connections do not give rise to On/Off responses, underlining the importance of asymmetric forward–backward interactions for the change detection function at the cortical level. (2) The distinct onset- and offset-frequency-receptive-fields (FRF) observed in A1 neurons in [3] can be accounted for by the two-layer scheme. We conclude that the tonotopically organized input layer has distinct equivalent inter-layer connections with the observation layer. (3) Furthermore, the simulation demonstrates that generation of Off response in the 2ndlayer relies on tonic inhibition in the 1stlayer during the stimulus. This nicely matches physiology, as the reduced Off response by NMDA receptor antagonists observed in [1] is due to the reduced inhibition during the stimulus, because excitatory synapses on inhibitory neurons are more sensitive to the NMDA receptor antagonists [4]. To summarize, the recurrent circuits in our model provide a parsimonious solution for the change detection function at the cortical level. This model for On/Off responses will be further used to reproduce mismatch responses to omitted and deviant stimulus in the oddball paradigms.

**References**

1. Hironori B, et al. Auditory cortical field coding long-lasting tonal offsets in mice. *Scientific reports 2016,* 6, 34421.
2. Deneux T, et al. Temporal asymmetries in auditory coding and perception reflect multi-layered nonlinearities. *Nature communications* 2016, 7, 12682.
3. Qin L, et al. Comparison between offset and onset responses of primary auditory cortex ON–OFF neurons in awake cats. *Journal of Neurophysiology* 2007, 97, 5, 3421–3431.
4. Rujescu D, et al. A pharmacological model for psychosis based on N-methyl-D-aspartate receptor hypofunction: molecular, cellular, functional and behavioral abnormalities. *Biological Psychiatry* 2006, 59, 8, 721–729.

## P250 Using GPU enhanced neuronal networks to put real-time brains on board

### James Knight^1^, Alex Cope^2^, Thomas Nowotny^1^

#### ^1^University of Sussex, School of Engineering and Informatics, Brighton, United Kingdom; ^2^University of Sheffield, Sheffield Robotics, Sheffield, United Kingdom

##### **Correspondence:** James Knight (j.c.knight@sussex.ac.uk)

*BMC Neuroscience* 2018, **19(suppl 2):**P250

Various techniques to scale down models of biological neural networks have been developed. However scaling can never preserve all properties of a network, especially not the high number of connections between neurons observed in the brain. Therefore, simulating large-scale biological neural network models remains important and doing so in a reasonable time is one of the major technical challenges in computational neuroscience. Conventionally large-scale simulations are executed on High Performance Computing (HPC) clusters and the tools to distribute neural network simulations across such systems are now relatively mature [1]. However, HPC systems are expensive and not well suited to real-time simulation. Bespoke ‘neuromorphic’ hardware has been developed to address these problems, but they come with their own challenges and limitations.

Graphics Processing Units (GPUs) were initially designed to accelerate the rendering of 3D graphics, but have evolved into massively parallel accelerators, programmable at a relatively high-level of abstraction. This has led to their use in accelerating a wide range of HPC applications and proved vital in the practical application of deep learning. The computational properties of biological neural networks simulations are not necessarily a natural fit for GPU acceleration but modern GPUs can still achieve significant speed-up across a wide range of realistic models. In this work we present the latest developments in GeNN [2], our open source library for generating optimised neural network simulation code for NVIDIA GPUs. GeNN does not require that users understand the details of GPU programming; instead it lets users describe neuron and synapse models using C-like code and then generates the code for running the resultant models on GPUs. To demonstrate the power of this approach we re-implemented a point neuron model of a cortical column [3], which was previously simulated at 0.2× real-time using a 23-node HPC cluster [4]. Using GeNN and a single GPU, we are able to simulate the same model at only 0.25× real-time. This model also demonstrates how GeNN allows the initialisation of a network to be offloaded onto the GPU, significantly reducing overall runtime. GeNN can be used directly from C++, allowing users full control of their simulation and making it easy to integrate GeNN with other libraries. This is particularly useful, now that GPUs such as the Jetson TX1 are available in form factors small enough to use on board autonomous robots. We demonstrate this using a recent model of the bee central complex [5] which we re-implemented in GeNN and used as a controller for a wheeled robot equipped with a Jetson TX1. Finally, for those requiring simulator-agnostic model creation, we have created interfaces to use GeNN from SpineML and the Brian 2 simulator—making it trivial for existing users of these tools to take advantage of GPU acceleration. We demonstrate this with a SpineML model of optical flow calculation inspired by the honeybee [6] (Fig. 1).

**Fig. 1:**
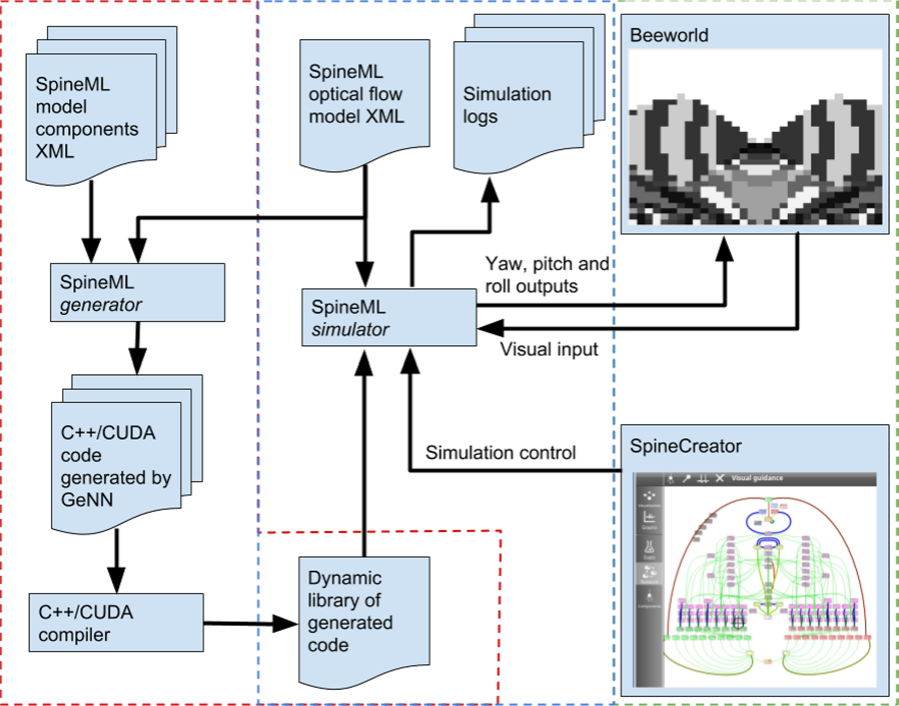
Simulating a honeybee-inspired model of optical flow calculation using SpineML with GeNN providing GPU acceleration. The red dashed border indicates the stages of the process involved in generating GPU code from the SpineML model. The blue dashed border indicates the stages involved in running the resultant simulation. The green dashed border indicates the external simulator-agnostic processes that control and interact with the GPU simulation

**References**

1. Brette R, Rudolph M, Carnevalle T, et al. Simulation of networks of spiking neurons: A review of tools and strategies. *Journal of Computational Neuroscience* 2007, 23(3), 349–398. 10.1007/s10827-007-0038-6
2. Yavuz E, Turner J, Nowotny T. GeNN: a code generation framework for accelerated brain simulations. *Scientific Reports* 2016, 6(18854). 10.1038/srep18854
3. Potjans TC, Diesmann M. The cell-type specific cortical microcircuit: relating structure and activity in a full-scale spiking network model. *Cerebral Cortex* 2012, 24(3), 10.1093/cercor/bhs358
4. Djurfeldt M, Hjorth J, Eppler JM, et al. Run-Time Interoperability Between Neuronal Network Simulators Based on the MUSIC Framework. *Neuroinformatics* 2010, 8(1), 43–60. 10.1007/s12021-010-9064-z
5. Stone T, Webb B, Adden A, et al. An Anatomically Constrained Model for Path Integration in the Bee Brain. *Curr Biol.* 2017, 23, 3069–3085. 10.1016/j.cub.2017.08.052

## P251 Firing probability for a noisy leaky integrate-and-fire neuron receiving an arbitrary external input signal

### Ho Ka Chan, Thomas Nowotny

#### University of Sussex, School of Engineering and Informatics, Brighton, United Kingdom

##### **Correspondence:** Ho Ka Chan (chanhoka911212@yahoo.com.hk)

*BMC Neuroscience* 2018, **19(suppl 2):**P251

In many applications, it is important to understand how quickly and reliable a neuron receiving noisy background input would respond to an external input signal. Here, we consider a leaky integrate-and-fire neuron receiving Gaussian, exponentially correlated noise, and obtain analytically the probability that the neuron fires at least one action potential when an external input signal, in the form of post-synaptic potentials (PSPs), is applied. We derive expressions for the probability of firing in three regimes, when the dynamical time scale of the external input is much quicker than the membrane time constant, when it is much slower, and when it is of comparable speed. In case of fast inputs, the firing probability can be obtained by assuming that the probability distribution of the free membrane potential is translated according to the size of the signal, In the case of slow inputs, we can use the adiabatic approximation, assuming that the membrane potential distribution is quasi-stationary, and obtain the firing probability using previously developed methodologies [1, 2]. In the most challenging case of comparable timescales of membrane potential and input fluctuation, we derive a novel correction to the adiabatic case by applying a Taylor expansion on the noise-induced component of the membrane potential. To determine the spiking probability for arbitrary input waveforms, we heuristically combine the three approximations depending on the time derivative of the input. Our analytic approximations match well with the results obtained from numerical simulations for input signals of a wide range of sizes and shapes, and under both strong and weak background noise (See Fig. 1). Our method can be used to estimate the response latency of sensory neurons and to study the reliability of neural responses to temporally filtered and jittered synchronous input.

**Fig. 1:**
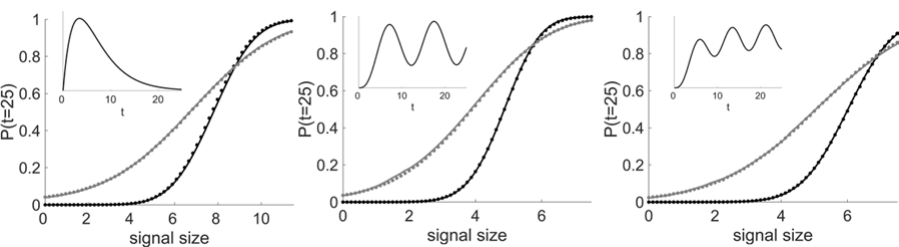
The approximation of the probability of LIF neuron firing within 25 ms after the onset of an external signal, which shape is shown in the insets. The Dots correspond to the result of numerical simulations and the solid lines are the results obtained from our analytical approximation. Black (gray) dots and lines corresponds to weak (strong) background noise. The approximation remains excellent even with signals of complex shapes for both strong and weak background noise

**References**

1. Fourcaud N, Brunel N. Dynamics of the Firing Probability of Noisy Integrate-and-Fire Neurons. *Neural Comput*. 2002, 14, 2057–2110
2. Moreno-Bote R, Parga N. Response of integrate-and-fire neurons to noisy inputs filtered by synapses with arbitrary timescales: firing rate and correlations. *Neural Comput*. 2010, 22(6), 1528–152.

## P252 Computing reward prediction errors and learning valence in the insect mushroom body

### James Bennett, Thomas Nowotny

#### University of Sussex, School of Engineering and Informatics, Brighton, United Kingdom

##### **Correspondence:** James Bennett (james.bennett@sussex.ac.uk)

*BMC Neuroscience* 2018, **19(suppl 2):**P252

Decision making in the insect brain utilizes learned valence to bias particular actions in response to the animal’s environment. A key site for learning in insects is the mushroom body (MB) [1], where environmental cues are encoded by Kenyon cells (KCs) and assigned valence by MB output neurons (MBONs). Valence memories are learned via reward modulated synaptic plasticity and stored in KC-MBON synapses, at which rewards are signaled by dopaminergic neurons (DANs). Recent studies in *Drosophila* have revealed intricate connections between these three cell types, which are necessary for learning appropriate actions [2, 3]. Here, we present a MB model that captures these data to compute reward prediction errors (RPEs) for learning, thus implementing the Rescorla–Wagner model. Current models posit that the *alpha*− (A) and *beta*− (B) lobes of the MB encode the signed valence of reward information and actions [1]: DANs in the A-lobe (D− in Fig. 1a) are excited by negative (−ve) rewards, and depress active KC synapses onto MBONs that bias actions toward approach (M+ in Fig. 1a); DANs in the B-lobe (D+ in Fig. 1a) are excited by positive (+ve) rewards, depressing active KC synapses onto MBONs that bias actions toward retreat (M− in Fig. 1a). If MBONs provide excitatory feedback to their respective DANs, the learned reduction in MBON firing can offset the excitatory reward signal arriving at that DAN. Thus, D+ and D− may both encode RPEs in the signed (+ve or –ve) reward valence. Moreover, the difference in MBON firing rates, *m*+-*m*−, signals the learned net valence associated with a sensory cue.

**Fig. 1:**
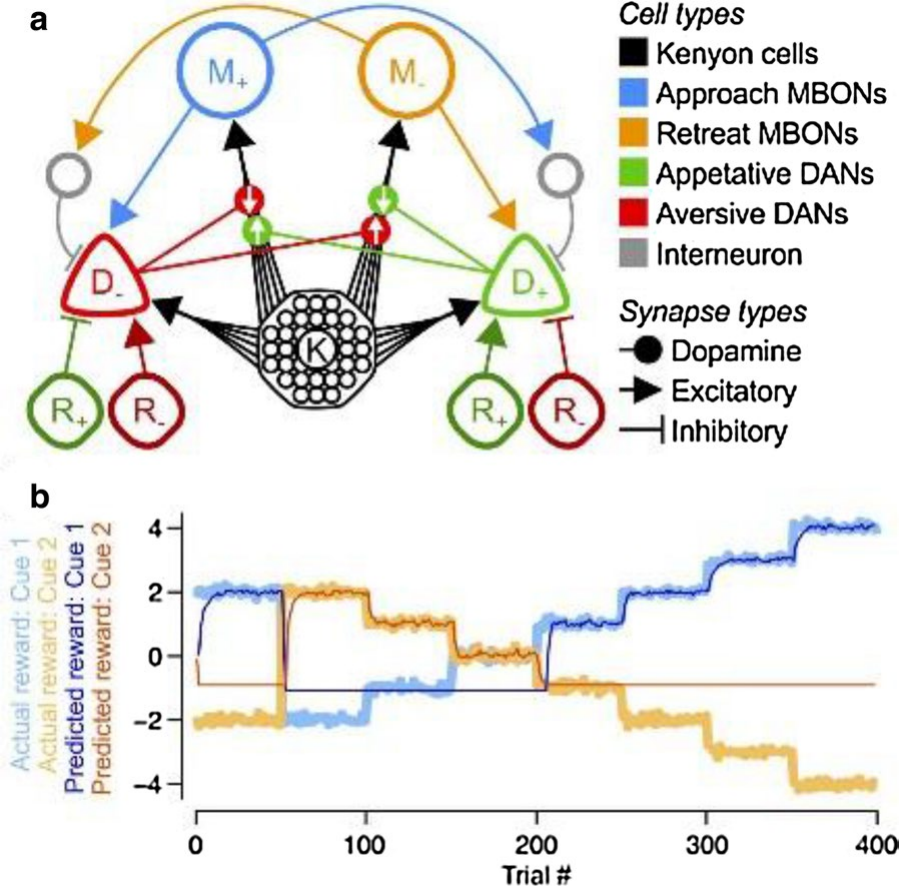
(a) Schematic of the Signed Reward Prediction Error Circuit. White arrows on the dopamine synapses indicate the relative change in synaptic weight with increases in dopamine released by the respective DAN. (b) Reward contingencies, and reward predictions computed from the MBON firing rates, in a two-alternative forced choice task. Reward predictions track the actual rewards associated with each option, and become highly erroneous for options that are repeatedly not chosen

We first show two problems with this model: (1) it cannot learn reward magnitudes above an upper bound; (2) it learns only when KC-DAN excitation is minimal or absent, in contrast with experiments [2]. We propose a solution, in which D+/D− neurons are instead inhibited by –ve/+ ve reward signals, and in which KC-DAN excitation is required (Fig. 1a). We also derive a plasticity rule for KC-MBON synapses that performs gradient descent on the RPE, and that resembles experimentally observed rules [4]. We call this model the Signed Valence Circuit (SVC). As before, DANs encode RPEs in the signed reward valence (Fig. 1d), and the difference in DAN firing rates, *d*+-*d*−, yields the net RPE (Fig. 1e). The SVC can learn rapid changes to reward contingencies in just 5–10 trials (Fig. 1c). In the SVC, D+/D− respectively signal RPEs for –ve/+ ve rewards, so do not actually contribute to learning +ve/−ve valences, counter to experimental evidence [1]. However, in a dual version of this circuit—in which D+/D− are driven by +ve/–ve rewards—D+ no longer signals decrements in –ve rewards, again in contrast with experiments [5]. We therefore combine the SVC and its dual to produce the Signed RPE Circuit (SRC; Fig. 1b), in which the lobes encode the signed RPE of both +ve and –ve reward signals (Fig. 1G). Lastly, the SRC performs well in a traplining task (Fig. 1H–I)—repeating learned routes and minimizing the distance traveled between feeding areas—a behavior exhibited by bees [6] and other species, and a foraging analogue of the travelling salesman problem.

**References**

1. Owald,Waddell. *Curr. Op. Neurobiol*. 2015, 35, 178–184
2. Cervantes-Sandoval, et al. eLife 2017, 6, e23789
3. Felsenberg et al. *Nature* 2015, 544, 240–244
4. Hige et al. *Neuron* 2015, 88, 985–998
5. Perisse et al. *Neuron* 2015, 79, 945–956
6. Lihoreau et al. *Biol. Lett*. 2012, 8, 13–16

## P253 Characterization of short-term synaptic plasticity in mouse primary visual cortex

### Jung Lee, Stefan Mihalas, Luke Campagnola, Stephanie Seeman, Pasha Davoudian, Alex Hoggarth, Tim Jarsky

#### Allen Institute for Brain Science, Modelling, Analysis and Theory, Seattle, WA, United States

##### **Correspondence:** Jung Lee (jungl@alleninstitute.org)

*BMC Neuroscience* 2018, **19(suppl 2):**P253

Synapses do not merely relay spikes from presynaptic to postsynaptic neurons. Instead, they edit outputs of presynaptic neurons depending on presynaptic spike history. During stimulation period, synaptic transmission can facilitate or depress [1–3]. However, the exact functions of this ‘short-term synaptic plasticity’ remains contentious partly due to its diversity. Thus, it is necessary to characterize short-term synaptic plasticity to comprehend its functions. To this end, we analyzed the data from Allen Institute’s large scale multi-patch pipeline project, which focuses on studying synaptic connections in mouse primary visual cortex (V1). As short-term synaptic plasticity depends on both pre- and post-synaptic neuron classes [4–6], we defined the synapse classes using pre- and post-synaptic neuron classes and studied their properties. To characterize short-term synaptic plasticity in synapse classes, we developed descriptive models. Specifically, our models considered five temporal dynamics of synaptic transmission: depression (Eq. 1 in Fig. 1a), facilitation (Eq. 2 in Fig. 1a), use-dependent modulation (Eq. 3 in Fig. 1a), desensitization of postsynaptic receptors (Eq. 4 in Fig. 1a) and slow modulation of release probability (Eq. 5 in Fig. 1a). The amplitude of postsynaptic potentials (PSPs) is proportional to resource (*n*), utility (*P*) and sensitivity of receptor (*S*); see Eq. 6 in Fig. 1a. Depression and facilitation were modeled by the dynamical systems proposed by the two groups, and the rest were modeled by those presented in an earlier work [7].

**Fig. 1:**
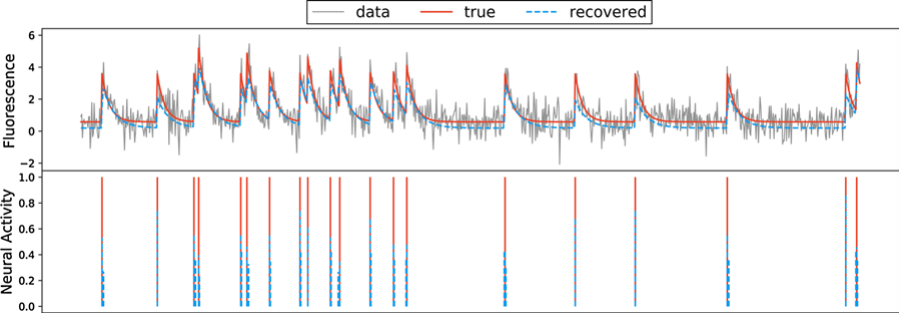
The top panel gives the simulated, true, and computed fluorescence traces. The bottom panel shows the true and computed spikes. We recover all spikes, with few false positives

In the experiments, PSPs were measured while presynaptic neurons were stimulated with spike trains at 10, 20, 50, 100 and 200 Hz, and we fitted the time courses of PSPs to our synapse models. Specifically, we first used *x*-means clustering to identify homogenous synapses in the class. Then, we fitted the average PSPs from homogeneous synapses to the model using ‘LMFIT’, an open-source package developed for flexible non-linear least-square minimization [8]. So far, we constructed ~ 10 synapse models in V1 that can capture short-term synaptic plasticity observed in V1 (See Fig. 1 for an example). These models suggest 1) that most synapse classes depress in V1 and 2) that short-term synaptic plasticity depend mainly on presynaptic neurons. We believe that these synapse models would allow us to better understand the neural basis of visual perception. The following points should be underscored. First, our synapse models will be further refined, as more data become available. Second, we are currently building network models of V1 with our synapse models to study functions of short-term synaptic plasticity in V1 in visual perception. For instance, we are studying short-term synaptic plasticity’s contribution to the stimulus-specific adaptation in V1.

**References**

1. Abbots LF, Verela JA, Sen K, Nelson SB. Synaptic depression and cortical gain control. *Science* 1997, 275, 220–224.
2. Markram H, Wang Y, Tsodyks M. Differential signaling via the same axon of neocortical pyramidal neurons. *PNAS* 1998, 95(9), 5323–5328.
3. Stevens CF, Wang Y. Changes in reliability of synaptic function as a mechanism for plasticity. *Nature* 1994.
4. Beierlein M, Gibson KR, Connors BW. Two dynamically distinct inhibitory networks in layer 4 of the neocortex. *Journal of Neurophysiology* 2003, 90(5), 2987–3000.
5. Gibson JR, Beierlein M, Connors BW. Two networks of electrically coupled inhibitory neurons in neocortex. *Nature* 1999, 402(6757), 75–79.
6. Pala A, Petersen CCH. In vivo measurement of cell-type-specific synaptic connectivity and synaptic transmission in layer 2/3 mouse barrel cortex. *Neuron* 2015, 68–75.
7. Hennig C, Liao TF. How to find an appropriate clustering for mixed‐type variables with application to socio‐economic stratification. *Royal Statistics Society* 2013.
8. Newville M, Stensitzki T, Allen DB, et al. LMFIT: Non-Linear Least-Square Minimization and Curve-Fitting for Python.

## P254 Spatial patterns of synchrony from electrical synapses in the inferior olive

### Thomas Chartrand^1^, Mark Goldman^2^, Timothy Lewis^3^

#### ^1^University of California, Davis, Applied Mathematics and Center for Neuroscience, Davis, CA, United States; ^2^University of California, Davis, Departments of Neurobiology, Physiology and Behavior & Ophthalmology and Vision Science, Davis, CA, United States; ^3^University of California, Davis, Department of Mathematics, Davis, CA, United States

##### **Correspondence:** Thomas Chartrand (tmchartrand@ucdavis.edu)

*BMC Neuroscience* 2018, **19(suppl 2):**P254

The inferior olive is thought to contribute to the generation of timing and error signals for motor control. However, the specific role of its distinctive spatiotemporal activity patterns is still controversial. Synchronous states have been observed both in olivary subthreshold oscillations in vitro and in complex spikes of cerebellar Purkinje cells in vivo, which serve essentially as a one-to-one readout of spiking in olivary climbing fiber output. These observations show two types of spatial structure: zero-lag correlations that decay with distance (localized synchrony), and synchrony at nonzero phase lags that increase with distance (phase waves). Although evidence suggests that electrical synapses between olivary cells underlie this synchronization, the details of spatial pattern formation remain largely unexplained. We present a model that explains how both types of synchronized states arise from the interplay of subthreshold membrane potential oscillations and spatially constrained electrical coupling. Although the olive is known to have the highest density of electrical coupling (via dendro-dendritic gap junctions) of any brain region, olivary cells also exhibit a high level of heterogeneity in the intrinsic oscillation frequency, which opposes the synchronizing effect of the coupling. We capture these features in a network model of phase oscillators, derived from conductance-based models of olivary neurons following the theory of weakly coupled oscillators (Fig. 1). We show that a network of phase oscillators with the appropriate balance of heterogeneity and local coupling will produce spatial phase gradients which are stable and robust to perturbations of the network. This may be a generic mechanism for brain to harness random heterogeneity to create stable patterns of timing. We next consider the patterns of spiking triggered by external input to a network in the subthreshold phase gradient state. Depending on the spatial structure of input and the degree to which spike output depends on phase of oscillation, external input can evoke different types of patterns, reproducing both zero-lag and spatial-lag observations of complex spike synchrony. We present examples of both states in a spiking network model of olivary neurons, and discuss the potential functional role of these structured spiking states in terms of generating motor timing and filtering error signals.

**Fig. 1:**
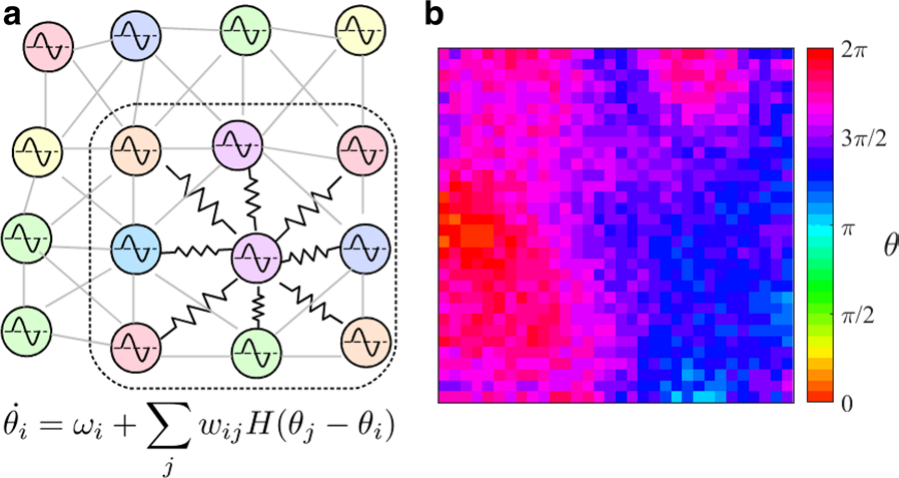
(a) A schematic diagram of a network of phase oscillators with local electrical coupling and random intrinsic frequencies. (b) Synchronization of locally-coupled phase oscillator network in phase gradient state

## P255 Dopaminergic changes in striatal pathway competition modify specific decision parameters

### Jonathan Rubin^1^, Kyle Dunovan^2^, Catalina Vich^3^, Matthew Clapp^4^, Timothy Verstynen^2^

#### ^1^University of Pittsburgh, Department of Mathemathics, Pittsburgh, PA, United States; ^2^Carnegie Mellon University, PA, United States; ^3^Universitat de les Illes Balears, Spain; ^4^University of South Carolina, SC, United States

##### **Correspondence:** Jonathan Rubin (jonrubin@pitt.edu)

*BMC Neuroscience* 2018, **19(suppl 2):**P255

Mammals selecting actions in noisy contexts quickly adapt to unexpected outcomes to better resolve uncertainty in future decisions. Such feedback-based changes in behavior rely on plasticity within cortico-basal-ganglia-thalamic (CBGT) networks, driven by dopaminergic (DA) modulation of cortical inputs to the direct (d) and indirect (I) pathways of the striatum. DA error signals favor the D pathway over the I pathway for rewarding actions with the opposite tendency for aversive ones, effectively encoding the values of alternative actions. It remains unclear how changes in action value influence the mechanisms of the action selection process itself. Here we use a biologically plausible spiking model of CBGT networks to illustrate (1) how feedback-driven DA signals modify the strength of D and I pathways in accordance with a simple reinforcement learning model and (2) how asymmetries in D/I efficacy, resulting from the learning process, impact the accumulation of evidence for alternative actions. Simulations of corticostriatal synapses showed that DA feedback leads to asymmetrical weights in the D and I pathways within a given action channel and the ratio of these weights (w_D/w_I) effectively encodes the action’s expected value (Q). We then simulated the full CBGT network in the context of a simple 2-choice value-based decision task under different weighting schemes for cortical inputs to the D and I pathways (high, medium, and low w_D/w_I) for one of the action channels. The simulated response times from these simulations were fit with two variants of a drift–diffusion model (DDM), leaving either the drift-rate or the boundary height free to vary with the w_D/w_I ratio. As w_D/w_I increases, the speed of information accumulation in the decision process also increases, providing a direct mapping between network level properties of CBGT systems and cognitive decision processes. Finally, we have incorporated the corticostriatal plasticity module into the CBGT network model to form an integrated learning and decision-making network. Fits of the DDM to integrated network outputs will provide novel predictions about the mapping between CBGT and DDM parameters—drift-rate, boundary height, accumulation onset time, bias, and others—that best captures RTs associated with variable reward schedules in human experiments performed in our lab. This framework also allows us to explore how particular basal ganglia network features, such as tonic dopamine levels and changes in synaptic connection strengths, relate to changes in decision-making strategies, including those driven by behavioral parameters such as expectation and motivation.

## P256 The mean-field theory of dynamically balanced neuronal networks

### Takashi Hayakawa, Tomoki Fukai

#### RIKEN Brain Science Institute, Laboratory for Neural Coding and Brain Computing, Wako, Japan

##### **Correspondence:** Takashi Hayakawa (hayakawa.takashi@nihon-u.ac.jp)

*BMC Neuroscience* 2018, **19(suppl 2):**P256

Coherence among activities of individual neurons and local field potential oscillations has been suggested as a clue to the mechanisms underlying information integration in the brain [1–6]. Experiments have also revealed that balanced excitatory and inhibitory synaptic inputs to neurons underlie the local-field potential oscillations [7]. However, despite recent pioneering studies of oscillations in neuronal networks [8–15], how the local field potential oscillations emerge as a result of balanced excitatory and inhibitory inputs and how individual neuronal activities become coherent with those oscillations remain to be understood theoretically. In the present study, we investigate a simple neuronal-network model on a dynamical balance between excitatory and inhibitory recurrent inputs, developing an analytical method that extends a previous theory [16] and describes this type of networks theoretically for the first time see [17] for a preprint,. In this network, microscopic dynamics of a small number of neurons are amplified by the strong excitation and inhibition and reflected in the macroscopic dynamics of the mean synaptic input over the network which have been considered as the origin of local field potentials. Conversely, the macroscopic dynamics of the mean synaptic input constrain the microscopic fluctuations in the activities of individual neurons. As a result of these bidirectional interscale interactions, oscillatory patterns of the mean synaptic input similar to local field potential oscillations spontaneously emerge. As the magnitude of balanced excitation and inhibition is increased, the mean synaptic input and the neuronal activities become coherent. This type of coherent states can also be induced by applying external stimuli to a small number of neurons in the network. The above behaviour of the network model is predicted by our theory with good quantitative agreement between the theory and direct simulations. Numerical results further suggest that the coherent states allow selective and reproducible read-out of information from the network. In conclusion, our results suggest a novel form of neuronal information processing that accounts for the emergence of local field potential oscillations, their coherence with neuronal activities, and the role of coherent dynamics in information processing in the brain. We also expect our results to provide a foundation for designing artificial neuronal networks for reservoir computing and beyond.

**References**

1. O’Keefe J, Recce ML. Phase relationship between hippocampal place units and the EEG theta rhythm *Hippocampus* 1993, 3, 317.
2. Buzsáki G, Theta oscillations in the hippocampus. *Neuron* 2002, 33, 325.
3. Harris KD, et al. Spike train dynamics predicts theta-related phase precession in hippocampal pyramidal cells. *Nature* 2002, 417, 738.
4. Fries P, et al. The gamma cycle. *Trend. Neurosci*. 2007, 30, 309.
5. Poulet JF, Petersen CC, Internal brain state regulates membrane potential synchrony in barrel cortex of behaving mice. *Nature* 2008, 454, 881.
6. Strüber D, et al. Antiphasic 40 Hz oscillatory current stimulation affects bistable motion perception. *Brain Topography* 2014, 27, 158.
7. Atallah, Scanziani M, Instantaneous modulation of gamma oscillation frequency by balancing excitation with inhibition. *Neuron* 2009, 62, 566.
8. Faugeras O, et al. A constructive mean-field analysis of multi-population neural networks with random synaptic weights and stochastic inputs. *Front. Comp. Neurosci*. 2008, 3, 1.
9. Hermann G, Touboul J. Heterogeneous connections induce oscillations in large-scale networks. *Phys. Rev. Lett*. 2012, 109, 018702.
10. Cabana T, Touboul J. Large deviation, dynamics and phase transitions in large stochastic and disordered neural networks. *J. Stat. Phys*. 2013, 153, 211.
11. Lagzi F, Rotter S. A Markov model for the temporal dynamics of balanced random networks of finite size. *Front. Comp. Neurosci*. 2014, 8, 1.
12. Montbrió E, et al. Macroscopic description for networks of spiking neurons. *Phys. Rev. X* 2015, 5, 021028.
13. Sancristóbal B, et al. Collective stochastic coherence in recurrent neuronal networks. *Nat. Phys*. 2016, 12, 881.
14. García del Molino LC, et al. Synchronization in random balanced networks. *Phys. Rev. E* 2013, 88, 042824.
15. Stern M, Abbott L. Dynamics of rate-model networks with seperate excitatory and inhibitory populations. SFN2016.
16. Kadmon J, Sompolinsky H. Transition to chaos in random neuronal networks. *Phys. Rev. X* 2015, 5, 041030.
17. Hayakawa T, Fukai T. Spontaneous and stimulus-induced coherent states in dynamically balanced neuronal networks. arXiv:1711.09621

## P257 Estimation of model parameters from LFPs of spiking neuron networks using deep learning

### Espen Hagen^1^, Gaute Einevoll^2^, Jan-Eirik W Skaar^2^, Alexander J Stasik^1^, Torbjørn V Ness^2^

#### ^1^University of Oslo, Department of Physics, Oslo, Norway; ^2^Norwegian University of Life Sciences, Faculty of Science and Technology, Ås, Norway

##### **Correspondence:** Espen Hagen (espen.hagen@fys.uio.no)

*BMC Neuroscience* 2018, **19(suppl 2):**P257

Biologically inspired machine learning (“deep learning”) techniques such as convolutional neural networks (CNNs) have shown tremendous power to detect non-trivial features in a wide repertoire of data types. Increased computational power, availability of large labeled data sets, and general purpose and open source software implementations such as Google’s Tensorflow (https://www.tensorflow.org) ensure that the popularity of these techniques is rapidly rising, for example in various image classification tasks [1]. In experimental neurosciences, data with high-dimensional features is routinely collected using a variety of techniques. One such comparably easy-to-perform technique is measurements of extracellular potentials by insertion of electric probes into neural tissue. However, the interpretation of the low frequency part of the signal, the local field potential (LFP), is hard because the measured signals result from both local and remote neural activity. Applications of CNNs for LFP analysis are not yet widespread, in particular with regards to detecting and classifying neural events or states that may not readily be detected using conventional methods. Here, we ask the question: Can CNNs be trained to estimate the underlying model parameters of spiking neuron networks from the LFPs they generate? We apply a recently developed hybrid scheme for computing extracellular potentials from spiking point-neuron network models [2] to a cortex-like, sparsely connected network model consisting of one excitatory and one inhibitory population of leaky integrate-and-fire (LIF) neurons. The network is simple enough to allow for detailed analysis of its state space [3]. We systematically vary different network parameters (for example connection strengths and amount of external input), run each simulation and compute the corresponding ‘virtual’ LFP signals as if measured at different depths through the neuronal populations. We then train CNNs set up using Tensorflow on subsets of LFP data, and explore to what extent model parameters can be estimated by the CNNs for the remaining LFP data. We indeed find that these CNNs can, based on the generated LFP, accurately identify the model parameters underlying the simulations by this relatively simple spiking network. This work contributes to a better understanding of what information is available in the LFP signal. It is also a first step in the direction of new analysis methods applicable to experimental LFP data that can be used to obtain more detailed information about the underlying neurons and neural networks.

**References**

1. Rawat W, Wang Z. Deep Convolutional Neural Networks for Image Classification: A Comprehensive Review. *Neural Comput* 2017, 29, 2352–2449
2. Hagen E, Dahmen D, Stavrinou ML, et al. Hybrid Scheme for Modeling Local Field Potentials from Point-Neuron Networks. *Cereb Cortex* 2016, 26, 4461–4496
3. Brunel N. Dynamics of Sparsely Connected Networks of Excitatory and Inhibitory Spiking Neurons. *J Comput Neurosci* 2000, 8, 183–208

## P258 Electrical synapses between inhibitory neurons shape the responses of principal neurons to transient inputs in the thalamus: a modeling study

### Julie Haas, Tuan Pham

#### Lehigh University, Dept. of Biological Sciences, Bethlehem, PA, United States

##### **Correspondence:** Julie Haas (julie.haas@gmail.com)

*BMC Neuroscience* 2018, **19(suppl 2):**P258

As multimodal sensory information proceeds to the cortex, it is intercepted and processed by the nuclei of the thalamus. The main source of inhibition within thalamus is the reticular nucleus (TRN), which collects signals both from thalamocortical relay neurons and from thalamocortical feedback. Within the reticular nucleus, neurons are densely interconnected by connexin36-based gap junctions, known as electrical synapses. Electrical synapses have been shown to coordinate neuronal rhythms, including thalamocortical spindle rhythms, but their role in shaping or modulating transient activity as is propagates through the brain is far less understood. Our recent findings on the plasticity of electrical synapses led us to investigate the impact of changes in electrical synapse strength on the circuit that embeds them. We constructed a four-cell single-compartment Hodgkin-Huxley model comprising thalamic relay and TRN neurons, and used it to investigate the impact of electrical synapses on closely timed inputs delivered to thalamic relay cells (Fig. 1). We showed that the electrical synapses of the TRN assist in cortical discrimination of these inputs through effects of truncation, delay or inhibition of thalamic spike trains. Electrical synapses lead to increased thalamocortical spiking separation and independence when inputs to the thalamus are dissimilar in strength and arrival timing. Conversely, electrical synapses can result in fusion of thalamocortical spiking by masking smaller strength and/or temporal differences between the incoming synaptic inputs. Thus, electrical synapses within the thalamocortical circuit strongly influence what the cortex receives from thalamus, and whether the information relayed to cortex is amenable to discrimination. We expect that these are principles whereby electrical synapses play similar roles in regulating the processing of transient activity in excitatory neurons across the brain. Our simulations provide specific predictions regarding the impact of electrical synapses and plasticity in thalamocortical processing, which we are currently testing in experiments in vitro.

**Fig. 1:**
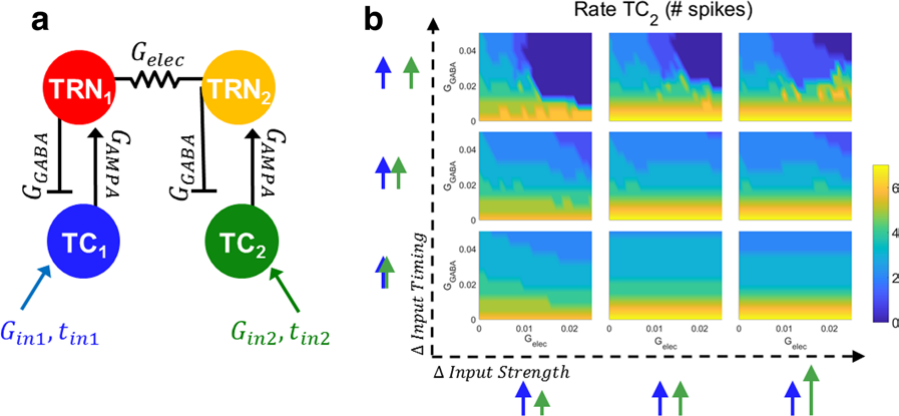
(a) Thalamocortical circuit model that we used to investigate the impact of TRN electrical synapses on TC spiking. (b) Spiking in TC2 was most strongly modified in rate for inputs that were different. Electrical and inhibitory synapses acted synergistically to control TC spiking

## P259 An auto-encoder architecture for transcriptomic cell type analysis: 2d mapping of mouse cortical cells

### Uygar Sumbul

#### Allen Institute for Brain Science, Modelling, Analysis and Theory, Seattle, WA, United States

##### **Correspondence:** Uygar Sumbul (uygars@alleninstitute.org)

*BMC Neuroscience* 2018, **19(suppl 2):**P259

Single cell RNA sequencing (scRNA-seq) can obtain snapshots of the transcriptomic identities of single cells, including neurons. While it has emerged as a high-throughput method of generating cell atlases based on similarities in gene expression profiles, its high-dimensional representations and complicated noise processes create dimensionality reduction challenges for many problems. Here, we present an auto-encoder architecture that improves the quality of the low-dimensional embeddings of scRNA-seq data. We show that the resulting embedding can identify cortical cell types and resolve previously merged classes in a recent deep scRNA-seq dataset of more than 20,000 cells.

## P260 Characterizing spatial attributes of structural networks in acute traumatic brain injury

### Margaret Mahan^1^, Shivani Venkatesh^2^, Maxwell Thorpe^2^, Tessneem Abdallah^2^, Hannah Casey^2^, Aliya Ahmadi^2^, Mark Oswood^3^, Charles Truwit^3^, Chad Richardson^4^, Uzma Samadani^2^

#### ^1^University of Minnesota, Biomedical Informatics and Computational Biology, Minneapolis, MN, United States; ^2^Hennepin County Medical Center, Neurosurgery, Minneapolis, MN, United States; ^3^Hennepin County Medical Center, Radiology, Minneapolis, MN, United States; ^4^Hennepin County Medical Center, General Surgery, Minneapolis, MN, United States

##### **Correspondence:** Margaret Mahan (mahan027@umn.edu)

*BMC Neuroscience* 2018, **19(suppl 2):**P260

**Introduction:** Traumatic brain injury (TBI) occurs when an external force results in structural damage to the brain, typically in white matter regions. In cases of mild to moderate TBI, this damage often goes undetected with conventional imaging techniques. However, since the structural damage invokes axon shearing, magnetic resonance imaging (MRI) with the application of network science, may improve detection and ultimately uncover the underlying dysfunction in TBI. Furthermore, research using Diffusion Tensor Imaging (DTI) has shown that diffusion properties, as well as connectivity patterns, can depict properties of TBI networks, namely, decreased fractional anisotropy (FA), increased mean diffusivity (MD), higher small-worldness, higher modularity, and lower global efficiency. While these metrics provide insights into the properties of the structural network, the specific attributes of the network that are disrupted after TBI are still unknown. Here, we aim to further advance the knowledge about the spatial attributes of TBI-related network dysfunction by applying novel network science methods.

**Methods:** The study enrolled 29 controls and 43 TBI patients who underwent an MRI scan (sagittal T1-weighted volumes and axial diffusion-weighted volumes acquired in 32 directions) within 4 ± 2 days from injury. The Human Connectome Project Multimodal Parcellation, providing 180 regions per hemisphere, was utilized for node definitions in the structural graph with each region corresponding one node. Further resolution of these graphs was achieved at 2-fold, 5-fold, and 10-fold splitting, via k-means with biological constraints, of each region. Edges in the structural graph were represented by streamlines seeded from each white matter voxel in the cerebrum, thresholded by anisotropy and curvature, and calculated using probabilistic Bayesian tractography and deterministic FACT algorithm tractography. Streamlines were retained if two different nodes were connected, the connection included the seed, and was at least 10 mm. The resulting edge definitions for weighting the structural graphs include: streamline counts, mean FA, along with corrections for node volume and streamline length. Adjacency matrices were constructed using the aforementioned node and edge definitions. These matrices were analyzed for graph measures of segregation, integration, and influence with subsequent group analysis via participation coefficient. The final analysis applied spatial machine learning algorithms for assessing network dysfunction.

**Results:** Previous research has shown sensitivity in results from structural network construction. Here, we comprehensively construct a variety of graphs for each subject and utilize each graph as a valid representation of the structural network. First, the diffusion properties in TBI subjects showed similar patterns for alterations in FA and MD, and specific track related decreases in FA will be presented. Second, graph metrics for segregation, integration, and influence show interesting changes in the acute TBI case, most notably were changes in network efficiency. Next, feature extraction was implemented to find indications of disconnections in the TBI structural network followed by spatial machine learning algorithms to show spatial attributes of these network difference. The results present a novel step towards understanding the structural network dysfunction in acute mild to moderate TBI.

## P261 Mass-action vs stochastic simulations of Ca2+ dependent vesicle release latency

### Victor Matveev

#### New Jersey Institute of Technology, Department of Mathematical Sciences, Newark, NJ, United States

##### **Correspondence:** Victor Matveev (matveev@njit.edu)

*BMC Neuroscience* 2018, **19(suppl 2):**P261

A distinguishing feature of synaptic and endocrine secretory vesicle exocytosis is the high degree of variability in response latency. Stochastic Ca2+ channel gating is a major source of this stochasticity [2–4]. Another reason for high variability is that only a small number of Ca2+ ions enter the cell through a single channel during an action potential, as well as the stochasticity in the Ca2+ binding to Ca2+ buffers and sensors. This leads to a widely-held assumption that solving mass-action reaction–diffusion equations for buffered Ca2+ diffusion does not provide sufficient accuracy for modeling Ca2+-dependent cell processes. However, several recent comparative studies show a surprising close agreement between deterministic and trial-averaged stochastic simulations of Ca2+ diffusion, buffering and binding [1, 2], as long as Ca2+ channel gating is not strongly Ca2+ dependent [2–4]. We present further analysis and comparison of stochastic and mass-action simulations, focusing on Ca2+ dynamics downstream of Ca2+ channel gating. Smoldyn (www.smoldyn.org) is used for stochastic simulations, while CalC (Calcium Calculator) is used for deterministic simulation (www.calciumcalculator.org), with Ca2+-binding scheme of Felmy et al. [5]. We show that the discrepancy between deterministic and stochastic approaches can be surprisingly small even when only as few as 30 ions enter per single channel-vesicle complex, assuming that Ca2+ binding depletion is accurately implemented, as described previously [1, 2]. We argue that the reason for the close agreement between stochastic and mass-action simulations is that the discrepancy between the two approaches is determined by the size of correlations between reactant molecule numbers rather than their variance. Further, contrary to naïve intuition, mass-action reaction–diffusion description provides an estimate of the vesicle release latency (i.e. first passage time) probability density function, since it is identical to the system of equations for the first moments of the Ca2+-sensor system derived from the underlying master equations under the moment-closing assumption of negligible correlations (Fig. 1). This may explain the close match between stochastic and mass-action simulations of Ca2+ dependent exocytosis, despite the high variability in Ca2+ diffusion, buffering and binding. Potential explanations for the small size of molecule number covariances will be discussed.

**Fig. 1:**
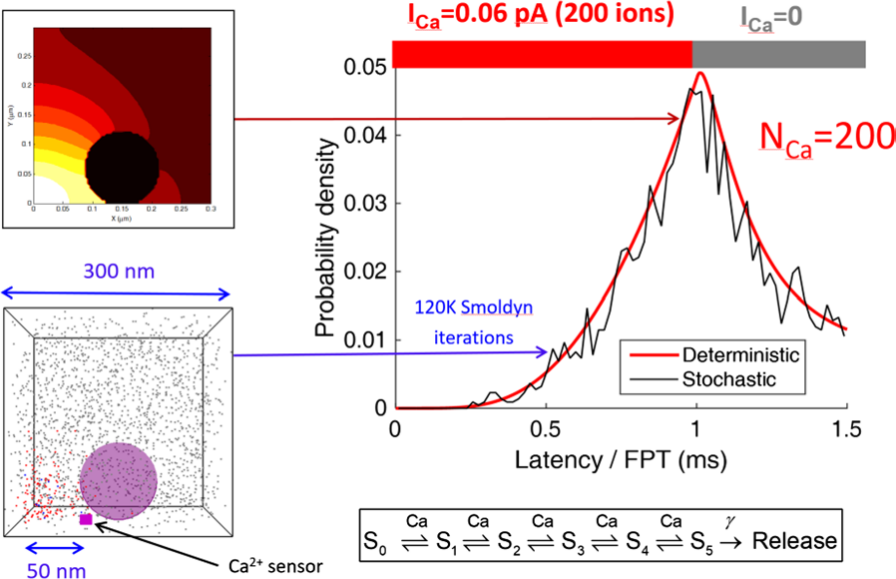
Close agreement between mass-action (red curve) and stochastic (black curve) calculations of the probability distribution of first passage time to the final release state of the exocytosis sensor (lower inset). A one-millisecond long square Ca2+ current pulse of about 0.06 pA is simulated, resulting in the entry of 200 Ca2+ ions into a sealed box, partially absorbed by the Ca2+ buffer (gray dots, lower left) with affinity of 1 μM and concentration of 20 μM. Simulations conducted using Smoldyn (www.smoldyn.org) and CalC (www.calciumcalculator.org)

**Acknowledgments**

This work is supported by NSF grant DMS-1517085.

**References**

1. Hake J, Lines GT: Stochastic binding of Ca2+ ions in the dyadic cleft; continuous versus random walk description of diffusion. *Biophys J* 2008, 94(11):4184–4201.
2. Modchang C, Nadkarni S, Bartol TM, Triampo W, Sejnowski TJ, Levine H, Rappel WJ: A comparison of deterministic and stochastic simulations of neuronal vesicle release models. *Phys Biol* 2010, 7(2):026008.
3. Weinberg SH, Smith GD: Discrete-state stochastic models of calcium-regulated calcium influx and subspace dynamics are not well-approximated by ODEs that neglect concentration fluctuations. *Comput Math Methods Med* 2012, 2012:897371.
4. Flegg MB, Rudiger S, Erban R: Diffusive spatio-temporal noise in a first-passage time model for intracellular calcium release. *J Chem Phys* 2013, 138(15):154103.
5. Felmy F, Neher E, Schneggenburger R: Probing the intracellular calcium sensitivity of transmitter release during synaptic facilitation. *Neuron* 2003, 37(5):801–811.

## P262 Spike timing based learning in neuronal networks induces a diverse range of states

### Benjamin Cramer^1^, David Stöckel^1^, Johannes Schemmel^1^, Karlheinz Meier^1^, Viola Priesemann^2,3^

#### ^1^Kirchhoff Institute for Physics, Heidelberg University, Heidelberg, Germany; ^2^Max Planck Institute for Dynamics & Self-Organization, Göttingen, Germany; ^3^Bernstein Center for Computational Neuroscience, University of Göttingen, Göttingen, Germany

##### **Correspondence:** Benjamin Cramer (benjamin.cramer@kip.uni-heidelberg.de)

*BMC Neuroscience* 2018, **19(suppl 2):**P262

We study the dynamics of spiking neural networks subject to synaptic plasticity driven by causality, emulated on accelerated, analog neuromporphic hardware. By adjusting the coupling to the external input or the degree of recurrence respectively, different dynamical regimes could be observed. For highly recurrent networks, long-tailed avalanche distributions are visible. Further, computationally relevant features develop, quantified by information theory. The applicability of the network for reservoir computing is tested within an auditory setup. By adjusting the coupling to the external input, network features could be selected and adjusted for a desired task.

## P263 Influence of inhibitory circuits in the olfactory bulb on the frequency tuning of mitral cells

### Rebecca Miko, Christoph Metzner, Volker Steuber

#### University of Hertfordshire, Biocomputational Rearch Group, Hatfield, United Kingdom

##### **Correspondence:** Rebecca Miko (rm17adc@herts.ac.uk)

*BMC Neuroscience* 2018, **19(suppl 2):**P263

Naturalistic odour stimuli have a rich temporal structure. It has been hypothesised that this structure contains information about the olfactory scene, for example the distance to an odour source [1, 2]. Furthermore, it has been suggested that animals might exploit this structure and extract this information in order to find odour sources [3]. As some of this information may lie in the frequency content of the stimuli [2], we studied input frequency dependent responses of mitral cells (MCs) in the olfactory bulb (OB), the first processing stage in the mammalian olfactory system. Specifically, we investigated whether MCs show frequency tuning and, if they do, how different components of the glomerular layer circuitry shape and determine the tuning. We used a model of the OB (modified from [4]) containing periglomerular cells (PGCs) and MCs, thus focusing on the recurrent and feed-forward inhibition in the glomerular layer. Simple sinusoidal currents of varying strengths and frequencies were used as input to the model. We constructed frequency tuning curves, extracted the peak resonance frequencies and looked at how these changed for different parameter combinations. We also considered the strength of the tuning, measured as (max firing rate − mean firing rate)/mean firing rate. We found that the resonance frequency decreased as the excitation of PGCs (both from the input and from the MCs) increased, whereas the strength of the PGC inhibition onto MCs did not seem to have a strong effect. Furthermore, the resonance strength increased with the strength of the excitatory connection between MCs and PGCs when the PGCs received sufficient external input from olfactory stimuli. These results suggest that the MCs can indeed show frequency tuning and that this depends on the strength of the excitatory synaptic input to PGCs, which provide inhibitory input to the MC. However, the observed frequency tuning occurred in a narrow range (19.5– 33.0 Hz). Future work should investigate how the OB could use this frequency tuning to obtain information about the surrounding olfactory scene.

**References**

1. Celani, A, Villermaux E, Vergassola M. Odor landscapes in turbulent environments. *Physical Review X* 2014, 4(4), 41015.
2. Schmuker M, Bahr V, Huerta R. Exploiting plume structure to decode gas source distance using metal-oxide gas sensors. *Sensors and Actuators B: Chemical* 2016, 235, 636–646.
3. Jacob V, Monsempès C, Rospars JP, et al. Olfactory coding in the turbulent realm. *PLoS Computational Biology* 2017, 13(12), p. e1005870.
4. Li G, Cleland TA. A two-layer biophysical model of cholinergic neuromodulation in olfactory bulb. *Journal of Neuroscience* 2013, 33(7), 3037–3058.

## P264 The combined effect of homeostatic structural and inhibitory synaptic plasticity during the repair of balanced networks following deafferentation

### Ankur Sinha, Christoph Metzner, Rod Adams, Neil Davey, Michael Schmuker, Volker Steuber

#### University of Hertfordshire, Biocomputational Rearch Group, Hatfield, United Kingdom

##### **Correspondence:** Ankur Sinha (a.sinha2@herts.ac.uk)

*BMC Neuroscience* 2018, **19(suppl 2):**P264

Although a number of previous experimental and theoretical studies have investigated network reorganisation following deafferentation down to the level of synaptic elements [4], the mechanisms that are involved in this process are still not completely understood. We examined the dynamics of the repair mechanism by incorporating activity dependent homeostatic structural plasticity [1] into a spiking neural network model balanced by inhibitory synaptic plasticity [6]. Results from our simulations suggest that the process of reconfiguration of lateral connectivity following sensory deprivation is extremely sensitive to the balance of excitation and inhibition (E-I) in the network. We find that while fast homeostatic inhibitory synaptic plasticity is able to re-establish the E-I balance in neurons outside the lesion projection zone (LPZ), it prevents them from transferring excitatory activity to the deprived neurons in the LPZ. On the other hand, uncontrolled disinhibition by suppression of homeostatic inhibitory synaptic plasticity initially allows deprived neurons to regain activity but fails to stabilise the network back to a functional balanced state. These observations are in accordance with findings that indicate that inhibition plays a critical role in network rewiring [2] seemingly by stimulating structural plasticity mechanisms seen during development [5]. The sprouting of inhibitory axons outwards from the LPZ, opposite to excitatory axons has also been observed, possibly to re-inhibit neurons outside the LPZ [3]. Therefore, we hypothesise that the ratio of excitation and inhibition must follow a specific trajectory in the different regions of the network to enable successful repair as has been observed in various studies. The model of structural plasticity implements the dynamics of synaptic elements as dependent on intrinsic properties of individual neurons only [1]. The configuration of the network, by the formation and removal of synapses therefore depends solely on the numbers of various synaptic elements. Our current work extends this model by considering other factors that affect network rewiring, such as the activity dependent stability of synapses, and inhibition gradient guided axonal sprouting [4], to build a more faithful simulation of the underlying dynamics. This will enable us to study the effects of network reorganisation after deprivation on its computational functions, such as associative memory.

**References**

1. Butz M, van Ooyen A. A Simple Rule for Dendritic Spine and Axonal Bouton Formation Can Account for Cortical Reorganization after Focal Retinal Lesions. *PLoS Comput Biol* 2013, 9(10), e1003259.
2. Chen JL, et al. Structural basis for the role of inhibition in facilitating adult brain plasticity. *Nature Neuroscience* 2011, 14(5).
3. Marik SA, et al. Large-scale axonal reorganization of inhibitory neurons following retinal lesions. *Journal of Neuroscience* 2014, 34(5).
4. Sammons RP, Keck T. Adult plasticity and cortical reorganization after peripheral lesions. *Current Opinion in Neurobiology* 2015, 35.
5. Vetencourt JFM, et al. The antidepressant fluoxetine restores plasticity in the adult visual cortex. *Science* 2008, 320, 5874.
6. Vogels TP, et al. Inhibitory plasticity balances excitation and inhibition in sensory pathways and memory networks. *Science* 2011, 334, 6062.

## P265 The role of chandelier cells in auditory steady-state response deficits in schizophrenia

### Christoph Metzner^1^, Bartosz Zurowski^2^, Volker Steuber^1^

#### ^1^University of Hertfordshire, Biocomputational Rearch Group, Hatfield, United Kingdom; ^2^University of Lübeck, Center for Integrative Psychiatry, Lübeck, Germany

##### **Correspondence:** Christoph Metzner (c.metzner@herts.ac.uk)

*BMC Neuroscience* 2018, **19(suppl 2):**P265

Since synchronized neuronal activity might underlie efficient communication in the brain, alterations thereof, as found in EEG/MEG studies of schizophrenic patients, might contribute to the symptoms characterizing schizophrenia [1]. A robust finding is a deficit in the gamma band auditory steady-state response (ASSR) [2]. Fast-spiking PV+ interneurons seem to be a major contributor to gamma oscillations. However, this class of inhibitory interneurons can be divided into at least two subgroups: basket cells (BCs) and chandelier cells (ChCs) [1]. Interestingly, for both subtypes cellular/molecular alterations have been identified in schizophrenia [1]. However, the role these two subgroups play during the generation of gamma oscillations, and during abnormal oscillations in schizophrenia, remains unresolved. We use a simple model, consisting of three populations of theta neurons: (1) pyramidal cells (PCs), (2) BCs and (3) ChCs (based on [3, 4]). We assume that the prolonged GABAergic decay time at ChC synapses is a major contributor to gamma and beta band ASSR deficits in schizophrenia [3] and model this by increasing the decay time constant for ChCs. We then explore the model behaviour in response to oscillatory inputs in the beta and gamma range, for different ratios of BCs vs. ChCs (BCs are known to be more numerous than ChCs [1]), different strengths of inhibition of the ChCs onto PCs (ChCs might exert powerful inhibition because of their synapses directly targeting the axon hillock of PCs [1]) and reductions in the strength of inhibition of BCs (a possible result of genetic alterations in schizophrenia [2]). At realistic BCs/ChCs ratios, increased ChC inhibition, due to increased decay times is not sufficient to strongly reduce gamma power as it has been described for schizophrenia patients. Under the assumption that they exert much more powerful control over PC firing stronger reductions were observed. However, the model did not reproduce other deficits that have been described in schizophrenia, such as an increase in beta power for 20 and 40 Hz stimulation [3]. Simultaneously reducing BC inhibition did not change this overall behaviour. Interestingly, prolonged decay times at BC-PC synapses led to both a strong decrease of gamma power and an increase in beta power, matching experiments more closely. We conclude that changes in the dynamics at ChC-PC synapses might not be a major contributor to gamma and beta band ASSR deficits in schizophrenia. Our results suggest that the more numerous BCs are likely to dominate the influence inhibitory interneurons exert on the PC population during oscillatory entrainment.

**References**

1. Gonzalez-Burgos G, Lewis D.A. NMDA receptor hypofunction, parvalbumin-positive neurons, and cortical gamma oscillations in schizophrenia. *Schizophrenia bulletin* 2012, 38(5), pp. 950–957.
2. Thune H, Recasens M, Uhlhaas PJ. The 40-Hz auditory steady-state response in patients with schizophrenia: a meta-analysis. *JAMA psychiatry* 2016, 73(11), pp. 1145–1153.
3. Vierling-Claassen D, Siekmeier P, Stufflebeam S, Kopell N. Modeling GABA alterations in schizophrenia: a link between impaired inhibition and altered gamma and beta range auditory entrainment. *Journal of Neurophysiology* 2008, 99(5), pp. 2656–2671.
4. Metzner C. Modeling GABA alterations in schizophrenia: a link between impaired inhibition and altered gamma and beta range auditory entrainment. *ReScience* 2017, 3(1).

## P266 Modeling rod-cone parallel processing in the retina

### Adree Songco Aguas^1^, Fred Rieke^1^, William Grimes^2^

#### ^1^University of Washington, Departments of Physiology & Biophysics, Seattle, WA, United States; ^2^National Institutes of Health, Neuroscience Department, Bethesda, MD, United States

##### **Correspondence:** Adree Songco Aguas (adree@uw.edu)

*BMC Neuroscience* 2018, **19(suppl 2):**P266

Parallel processing underlies computation in many neural circuits. Several common circuit motifs control how parallel processing contributes to circuit function: (1) divergence of common inputs to parallel circuits; (2) distinct linear shaping of signals in different parallel circuits; and (3) location of key circuit nonlinearities relative to the convergence points of signals from different parallel circuits. Interactions between rod and cone mediated signals in the retina provide an excellent opportunity to investigate these computational elements. Vision relies on inputs from both rod and cone photoreceptors across light conditions ranging from moonlight to dawn, and visual perception is strongly influenced by interactions between the resulting signals. To understand how retinal mechanisms contribute to these perceptual interactions, we aim to develop a model that predicts retinal output in response to temporally and spatially modulated images in dim and intermediate light. We will use direct retinal recordings from cells across the primate retina to constrain the architecture of our model and test its ability to capture key features of rod-cone interactions. The model will then be used to predict neural responses to novel stimuli, specifically focusing on identifying stimuli that highlight the importance of specific circuit features in shaping retinal outputs; significant discrepancies between predictions and empirical measurements will be utilized in finetuning the model. Ultimately, this model will improve both our understanding of how perceptually-relevant computation operates in parallel circuits and our ability to incorporate relevant computational features into devices (e.g. retinal prosthetics) that aim to replicate retinal function.

## P267 Simulation of avalanches in mouse primary motor cortex (M1)

### Donald Doherty^1^, Subhashini Sivagnanam^2^, Salvador Dura-Bernal^3^, William W Lytton^3^

#### ^1^SUNY Downstate Medical Center, Department of Anesthiology, Pittsburgh, PA, United States; ^2^University of California, San Diego, San Diego Supercomputer Center, La Jolla, CA, United States; ^3^SUNY Downstate Medical Center, Department of Physiology and Pharmacology, Brooklyn, NY, United States

##### **Correspondence:** Donald Doherty (donald.doherty@actionpotential.com)

*BMC Neuroscience* 2018, **19(suppl 2):**P267

Avalanches have been suggested to reflect a scale-free organization of cortex. It is hypothesized that such an organization may relate to a particularly effective form of activity propagation which is balanced between failure (activity fails to reach a target area) and overactivation (activity reaches a target area via many routes leading to wasted activity or epileptiform activity patterns). We electrically stimulated a computer model of mouse primary motor cortex (M1) and analyzed signal flow over space and time. Initially we stimulated a 300 μm × 600 μm slice of M1 using a 10 μm × 10 μm 0.5 nA stimulus across all 6 layers of cortex (1350 μm) for 100 ms. Waves of activity swept across the cortex for a half a second after the end of the electrical stimulus. We extracted avalanches from the data by counting events, spikes, occurring within 1 ms frames. An avalanche of length N was defined as N consecutively active frames, preceded by a blank frame and followed by a blank frame. A graph of the cortical slice above, with the 0.5 nA stimulus, displayed a bimodal distribution. We observed 18 avalanches in total with 4 single neuron avalanches and all the other avalanches containing more than 1000 neurons each. The largest avalanche contained 7000 neurons. Studies have generally shown avalanche activity to show a linear log–log graph starting highest from small avalanches and decreasing as the avalanches get larger. We looked at responses of M1 to lower amplitude stimuli between 0.05 and 0.5 nA to see if they may fit a classic inverse power-law curve. We graphed M1 response to a 500 ms electric stimulus at various amplitudes and found particularly clear inverse power-law responses to stimuli between 0.16 and 0.18 nA. In the 300 μm × 300 μm slice of M1 for 500 ms using 0.16nA we observed 90 avalanches from as small as a single neuron action potential in isolation to 13 neurons spiking. A large proportion were SOM neurons participating in the avalanches but they also included IT neurons at this level of stimulation. Neurons from every layer of cortex participated in avalanches except for layer 4. At stimulus onset neurons within an avalanche spiked at the same time. Spike onset amongst neurons within an avalanche became more heterogeneous as time progressed, especially after about 400 ms. For example, a 5 neuron avalanche began 431 ms after stimulus onset with a SOM6 neuron spike (x:84.2 μm, z:98.9 μm). Eight-tenths of a millisecond later it was followed with an IT5A spike (x:92.8 μm, z:85.7 μm). Next, after 0.65 ms, a different SOM6 neuron spiked (x:79.1 μm, z:83.2 μm) and finally the avalanche ended with yet another SOM6 spike (x:81.0 μm, z:64.3 μm). We observed similar results using a 0.18nA stimulus that elicited 110 avalanches from single neuron avalanches to avalanches that included 12 neurons. The simulation of avalanches in cortex offers advantages for analysis that are not readily done experimentally in in vivo or in vitro. We have been able to record from every neuron in our M1 slice and follow activity from cell to cell. In the future we will analyze how avalanches take place within and between layers.

**Acknowledgments**

Supported by NIH U01EB017695.

## P268 NetPyNE: a high-level interface to NEURON to facilitate the development, parallel simulation and analysis of data-driven multiscale network models

### Salvador Dura-Bernal^1^, Padraig Gleeson^2^, Samuel Neymotin^1^, Benjamin A Suter^3^, Adrian Quintana^4^, Matteo Cantarelli^5^, Michael Hines^6^, Gordon Shepherd^7^, William W Lytton^1^

#### ^1^SUNY Downstate Medical Center, Department of Physiology and Pharmacology, Brooklyn, NY, United States; ^2^University College London, Dept. of Neuroscience, Physiology & Pharmacology, London, United Kingdom; ^3^Institute of Science and Technology (IST), Austria; ^4^EyeSeeTea Ltd, United Kingdom; ^5^Metacell LLC, CA, United States; ^6^Yale University, Department of Neuroscience, CT, United States; ^7^Northwestern University, Department of Physiology, IL, United States

##### **Correspondence:** Salvador Dura-Bernal (salvadordura@gmail.com)

*BMC Neuroscience* 2018, **19(suppl 2):**P268

Experimental data is accumulating at an unprecedented—and accelerating—rate. However, as the BRAIN Initiative 2025 report points out: (1) even excellent quality data will not yield solid conclusions unless it is adequately integrated and interpreted, and (2) turning experimental knowledge into understanding inevitably requires rigorous theory and modeling. Biophysically realistic modeling provides a tool to integrate, organize and bridge data at multiple scales and develop hypothesis about the biological mechanisms underlying physiological and pathological brain function. NetPyNE (www.netpyne.org) is a high-level Python interface to the widely used NEURON simulator [1]. It provides high-level declarative language designed to facilitate the definition of data-driven multiscale models, e.g., a concise set of connectivity rules vs. millions of explicit cell-to-cell connections. The user can then easily generate NEURON network instances from these specifications, run efficient parallel simulations (with predefined setup for supercomputers), and exploit the wide array of built-in analysis functions (e.g. connectivity matrix, voltage traces, raster plot, information transfer measures). A recent feature provides the ability to place extracellular LFP recording electrodes at arbitrary 3D locations and plot the LFP signal, power spectra or spectrogram. All this functionality is also accessible via a graphical user interface (GUI) based on the state-of-the-art Geppetto technology. The GUI provides an intuitive way to define the model, including an interactive Python console and full synchronization with the underlying Python-based model (Fig. 1). The user can visualize the 3D network, run simulations and choose from the available analysis plots. NetPyNE’s standardized format clearly separates model parameters from implementation and can be exported/imported to NeuroML, thus making it easier to understand, reproduce, reuse and share models. This has motivated the conversion of several published models to NetPyNE specifications, including the Potjans&Diesmann cortical network, the Traub thalamocortical network, the Cutsuridis CA1 microcircuit and the Tejada dentate gyrus network. The tool is also being used to develop a variety of new models exploring mouse M1 microcircuits [2], the claustrum network, cerebellum circuits, transcranial magnetic stimulation (TMS) in cortex, or the underlying biophysics of EEG recordings. We expect the NetPyNE tool to make data-driven biophysically-detailed network modeling accessible to a wider range of researchers and students, including those with limited programming experience, and encourage further collaboration between experimentalists and modelers.

**Fig. 1:**
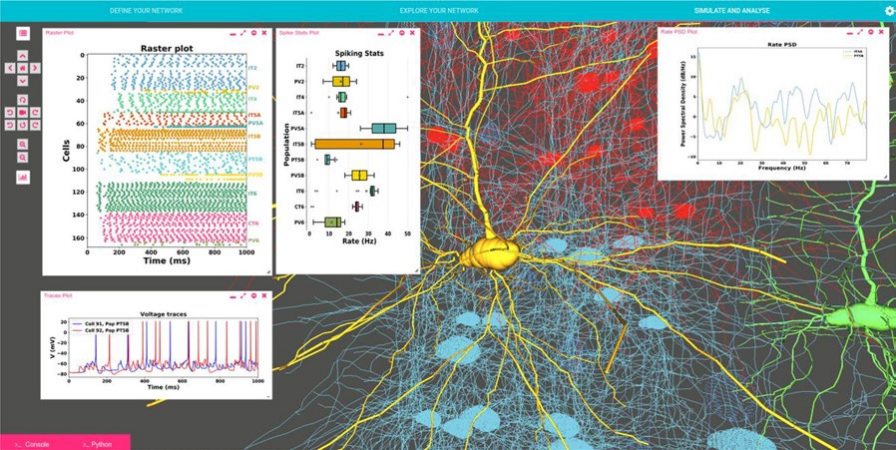
The NetPyNE GUI provides an intuitive way to generate, simulate and analyze data-driven biophysically detailed network models. NetPyNE GUI showing 3D representation of (simplified) M1 model and simulation results (raster plot, statistics, voltage traces and power spectra)

**Acknowledgments**

Research supported by NIH Grant U01EB017695, DOH01-C32250GG-3450000, NIH R01EB022903 and NIH R01MH086638.

**References**

1. Lytton WW, Seidenstein A, Dura-Bernal S, Schurmann F, McDougal RA, Hines ML. Simulation neurotechnologies for advancing brain research: Parallelizing large networks in NEURON*. Neural Comput*. 2016
2. Dura-Bernal S, Neymotin SA, Suter BA, Shepherd GMG, Lytton WW. Long-range inputs and H-current regulate different modes of operation in a multiscale model of mouse M1 microcircuits. *bioRxiv.* 2017 07 [Preprint]; 10.1101/201707

## P269 Extracellular reaction–diffusion in the NEURON simulator: modeling ischemic stroke

### Adam J. H. Newton^1^, Alexandra H. Seidenstein^2^, Robert A. McDougal^1^, Michael Hines^1^, William W Lytton^2^

#### ^1^Yale University, Department of Neuroscience, New Haven, CT, United States; ^2^SUNY Downstate Medical Center, Department of Physiology and Pharmacology, Brooklyn, NY, United States

##### **Correspondence:** Adam J. H. Newton (adam.newton@downstate.edu)

*BMC Neuroscience* 2018, **19(suppl 2):**P269

The NEURON simulation platform, featured in over 1900 publications, traditionally focused on models of neurons and networks of neurons. NEURON’s reaction–diffusion module (*rxd*) expanded support for 1D and 3D intracellular reaction–diffusion models [1]. This has been used to probe intracellular calcium dynamics in both physiological and pathological conditions. Originally *rxd* provided only limited extracellular support with an isolated space around each segment. We have extended*rxd*to include coarse-grained macroscopic models of the extracellular space [2]. NEURON thus allows detailed neuron models to be embedded in a 3D macroscopic model of tissue. Extracellular diffusion is implemented using the Douglas-Gunn alternating direction implicit method, an efficient scheme which supports parallelization. Reactions are now implemented using Just-In-Time compilation, allowing numerical integration to use faster compiled code rather than slow interpreted code. The macroscopic tissue model is based on a volume averaging approach, allowing the user to specify both the free volume fraction (the proportion of space in which species are able to diffuse) and the tortuosity (the average multiplicative increase in path length due to obstacles). These tissue characteristics can be spatially dependent enabling the modeler to account for differences in brain region or pathological effects of injury. We applied the *rxd* simulation framework to develop a model of ischemic stroke, which required multiscale coupling of electrophysiology with intracellular molecular alterations, and consideration of network properties in the context of bulk tissue alterations mediated by extracellular diffusion [3]. We initially modeled spreading depression triggered by elevated potassium in a cube of tissue (Fig. 1). Occlusion of a blood vessel in the brain triggers a cascade of changes, including: (1) synaptic glutamate release, related to excitotoxicity; (2) elevated extracellular potassium, leading to spreading depression; (3) cell swelling, reducing the extracellular volume and increasing the tortuosity; (4) production of reactive oxygen species, which give rise to inflammation. These cascades occur over multiple time-scales, with the initial rapid changes in cell metabolism and ionic concentrations triggering several damaging agents that may ultimately lead to cell death.

**Fig. 1:**
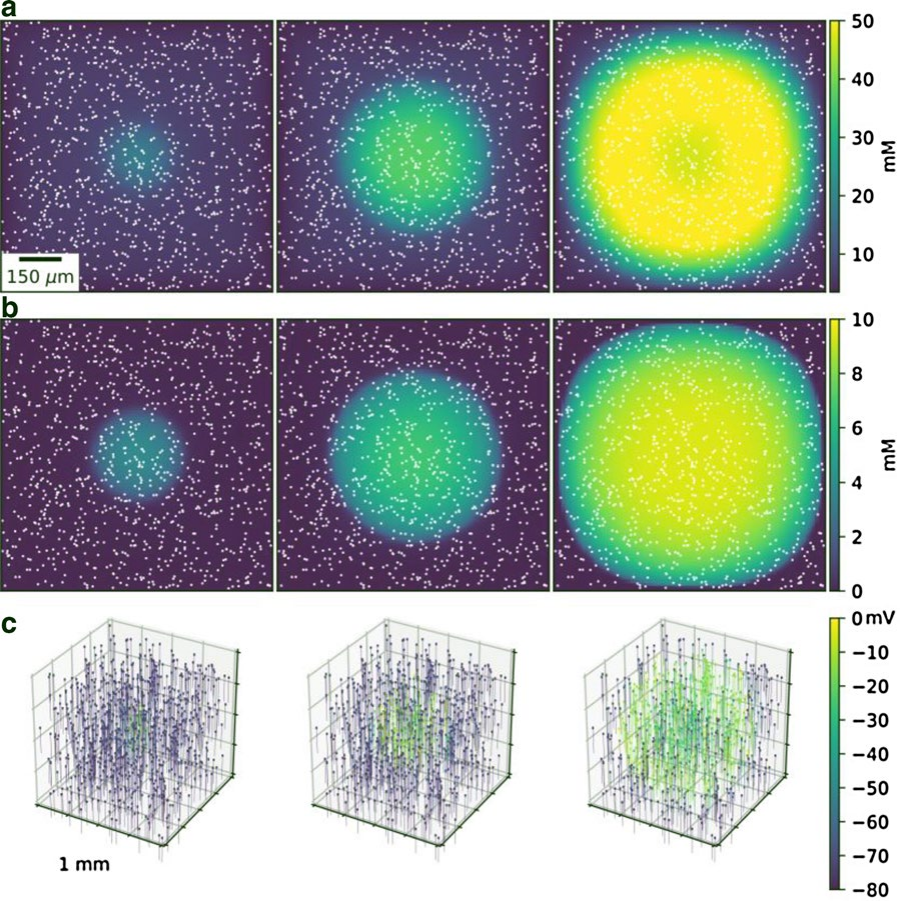
Spreading depression in a 1 cubic mm of tissue at 10, 20 and 30 s with 50,000 two compartment (soma, dendrite) neurons. (a) Extracellular potassium (b) Buffered concentration (based on a simple model of astrocyte buffering as an extracellular reaction) (c) The membrane potential of 1000 of the 50,000 neurons (positions shown in white in a & b)

**Acknowledgments**

Research supported by NIH Grant R01MH086638.

**References**

1. McDougal RA, Hines ML, Lytton WW. Reaction–diffusion in the NEURON simulator. *Frontiers in Neuroinformatics* 2013 7(28).
2. Newton AJH, McDougal RA, Hines ML, Lytton WW. Using NEURON to promote reproducibility in reaction–diffusion modeling of extracellular dynamics. *Frontiers in Neuroinformatics*. (in press).
3. Newton, AJH, and Lytton, WW. Computer modeling of ischemic stroke. *Drug Discovery Today: Disease Models* 2017.

## P270 Building and visualizing reaction–diffusion simulations in NEURON

### Robert A. McDougal^1^, Adam J. H. Newton^1^, William W Lytton^2^

#### ^1^Yale University, Department of Neuroscience, New Haven, CT, United States; ^2^SUNY Downstate Medical Center, Department of Physiology and Pharmacology, Brooklyn, NY, NY, United States

##### **Correspondence:** Robert A. McDougal (robert.mcdougal@yale.edu)

*BMC Neuroscience* 2018, **19(suppl 2):**P270

The NEURON simulator (neuron.yale.edu) provides a computational framework for studying not only networks of neurons but also the interplay between electrophysiology and chemical dynamics, (both intracellular and extracellular reaction–diffusion models). The models underlying these studies can be specified, simulated, and analyzed using both Python and graphical tools. NEURON’s graphical tools previously focused on supporting pure electrophysiology models. We describe a new integrated graphical toolset, powered by wxPython 4.x, for specifying and visualizing NEURON models incorporating both reaction–diffusion dynamics and traditional electrophysiology simulation. In comparison to electrophysiology models, these models feature new types of regions (1D and 3D, intracellular organelles, extracellular space, etc.), new types of kinetics, etc. Our toolset includes an expanded RxDBuilder supporting recent enhancements to NEURON’s reaction–diffusion capabilities, including extracellular and 3D intracellular simulations. The intracellular 3D graphical tools provide a detailed view of the cells morphology, enabling the modeler to select a region of interest over which to plot relevant intracellular concentrations. With the extracellular space, the GUI allows the modeler to choose to view the concentration dynamics for: a single voxel in the extracellular space, an average around the cell of section of interest, or over the whole extracellular space. To allow model changes from both the console and the GUI, the graphical tools are run in a separate thread that periodically polls the internal state; a function is provided to allow arbitrary wxPython windows to be run in the same thread, allowing user customization. For performance reasons, state variables are recorded in C++ during simulations; visualization occurs via Python at a user-specifiable interval. A session consisting of; the models, their current state and the graphical tools may be saved and loaded for future reuse. We demonstrate the utility of these model construction and visualization tools with a 3D intracellular calcium wave model and an extracellular model of spreading depression.

**Acknowledgments**

Research supported by NIH MH 086638.

## P271 Predicting runway excitation in nonlinear Hawkes processes

### Dmitrii Todorov, Wilson Truccolo

#### Brown University, Department of Neuroscience, Providence, RI, United States

##### **Correspondence:** Dmitrii Todorov (todorovdi@gmail.com)

*BMC Neuroscience* 2018, **19(suppl 2):**P271

In recent years, nonlinear Hawkes processes implemented as point process Generalized Linear Models (GLMs) have been proven to be a useful tool for analyzing microelectode array recordings. On a more theoretical side they are related to well known models of neuronal dynamics such as the spike response model and can capture the spiking temporal patterns of Izhikevich canonical models. Unlike ODE-based neuron models, point process GLMs can be fitted directly to the spike times data. For most relevant models, the models can be easily fitted using standard optimization tools, as the likelihood function is strictly convex. Despite the acknowledged utility of nonlinear Hawkes process GLMs, the dynamics of fitted models has attracted attention only recently. In particular, simulation of fitted models can often produce unphysiologically high firing rates, despite passing many goodness-of-fit tests. Here, “unphysiologically high rate” means “rate, close to 1/absolute refractory period,” reflecting “runaway excitation”. To make nonlinear Hawkes process GLMs useful for long-term prediction of neuronal activity and simulation studies, it is important to understand which model features can lead to runway excitation. The mathematical theory of nonlinear Hawkes processes is not fully developed. Prior studies (e.g. Bremaud and Massoulie, Ann Prob, 1996) have focused either on the mere existence of finite stationary firing rates (and do not allow to estimate their actual values in general), or on the theoretical examination of infinite neuronal networks with some degree of homogeneity in the connectivity, whereas actual recordings typically contain not more than several hundreds of neurons and lack homogeneity. The question we consider here is how to predict runway excitation for an arbitrary finite network of Hawkes processes. Several recent theoretical approaches, based on statistical physics, allow to approximate stationary firing rates for nonlinear Hawkes processes. Those include mean field approximations, 1-loop approximation (Ocker et al., PLoS CB, 2017), quasi-renewal (QR) approximation (Gerhard et al., PLoS CB, 2017) and the regular firing rate test. These approaches are quite different conceptually, were introduced in different settings and have limitations in different directions. E.g. mean field approximation does not work for neurons with absolute refractory period without additional adjustments; 1-loop approximation inherits the same issue but also shows poor accuracy for strong nonlinear functions (at least for some networks), whereas QR approximation is primarily designed to work for exponential nonlinearity only. Moreover, The QR approximation can lead to prediction of multiple “fixed points” (stationary firing rates) that may relate to the actual dynamics in a nontrivial manner. To summarize, so far the strengths and limitations of these different approaches have not been compared systematically. Also their application to real data has been limited. We present a study that compares how the above approaches work for simple single and multiple-neuron nonlinear Hawkes process GLMs and compare their predictions with simulations. We identify model features that make some approaches work much better than others. We show that, in some cases, the different approaches can complement each other. Finally we demonstrate how the different approaches work when being applied to multivariate nonlinear Hawkes process GLMs fitted to actual spiking data.

## P272 Multiunit activity patterns in neocortex predict upcoming seizures in human focal epilepsy

### Timothée Proix^1^, Mehdi Aghagolzadeh^1^, Leigh R. Hochberg^2^, Sydney Cash^3^, Wilson Truccolo^4^

#### ^1^Brown University, Department of Neuroscience & Institute for Brain Science, Providence, RI, United States; ^2^Brown University, U.S. Department of Veterans Affairs and Institute for Brain Science, Providence, RI, United States; ^3^Massachusetts General Hospital, MA, United States; ^4^Brown University, Department of Neuroscience, Providence, RI, United States

##### **Correspondence:** Timothée Proix (timothee_proix@brown.edu)

*BMC Neuroscience* 2018, **19(suppl 2):**P272

Methods to reliably predict seizures in patients with epilepsy have been sought for several decades. Reliable seizure prediction would have a major impact in the quality of life of people with pharmacologically intractable seizures, allowing for new seizure prevention therapies based on warning and closed-loop electrical stimulation systems [1]. While most seizure prediction systems have relied upon EEG and/or ECoG, the predictive value of intracortical neural signals remain little explored [2]. Here, we demonstrate that seizures can be predicted early in advance from the neural activity of small neocortical patches distal from the identified seizure onset areas. We used multiunit activity and local field potentials recorded via microelectrode arrays (Blackrock Microsystems, Salt Lake City, Utah) plus machine learning algorithms to show that interictal and preictal activity in people with focal seizures can be discriminated. Intracortical signals were recorded in 5 patients undergoing neuromonitoring for resective surgery from a neocortical area distal to identified seizure onset areas [3]. Preictal periods were defined as the one-hour period leading to a seizure with a 5-minute interval between the preictal period and the seizure onset time. Interictal periods excluded the four hours preceding any seizures [2]. This setting attenuates potential errors and uncertainty in the determination of actual seizure onset times and the separation or interictal and preictal periods. Long short-term memory (LSTM) recurrent neural networks were used to assess the predictive power of the different features extracted from the recorded neural activity signals. Substantial predicted power, as assessed by the area under the receiver operating characteristic curves, was achieved with a 90% score for at least one type of feature in each patient. Importantly, we show that successful prediction can be achieved based exclusively on the multiunit activity of recorded neurons detected by thresholding high-pass filtering the electric potentials. This result indicates that neural activity in the recorded local neocortical patch exhibited preictal changes not only in subthreshold postsynaptic potentials that could be driven by the distal epileptogenic areas, but also changes in the local neuronal spiking activity in the recurrent neocortical networks. Our findings indicate that large-scale neuronal networks are engaged beyond the identified epileptogenic seizure onset areas towards the onset of a seizure, and open new perspectives for seizure prediction and control by emphasizing the contribution of multiscale neural signals in these networks.

**References**

1. Cook MJ, O’Brien TJ, Berkovic SF, et al. Prediction of seizure likelihood with a long-term, implanted seizure advisory system in patients with drug-resistant epilepsy: a first-in-man study. *The Lancet Neurology* 2013, 12, 563–571.
2. Brinkmann BH, Wagenaar J, Abbot D, et al. Crowdsourcing reproducible seizure forecasting in human and canine epilepsy. *Brain* 2016, 139, 1713–1722.
3. Truccolo W, Donoghue JA, Hochberg LR, et al. Single-neuron dynamics in human focal epilepsy. *Nature Neuroscience* 2011, 14, 635–641.

## P273 Axonal dynamics: Signal propagation and collision

### Rosangela Follmann^1^, Epaminondas Rosa^1^, Wolfgang Stein^2^

#### ^1^Illinois State University, School of Information Technology, Normal, IL, United States; ^2^Illinois State University, School of Biological Sciences, Normal, IL, United States

##### **Correspondence:** Rosangela Follmann (rfollma@ilstu.edu)

*BMC Neuroscience* 2018, **19(suppl 2):**P273

Long-range communication in the nervous system is carried out with the propagation of action potentials along the axons of nerve cells. While typically thought of as being unidirectional, it is not uncommon for axonal propagation of action potentials to happen in both directions. This is the case because action potentials can be initiated at multiple ‘ectopic’ positions along the axon. Axons are endowed with ionotropic and metabotropic receptors for transmitters and neuromodulators that can alter membrane excitability, and initiate ectopic action potentials [1]. Action potentials generated at distinct sites, and traveling toward each other, will collide. Recently, it has been suggested that some biological axons may show properties of crossing action potentials [2], and that Hodgkin-Huxley type models may be inadequate for representing some axons. However, this view has been challenged in a subsequent study using a reduced Hodgkin-Huxley model [3]. As neuronal information is encoded in the frequency of action potentials, the rate of action potential collision and annihilation may affect the way in which neuronal information is received, processed and transmitted. Additionally, action potential collision and annihilation can be of relevance in the treatment of spinal cord injury along with chronic pain of peripheral origin [4]. Here we present numerical simulations and experimental results aimed at helping to elucidate the subject of colliding action potentials [5]. We introduce an axonal multicompartmental model with the compartments represented by Hodgkin-Huxley equations reciprocally connected to each other by diffusive coupling. The numerical simulations are capable of mimicking low frequency ectopic spiking with orthodromic and antidromic action potential propagation. They predict that colliding action potentials traveling in opposite directions annihilate and do not cross. We further discuss this matter in the context of axonal excitability and supernormality in the wake of action potential generation for neurons of type I and type II. We also present results of experimental work performed on the earthworm ventral cord and on the crustacean stomatogastric nervous system. Both numerical simulations and experimental outputs clearly and unambiguously demonstrate that annihilation is inevitable.

References

1. D. Bucher&J.-M. Goaillard, Prog. Neurobiol. 94, 307 (2011).
2. A. Gonzalez-Perez et all. Phys. Rev. X, 4(3):031047, 2014.
3. S. R. Meier, PloS one, 10(3):e0122401, 2015.
4. X. Zhang, et all., IEEE Trans. Biomed. Eng. 53, 2445 (2006).
5. R. Follmann, E. Rosa Jr,&W. Stein. Phys Rev E 92 (3) 032707 (2015).

## P274 Optimal stimulation protocol in a bistable synaptic consolidation model

### Chiara Gastaldi, Samuel Muscinelli, Wulfram Gerstner

#### École Polytechnique Fédérale de Lausanne, Blue Brain Project, Lausanne, Switzerland

##### **Correspondence:** Chiara Gastaldi (chi.gastaldi@gmail.com)

*BMC Neuroscience* 2018, **19(suppl 2):**P274

Consolidation of synaptic changes in response to neural activity is thought to be fundamental for memory maintenance over a time scale of hours. In experiments, synaptic consolidation can be induced by repeatedly stimulating presynaptic neurons. However, the effectiveness of such protocols depends crucially on the repetition frequency of such stimulations and the mechanisms that cause this complex dependence are unknown.

Here we propose a simple mathematical model that allows us to systematically study the interaction between the stimulation protocol and synaptic consolidation. We show the existance of optimal stimulation protocols and we explain the results using phase-plane techniques. Our model explains why the temporal structure of LTP induction protocols, in particular the repetition frequency, is important for the outcome of plasticity experiments (Fig. 1).

**Fig. 1:**
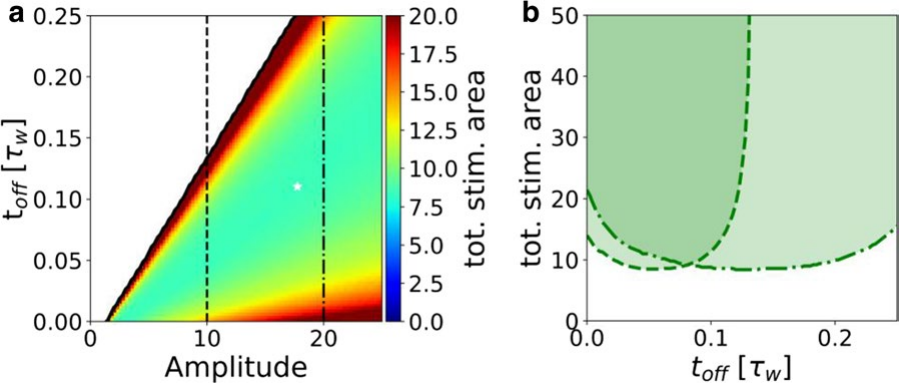
Stimulation time need to achieve potentation for large time-scale separation and pulse duration = 0.01. (a) In the potentiation domain is colored in proportion to the stimulation area (or “total power”) needed to achieve potentation with a repetitive pulse stimulus. The minimum stimulation area is 8.3, it is indicated by the white star and corresponds to the inter-pulse interval = 0.11τw and amplitude = 17.75. (b) The section of figure A for amplitude = 10 (dotted line) is enlarged. One can notice that for a fixed stimulation amplitude, there is an optimal frequency value that minimizes the stimulus area required to achieve potentation. Dotted and light-shaded: same for amplitude = 20

## P275 Oscillations and chaos in adaptive neural networks

### Samuel Muscinelli^1^, Tilo Schwalger^2^, Wulfram Gerstner^3^

#### ^1^École Polytechnique Fédérale de Lausanne, School of Life Sciences, Lausanne, Switzerland; ^2^École Polytechnique Fédérale de Lausanne, Laboratory of Computational Neuroscience., Lausanne, Switzerland; ^3^École Polytechnique Fédérale de Lausanne, Blue Brain Project, Lausanne, Switzerland

##### **Correspondence:** Samuel Muscinelli (samuel.muscinelli@epfl.ch)

*BMC Neuroscience* 2018, **19(suppl 2):**P275

Biological neurons exhibit an extended richness of biophysical mechanisms besides passive input integration. Among these, spike frequency adaptation has received great interest due to its richness in time scales [1] that allows sensory neurons to optimally transmit information [2]. Understanding the effect of such history-dependent processes on the dynamics of a recurrent neural networks has proven to be a hard task. Recent developments in mean-field approaches in the presence of such history-dependent processes [3, 4] allow to compute the mean and fluctuations of the network activity. To obtain the temporal structure of the recurrently generated fluctuations however, one has to solve the system self-consistently for the fluctuations. This can be done, in the large-N limit of non-adaptive, randomly connected networks of rate units, using Dynamical Mean-Field Theory (DMFT), thanks to which it was first shown the existence of a quiescent phase and a chaotic phase in the network dynamics [5]. However, this technique was until now restricted to non-adaptive networks. Here we apply DMFT to a randomly connected network of adaptive rate neurons. The technical challenge emerging from this setting is that the resulting mean-field system is two-dimensional, causing standard DMFT techniques to be not applicable. We propose an iterative method that allows fast computation of the mean power spectral density of the network activity. We show that in a large portion of the adaptation parameter space, the dynamics of the adaptive neural network is qualitatively different from the non-adaptive one. Besides the purely chaotic and purely quiescent phases, the adaptive network features two new phases. For strong recurrent connectivity and strong adaptation, the chaotic dynamics exhibit a finite-width peaked power spectral density, which means that in the DMFT limit the system can be described as a stochastic oscillation. For lower connection strength, a bistable phase also emerges, in which a stable fixed point coexists with limit cycles, even in the large-N limit. Finally, we extend the well-known result for the eigenvalue spectrum of Gaussian random matrices [6], to the adaptive case. This allows us to compute the stability of the zero fixed-point, and we show that it approximately predicts the separation between pure chaos and stochastic oscillations and the oscillation frequency at the criticality.

The additional dynamical richness of adaptive neural networks could explain the better performance in learning tasks that require integration of information over long time scales [7]. This could highlight a novel important role of adaptation that arise through network-level interactions.

**References**

1. Pozzorini C, Naud R, Mensi S, Gerstner W. Temporal whitening by power-law adaptation in neocortical neurons. *Nature neuroscience* 2013, 16(7), 942.
2. Fairhall AL, Lewen GD, Bialek W, van Steveninck RRDR. Efficiency and ambiguity in an adaptive neural code. *Nature* 2011, 412(6849), 787.
3. Deger M, Schwalger T, Naud R, Gerstner W. Fluctuations and information filtering in coupled populations of spiking neurons with adaptation. *Physical Review E* 2014, 90(6), 062704.
4. Schwalger T, Deger M, Gerstner W. Towards a theory of cortical columns: From spiking neurons to interacting neural populations of finite size. *PLoS computational biology* 2017, 13(4), e1005507.
5. Sompolinsky H, Crisanti A, Sommers HJ. Chaos in random neural networks. *Physical review letters* 1988, 61(3), 259.
6. Girko VL. Circular law. *Theory of Probability & Its Applications* 1985, 29(4), 694–706.
7. Muscinelli SP Gerstner W. Long timescale sequence recognition using adaptive neural networks. Conference on Cognitive Computational Neuroscience 2017.

## P276 Adaptation in a cascaded, image-computable model of cortical area MT

### Saba Entezari^1^, Pamela M Baker^2^, Wyeth Bair^2^

#### ^1^University of Washington, Mechanical Engineering, Seattle, WA, United States; ^2^University of Washington, Biological Structure, Seattle, WA, United States

##### **Correspondence:** Wyeth Bair (wyeth0@uw.edu)

*BMC Neuroscience* 2018, **19(suppl 2):**P276

**Introduction:** Adaptation is a ubiquitous property of cortical neurons, but how adaptation alters the encoding of sensory inputs across multiple stages of processing is not well understood. The pathway from V1 to MT is ideal for understanding how adaptation at one stage (V1) influences the encoding in downstream neurons because past work has characterized changes in selectivity in V1 and MT as a result of a diverse set of adaptation paradigms and circuit-level models for this pathway have been proposed. Nevertheless, there is currently no image-computable model of MT responses that includes adaptation. Thus, we developed such a model to better understand at the single-unit and circuit level how the encoding of visual motion and the emergence of pattern direction selectivity is altered by adaptation across cortical stages.

**Methods:** We added several mechanisms of adaptation to our image-computable model of MT component direction selective (CDS) and pattern direction selective (PDS) neurons (Baker&Bair, 2016, J Neurosci; Baker&Bair, ModVis 2017). The model includes spatial integration from V1 to MT, V1 iso-orientation surround suppression (IOSS) and normalization stages in V1 and MT. First, we implemented single-stage gain adaptation on the raw motion energy signals in twelve direction channels at each spatial location. The adapted signals are then used to compute the surround suppression signal and a spatially local classical untuned normalization signal. The normalized signals pass through a V1 opponency stage before being normalized and integrated (across space&direction) at the MT stage to form CDS and PDS units. Second, we implemented a recently proposed form of adaptation in which normalization weights between units are updated by a learning rule that aims to achieve pairwise response-product homeostasis (RPH; Westrick et al., 2016, J Neurosci), extending this mechanism from orientation to direction channels.

**Results:** For single-stage gain adaptation, we found that the effect on V1 tuning of prolonged adaptation to a single direction depended on the size of the adapting and test stimuli (drifting grating patches optimized for the unit under study) in a manner qualitatively consistent with electrophysiological results in terms of response amplitude (Patterson et al., 2013, J Neurosci). However, tuning curves showed attractive shifts in the presence of untuned normalization when flank adaptation was limited to the classical receptive field (CRF), unlike repulsive shifts reported in the literature. When untuned normalization was omitted, there were no attractive shifts and no shifts in tuning for MT CDS cells, but there were repulsive shifts for PDS cells, contrary to the literature. For RPH normalization, we were able to achieve the desired repulsive shifts in V1 direction tuning for flank adaptation, but direction channels not driven by the adapter showed implausibly large increases in gain.

**Conclusions:** Our results so far suggest that simple combinations of mechanisms believed to be fundamental to processing along the V1-to-MT pathway are not sufficient to account simultaneously for the influences of adaptation across a diverse set of stimulus configurations in the CRF and surround. To remedy this, we are implementing two-stage gain adaptation and exploring alterations to RPH normalization that can better account for physiological data.

**Acknowledgements**

We thank Adam Kohn for advice. Funding: NIH R01 EY027023-01.

**References**

1. Baker PM, Bair W. A Model of Binocular Motion Integration in MT Neurons. *Journal of Neuroscience* 2016, 36(24):6563–6582
2. Baker P.M., Bair W. Unifying Binocular, Spatial, and Spatiotemporal Frequency Integration in Models of MT Neurons. Computational and Mathematical Models in Vision (MODVIS) workshop 2017; St Pete Beach (FL).
3. Westrick ZM, Heeger DJ, Landy MS. Pattern Adaptation and Normalization Reweighting. *Journal of Neuroscience* 2016. 36 (38) 9805–9816.
4. Patterson CA, Wissig CW, Kohn A. Distinct effects of brief and prolonged adaptation on orientation tuning in primary visual cortex. *Journal of Neuroscience* 2013. 33 (2) 532–543.

## P277 Balancing of Orientation preference in primary visual cortex

### Ang Li^1^, Si Wu^1^, Ye Li^2^, Xiaohui Zhang^1^

#### ^1^Beijing Normal University, State Key Laboratory of Cognitive Neuroscience and Learning, Beijing, China; ^2^Zhejiang University, Interdisciplinary Institute of Neuroscience and Technology, Hangzhou, China

##### **Correspondence:** Ang Li (d0u.gie@hotmail.com)

*BMC Neuroscience* 2018, **19(suppl 2):**P277

How features encoded by neurons are reorganized during development is not well understood. We examined this question in the context of visual processing in primary visual cortex (V1) of mice. Recent experimental studies on mice suggest the distribution of preferred orientations and directions of neurons in primary visual cortex (V1) are balanced after eye-opening, through an activity-dependent mechanism [1, 2]. At eye-opening, the orientation preferences of V1 neurons are highly biased towards the horizon axis; as time goes on, neuronal preferences to other orientations emerge via visual experiences. This developmental balancing is considered to be important for optimal processing of the visual environment [3–5]. To achieve balancing of preferred orientations, two general conditions must be met. (1) The encoded feature should be plastic to achieve balancing during development while stable enough to ensure normal visual function. (2) There exists intrinsic signaling mechanisms that specifically guide neurons to develop under-represented orientations. We propose that inhibitory neurons in the network are capable to provide such mechanism. The first condition guarantees the onset of reorganization during visual development, while the second condition guarantees the reorganization process to be bias balancing. We proposed a computational model to unveil the underlying mechanism for the balancing of orientation preference in V1. We built a 2-layer network consists excitatory and inhibitory neurons. The first layer is composed of Poisson excitatory neuron, which provides tuned input from external visual stimuli. The second layer consists inhibitory neurons as well as two types of excitatory neurons: senior excitatory neurons, which have the initial biased preference to the horizon axis, freshman excitatory neurons, which have no preference before learning. All excitatory neurons receive feedforward currents conveying orientations of external inputs as well as recurrent connections between themselves. These connections are subject to spiking-time-dependent-plasticity (STDP), which shapes the tuning property of excitatory neurons. In the critical period, inhibitory neurons become mature, and through reciprocal connections, they mediate mutual inhibition between excitatory neurons, leading the freshman neurons to encode orientations unrepresented in the network. For instance, when the horizon direction is presented, because of the strong responses of senior neurons, inhibitory neurons become active, which suppress freshman neurons, so that freshman neurons are less likely respond to the horizon direction; on the other hand, when an underrepresented orientation is presented, inhibitory neurons are less active, and freshman neurons learn to encode this orientation via STDP. After onset of visual experience, the network undergoes a balancing process that equalizing the initial bias in distribution of preferred orientations. Our model successfully reproduced the balancing of orientation preferences in V1, and unveiled the important role of inhibitory neurons in the visual development (Fig. 1).

**Fig. 1:**
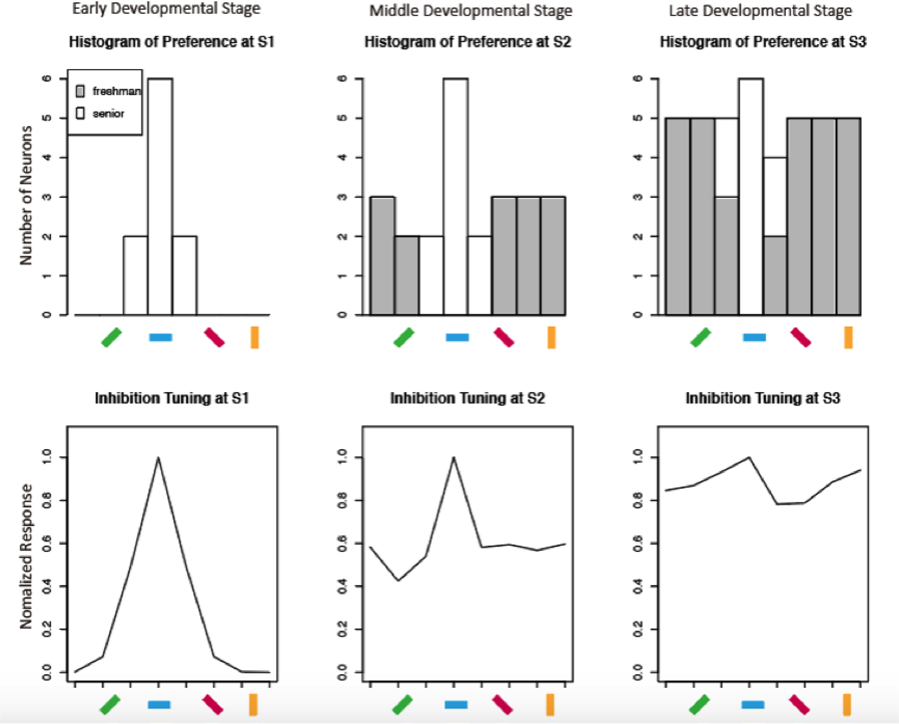
The emergence of balanced orientation selectivity in the network. The upper panel displays the distributions of neuronal orientation preferences at different developmental stages, which are: early stage (S1, just after eye-opening), middle stage (S2, during the development), and late stage (S3, mature). The lower panel displays the orientation tuning of the inhibitory neuron pool at different developmental stages. The inhibitory neuron pool is connected evenly to all excitatory neurons. At S1, its tuning property is determined by senior neurons. At S3, it no longer has preference, since the tunings of excitatory neurons cover the whole orientation space

## P278 Cholinergic modulation of reinforcement learning in the striatum

### Robert Capps^1^, Taegyo Kim^2^, Khaldoun Hamade^2^, Sergey Markin^2^, Dmitrii Todorov^3^, William Barnett^1^, Elizaveta Latash^1^, Yaroslav Molkov^1^

#### ^1^Georgia State University, Department of Mathematics & Statistics, Atlanta, GA, United States; ^2^Drexel University College of Medicine, Philadelphia, PA, United States; ^3^Brown University, Department of Neuroscience, Providence, RI, United States

##### **Correspondence:** Yaroslav Molkov (ymolkov@gsu.edu)

*BMC Neuroscience* 2018, **19(suppl 2):**P278

The striatum is a structure of the basal ganglia that is critical for reinforcement learning. In the striatum, cholinergic tonically active neurons (TANs) are thought to gate the dopaminergic input to medium spiny neurons during their involvement in action selection and reinforcement. TANs exhibit a context-dependent pause in their activity, during which the dopamine (DA) concentration in the striatum varies to encode reward prediction error (RPE), i.e. the difference between the expected and obtained reward. Although this mechanism has been the subject of many experimental studies, the role of TANs in motor learning is not well understood.

However, it is known that TANs generate a short burst in response to a stimulus, which is followed by a pause in TAN activity for several hundred milliseconds. During the pause, dopaminergic neurons modulate DA release into the striatum to encode the RPE and thus induce learning. After this pause, TANs return to normal tonic firing and striatal dopamine concentration stabilizes at its baseline levels. The duration of the TAN pause depends on dopaminergic inputs to TANs through activation of D2 receptors. During baseline tonic firing, TANs—being cholinergic—control the output of dopaminergic neurons by releasing acetylcholine (ACh) that binds to the nicotinic receptors of the latter. When a reward is presented, TANs receive a short stimulus from the thalamus. This short increase in TAN activity is then followed by inhibition via a slow after-hyperpolarization (sAHP) current, which lasts several seconds, inducing a pause in the tonic firing of TANs. Another current, the hyperpolarization-activated cation current (h-current) allows quick recovery from sAHP. The h-current in these neurons is down-regulated by DA via D2 receptors. Therefore, the TAN pause is produced by the slow AHP current, and the length of the pause is modulated by the faster h-current. Thus, encoding the RPE depends on the dynamic interactions between DA and ACh release mechanisms in the striatum. In this study, we constructed a mathematical model of ACh-DA interactions to clarify TANs’ role in reinforcement learning. We fit our model to the data obtained in electrophysiological experiments. Furthermore, we integrated the model ACh-DA interactions into our previously published model of the reward-based motor learning during center-out reaching movements. We simulated the effects of the striatal dopamine deficiency as observed in Parkinson’s disease patients. Additionally, we simulated and mechanistically explained the effects of administration of L-DOPA- a common treatment for early phases of Parkinson’s Disease- clarifying the mechanism by which L-DOPA recovers learning in these patients. In simulations, our model shows that both the baseline DA concentration and phasic DA release positively correlate with the duration of the TAN pause. Therefore, in the case of striatal DA deficiency, the loss of learning is associated not only with lower DA concentration but also with a shorter TAN pause, which means there is a shorter time period for learning to occur. We simulated L-DOPA administration by increasing the baseline concentration of DA in the striatum, which did allow partial recovery of motor learning functionality even though the magnitude of phasic DA release was not affected. Our model explains this recovery by L-DOPA-mediated prolongation of the TAN pause, which increases learning efficiency.

## P279 Brainstem mechanisms of cardio-respiratory coupling

### Elizaveta Latash, Robert Capps, William Barnett, Yaroslav Molkov

#### Georgia State University, Department of Mathematics & Statistics, Atlanta, GA, United States

##### **Correspondence:** Yaroslav Molkov (ymolkov@gsu.edu)

*BMC Neuroscience* 2018, **19(suppl 2):**P279

The respiratory and cardiovascular systems work together to oxygenate tissues and remove carbon dioxide and are physiologically integrated. Central neural circuits that control the respiratory and cardiovascular functions are located in brainstem and receive sensory feedback to maintain the gas homeostasis. Respiratory and cardiovascular physiologic outputs are partially synchronized/modulated by each other, and the respective brainstem neuronal networks have reciprocal synaptic connections. However, no quantitative mechanistic description was suggested to explain specific aspects of the cardio-respiratory interactions and their alterations in certain pathophysiological conditions. Two major markers of cardio-respiratory interactions were previously identified: cardio-ventillatory coupling (CVC) and respiratory sinus arrhythmia (RSA). CVC is usually interpreted as a form of partial synchronization between cardiac and respiratory rhythm which is characterized by varying probability of a heartbeat to occur at different phases of the respiratory cycle. RSA is a phenomenon concerned with changes in heartrate at different respiratory phases, which is usually represented by the dependence of the inter-heartbeat interval (R–R interval) on the respiratory phase with R–R interval shortened during inspiration and prolonged during expiration. Due to similar representation, CVC and RSA are often confused. However, there is substantial experimental evidence that independent mechanisms mediate the two phenomena. Here, we introduce a closed loop model of the integrated respiratory and cardiovascular control system to describe mechanisms for both CVC and RSA. The model combines and extends our previous data-driven models that incorporated mechanisms of cardiovascular input to the respiratory system or respiratory input to the cardiovascular system. In this model, CVC is mediated by the pulsatile inputs from arterial baroreceptors to neurons of the respiratory central pattern generator (rCPG) with pulses corresponding to the increases and subsequent relaxations in arterial pressure caused by heart contractions. We implement baroreceptor input to the rCPG as excitatory projections from 2nd order baro-sensitive neurons of the nucleus of solitary tract (NTS) to the expiratory population of the rCPG. This makes the onset of inspiration less likely to occur right after the heartbeat thus reproducing a characteristic structure of the heartbeat probability distribution. Our model explains RSA by modulation of the vagal input to the sinoatrial node of the heart. By fitting the literature data, we suggest that RSA should be driven by respiratory modulation of vagal cardiac neurons from both inspiratory and expiratory rCPG populations to accurately reproduce the experimentally observed dependence of the average R–R interval duration on the respiratory cycle phase.

## P280 Cortical dynamics on multiple time-scales drive growth of smooth maps to- gether with local heterogeneity

### Caleb Holt^1^, Yashar Ahmadian^2^

#### ^1^University of Oregon, Department of Physics, Eugene, OR, United States; ^2^University of Oregon, Institute of Neuroscience, Eugene, OR, United States

##### **Correspondence:** Caleb Holt (cholt@uoregon.edu)

*BMC Neuroscience* 2018, **19(suppl 2):**P280

The primary visual cortex of higher mammals develops smooth maps for many features of the its neural receptive fields, such as preferred orientation or spatial frequency. Such maps may be beneficial in minimizing wiring lengths between neurons selective to similar feature. Nevertheless, even in visual cortices with smooth maps, the receptive fields of nearby neurons show considerable degrees of heterogeneity. Correspondingly, some receptive field features appear to be uncorrelated between nearby cells, and average signal correlations between nearby cells are near zero. Such a random, “salt-and-pepper” organization may in turn be advantageous in reducing the response redundancy of local cortical populations and increasing their information content. Thus a combination of smooth maps for some features, and salt-and-pepper organizations for others, may provide both the benefits of wiring length minimization and informational efficiency. Previous theoretical models have accounted for the development of cortical feature selectivity and feature maps based on activity dependent Hebbian plasticity. However, these models inevitably predict that the cortex either develops smooth maps for all features (when long-range recurrent cortical excitation is strong) or random distribution of preference for all features (if cortical recurrent excitation is weak); they fail to account for the above observed mixture. We propose that this failure stems in part from the fact that these models do not account for the intrinsic temporal dynamics of the cortex. If properly considered, cortical interactions at slow and fast time scales will couple to the slow and fast features of the stimuli, respectively. We show that, given appropriate forms for the cortical interaction and input correlations at different time scales, this coupling allows for the development of smooth maps for slow stimulus features and random preference distributions for fast features. In particular, by simulating and analyzing one- and two-dimensional topographic models of development of thalamocortical connectivity, we show that our framework can sustain both smooth maps and salt-and-pepper organizations, providing a more biologically plausible mechanism for receptive field feature development.

## P281 Large-scale cortical model based on structural connectivity on aging APOE-4 allele carriers

### Yasunori Yamada

#### IBM Research, Tokyo, Japan

##### **Correspondence:** Yasunori Yamada (ysnr@jp.ibm.com)

*BMC Neuroscience* 2018, **19(suppl 2):**P281

The apolipoprotein E epsilon 4 allele (APOE-4) is the strongest genetic risk factor for causing sporadic Alzheimer’s disease. Neuroimaging studies have revealed that the APOE-4 allele carriers are different in structural and functional network connectivity. Additionally, resting state functional magnetic resonance imaging studies have shown that these differences can be observed even in the absence of cognitive impairments or even before the onset of brain amyloid accumulation processes. However, how structural changes in the brain affect its activities and functions remains poorly understood. To help give us a better understanding, I built large-scale cortical models based on structural connectivity data from diffusion tensor imaging on aging APOE-4 non-carriers and carriers. Each cortical model consisted of 2.4 million spiking neurons and 4.8 billion synaptic connections. Using these, I simulated resting-state cortical activities, and investigated the distinctive properties observed in vivo at multi-scale levels. As a result, I found that intrinsic cortical activities of both models matched typical patterns and quantitative indices from biological observations. However, the cortical model based on the structural connectivity of the APOE-4 carriers significantly increased the complexity of neural ensembles, and reduced the structural–functional relationship of inter-areal connectivity as well as the functional connectivity. To gain insight into how these differences of intrinsic cortical activities influence cortical information processing, I also investigated the properties of the responses to cortical inputs. I found that the cortical model based on the data of the APOE-4 carriers decreased the degree of cortical responses as well as the number of cortical regions responding to the input compared with the model based on the data of the non-carriers. From these experiments, the results suggest that structural changes in APOE-4 carriers might bring about complex and unstructured intrinsic activities, which might result in reducing cortical information propagation. This computational approach allowing for detailed analyses that are difficult, or impossible in human studies, may help to provide a causal understanding of how structural changes influence cortical information processing.

## P282 Approximate Bayesian inference for a neural mass model of anaesthesia

### Philip Maybank^1^, Ingo Bojak^2^, Richard G. Everitt^1^, Ying Zheng^3^

#### ^1^University of Reading, Department of Mathematics & Statistics, Reading, United Kingdom; ^2^University of Reading, Schools of Psychology & Clinical Language Sciences, Reading, United Kingdom; ^3^University of Reading, Department of Biomedical Sciences & Biomedical Engineering, Reading, United Kingdom

##### **Correspondence:** Philip Maybank (p.maybank@pgr.reading.ac.uk)

*BMC Neuroscience* 2018, **19(suppl 2):**P282

We have developed a new method for approximate Bayesian inference for a class of mechanistic models, which we call “stable” differential equations [1]. These systems evolve with quasi-linear dynamics close to a stable fixed point, and we exploit this for posterior inference several orders of magnitude faster than other state-of-the art MCMC sampling methods. In particular, we use the Whittle likelihood with model spectra computed through eigenvalue decomposition of the Jacobian evaluated at the fixed point. Our method has a comparable computational cost to variational methods, such as those used in Spectral DCM [2], but makes fewer assumptions regarding the parametric form of the posterior distribution. This allows more accurate inference for models where the posterior distribution has strong dependencies and complicated nonlinear geometry, as is typical for biophysically realistic models. Induction with the general anaesthetic agent isoflurane, as described with a well-established neural mass model [3], is a suitably “stable” system for our inference method at least for some concentrations of this agent. Here we use the electroencephalogram (EEG) recorded from rats undergoing isoflurane anaesthesia at two distinct, monitored levels of isoflurane concentration, light (0.75 vol%) and deep (2 vol%), which appear to lead to quasi-linear time series in the recorded data. Whereas we do not use intermediate concentrations showing prominent burst-suppression patterns, cf. the theoretical models in [4]. EEG was recorded using a spider electrode (GVB-geliMED, Germany. Diameter: 6 mm) placed on top of the rat’s skull approximately above the barrel cortex [5]. The electrode was secured with a conductive EEG paste and the skull underneath the electrode was intact. Data were recorded during resting state at a sampling rate of 24,414 Hz. They were subsequently down-sampled by a factor of 100 before analysis. Using our novel methods and this unique data, we successfully infer the parameters of the neural mass model. This success is demonstrated through (i) inferred spectral densities that are consistent with nonparametric spectral estimates using Welch’s method—see the Fig. 1—and (ii) changes in inferred parameter values with anaesthesia level that are consistent with mechanistic effects of isoflurane [3]. These results open the way for future work wherein one may be able to estimate the (sub)cellular effects of psychoactive agents merely from recordings of electrocortical activity.

**Fig. 1:**
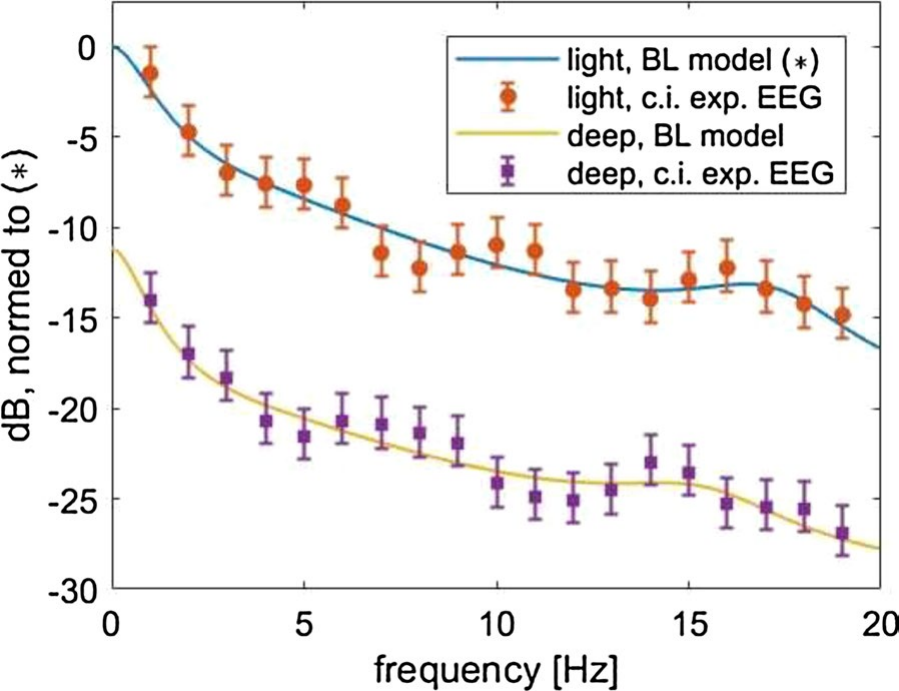
Spectral densities at light (blue line) and deep (yellow line) anaesthesia inferred approximately [1] with the Bojak-Liley (BL) model [3]. Nonparametric estimates using Welch’s method (orange and purple error bars: 95% confidence interval) are shown for comparison (20 s EEG, 1.4 s windows)

**References**

1. Maybank P, Bojak I, Everitt RG. Fast approximate Bayesian inference for stable differential equation models. *arXiv* 2017, arXiv:1706.00689 [stat.CO].
2. Moran RJ, Stephan KE, Seidenbecher T, Pape HC, Dolan RJ, Friston KJ. Dynamic causal models of steady-state responses. *NeuroImage* 2009, 44(3), 796–811.
3. Bojak I, Liley DTJ. Modeling the effects of anesthesia on the electroencephalogram. *Phys Rev* 2005, E 71:1–22.
4. Bojak I, Stoyanov ZV, Liley DTJ. Emergence of spatially heterogeneous burst suppression in a neural field model of electrocortical activity. *Front Syst Neurosci* 2015, 9, 18.
5. Kang S, Bruyns-Haylett M, Hayashi Y, Zheng Y. Concurrent Recording of Co-localized Electroencephalography and Local Field Potential in Rodent. *J Vis Exp* 2017, 129:e56447.

## P283 Modeling contrast gain control of fly photoreceptors

### Aurel A. Lazar, Nikul Ukani, Yiyin Zhou

#### Columbia University, Department of Electrical Engineering, New York, NY, United States

##### **Correspondence:** Nikul Ukani (nikul@ee.columbia.edu)

*BMC Neuroscience* 2018, **19(suppl 2):**P283

Early visual systems, both in vertebrates and invertebrates rapidly adapt to visual stimuli whose intensity and contrast vary orders of magnitude both in space and time [1], thereby achieving a performance that artificial cameras cannot. Although insights on the mechanism of contrast gain control has led to widely known models such as normalization [2], these models, in their current form, often 1) characterize the gain control only at steady-state or at peak transient levels. 2) lack a systematic framework for quantitative identification of the entire dynamical system. To model contrast gain control, we propose a functional framework comprising non-linear filters combined with divisive normalization, and provide algorithms for tractable identification of the filters in the model. We anchor our model around the photoreceptor-Amacrine-large monopolar cell (LMC) network in the fruit fly eye [3], whose outputs already exhibit adaptation. The overall divisive normalization (DN) framework used to model this network is shown in Fig. 1. Each input representing the light intensity received by each photoreceptor is first processed by a finite order Volterra Operator (VO), consisting of a linear and a quadratic filter. This process models the feedforward processing in the phototransduction process, and is denoted by VO-FF in the figure. This processed signal is then divisively normalized by a dynamic gain. The dynamic gain is determined by two factors. First, it is partly determined by the output of another VO processing the output of the photoreceptor itself. This models (temporal) modulation due to the feedback from an L2 neuron and is denoted by VO-FB in the figure. Second, it is partly controlled by the output of a finite order Multi-Input Volterra Operator (MVO) processing a pool of photoreceptor outputs. This models Amacrine cell mediated (spatial–temporal) modulation of the photoreceptor by neighboring photoreceptor outputs.

**Fig. 1:**
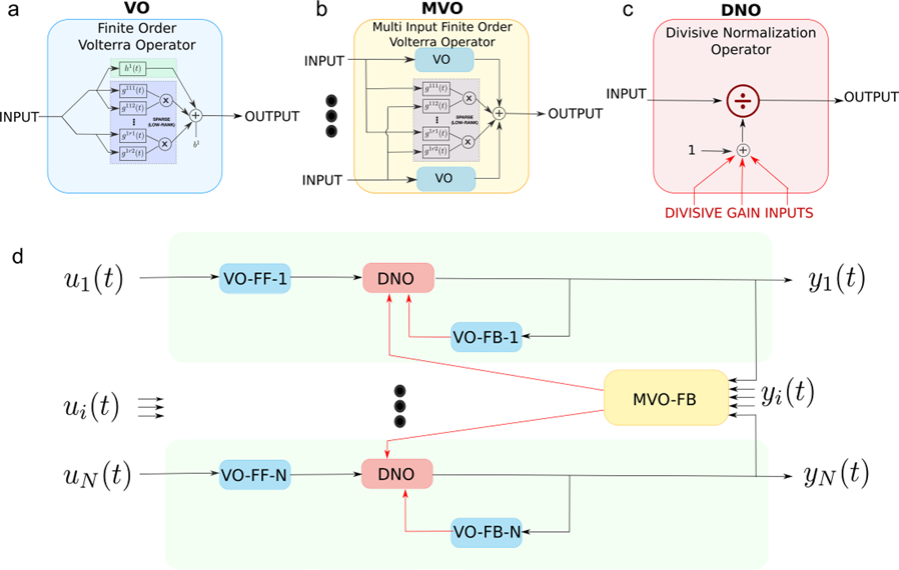
Schematic diagram of the Divisive Normalization Model

We demonstrate that such a model, with a very simple choice of filters, is capable of robust luminance and contrast adaptation and is able to respond reliably and efficiently to stimuli whose intensities vary over order of magnitudes. Further, we demonstrate that the tractability of identifying the filters in the DN model. Although we focused on the early stage of visual processing, every stage down the visual processing pathway, including motion detection, has been shown to be robust at various brightness and contrast levels. Adaptation to mean and variance of the stimuli has been observed in the early olfactory and auditory systems as well. The divisive normalization model that we describe here can be generally applied to the modeling and identification of these systems.

**References**

1. Rieke F, Rudd ME. The Challenges Natural Images Pose for Visual Adaptation. *Neuron* 2009, 64, 605–616.
2. Carandini M, Heeger DJ. Normalization as a canonical neural computation. *Nature Reviews Neuroscience* 2012, 13, 51–62.
3. Nikolaev A, Zheng L, et al. Network Adaptation Improves Temporal Representation of Naturalistic Stimuli in Drosophila Eye: II Mechanisms. *PLOS One* 2009, 4(1), 1–12.

## P284 Effect of floating point precision on dynamics of membrane potential in neural simulation

### Kazuhisa Fujita^1,2^, Yoshiki Kashimori^2^

#### ^1^Komatsu University, Dept. of Clinical Engineering, Komatsu, Japan; ^2^University of Electro-Communications, Dept. of Engineering Science, Chofu, Tokyo, Japan

##### **Correspondence:** Kazuhisa Fujita (k-z@nerve.pc.uec.ac.jp)

*BMC Neuroscience* 2018, **19(suppl 2):**P284

Many neuroscientists have used computer simulation. The accuracy of computer simulation is affected by not only a computational model and a numerical method but also floating point precision used in the simulation. The floating point types in C language are float (single precision), double (double precision), and long double (extended double precision). Generally, without consideration, we will select the double precision floating point for computer simulation. In recent years, a large-scale simulation and a real-time simulation of the neural system has been extensively attempted. For a large-scale simulation and a real-time simulation, not only a supercomputer but also a desktop workstation are used. In a simulation using a desktop workstation, a graphics board or an accelerator board with a GPU achieves acceleration of the simulation. When we perform a simulation using a graphics board, computational time with the single precision floating point is shorter than that with the double precision floating point. Furthermore, using single precision floating point can also reduce data transfer time. If the floating point precision has little effect on the accuracy of a simulation result, we can use single precision without worry and perform an efficiently accelerated simulation. In this study, we investigate the effect of the single precision, the double precision, and the extended double precision floating points on the dynamics of the neuronal activity in the computer simulation.

## P285 Decomposing adaptable elements of optokinetic response into cerebellar and non-cerebellar contributions by modeling and cerebellectomy approach

### Shuntaro Miki^1^, Robert Baker^2^, Yutaka Hirata^1^

#### ^1^Chubu University, Robotics Science and Technology, matsumoto-cho 1200 2422, kasugai-shi, Aichi, Japan; ^2^New York University, Department of Physiology & Neuroscience, 70 Washington Square South,New York, NY, 10012, NY, United States

##### **Correspondence:** Shuntaro Miki (mikisyun0@gmail.com)

*BMC Neuroscience* 2018, **19(suppl 2):**P285

**Introduction:** The optokinetic response (OKR) can be induced by large field visual motion in nearly all vertebrate species. It has been a popular model system to study cerebellum dependent motor control and learning. In response to visual velocity step stimuli OKR eye velocity exhibits two characteristic components: an initial rapid jump termed the Direct component, and a subsequent gradual rise termed the Indirect component [1]. Cohen et al. proposed an OKR model as shown in Fig. 1a which contains 3 parameters. Namely, a gain of the Direct pathway G1, a gain of the Indirect pathway G0, and a time constant of the velocity storage mechanism (VSM) in the Indirect pathway H. This simple model can reproduce OKR eye velocity well. Several studies have reported that the cerebellum is involved in the adaptation of OKR gain defined as eye velocity/visual stimulus velocity [2]. However, its contribution to generation and adaptation of the Direct and Indirect components has been controversial. Presently, we performed cerebellectomy before or after OKR gain adaptation in goldfish and evaluated the model parameters.

**Fig. 1:**
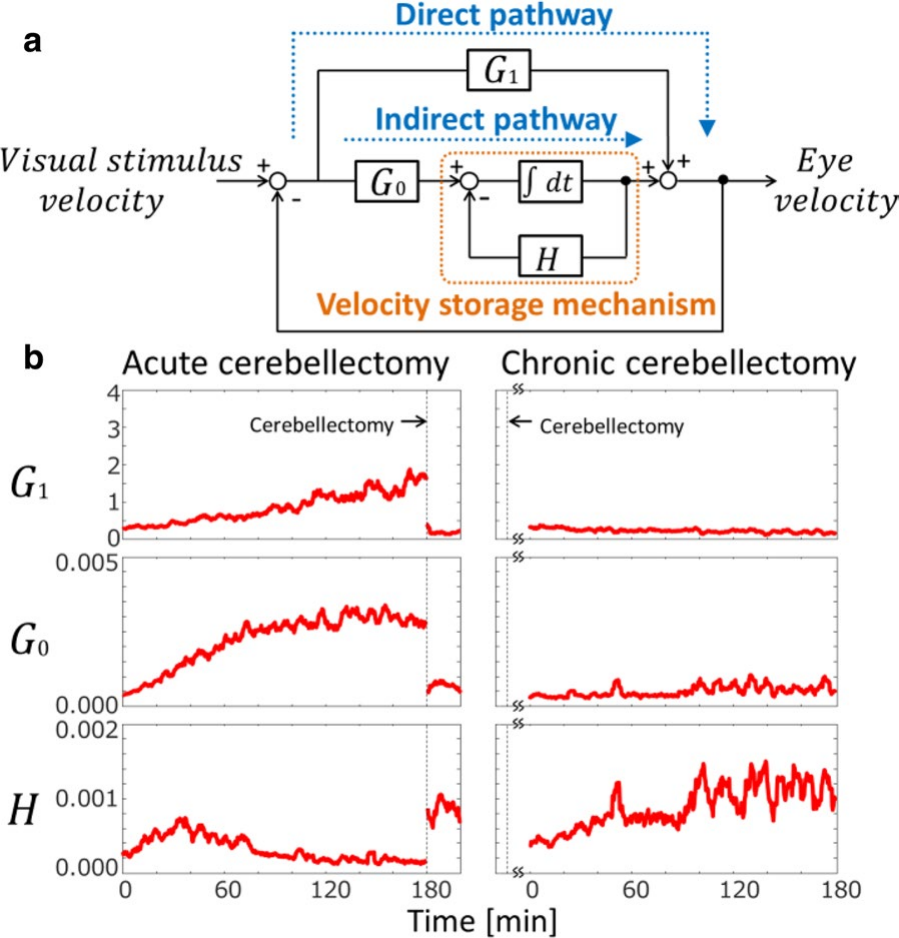
The OKR model by Cohen et. al, 1977 (a) and estimated model parameters (b) during OKR gain adaptation in acute (Left) and chronic cerebellectomy experiments (Right). In b, dotted lines indicate the timing of cerebellectomy

**Methods:** Goldfish were gently fixated at the center of a white cylindrical water tank with eye coils binocularly sutured on the cornea around the pupils for eye position measurement. Visual stimulus was projected on the wall of the water tank, and rotated in the clockwise direction at 20 deg/s for 8 s and stopped for 8 s repeatedly to generate horizontal OKR. This stimulation was continued for 3 h to induce OKR gain adaptation. Two kinds of experiments were conducted: Acute and chronic cerebellectomy. In the former experiment, normal goldfish (n = 8) underwent the 3-hour OKR training, then the cerebellum was acutely removed. In the latter experiment, cerebellectomy was conducted at least one week before the experiment, and the same 3-hour training was applied (n = 8). From eye velocity data recorded during these experiments, the parameters G1, G0and H of the OKR model implemented on MATLAB Simulink were estimated by fitting the unit step response of the model to the experimental data with a nonlinear optimization method (lsqnonlin function).

**Results:** In the acute cerebellectomy experiment (Fig. 1b, Left), G1and G0increased during the 3-hour training. H increased for the initial 20 min, but decreased thereafter and reached back to its pre-training value. After acute cerebellectomy, the increased G1and G0went back to their pre-training values. By contrast, the parameter H, which increased and then decreased during the training, increased after acute cerebellectomy. In the chronic cerebellectomy experiment (Fig. 1b, Right), G1and G0did not change during the 3-hour training while H increased gradually and did not show significant decrease unlike in the acute cerebellectomy experiment, and reached to a value comparable to that after acute cerebellectomy.

**Conclusion:** Changes in both Direct and Indirect components of OKR eye velocity represented by G1and G0in the model are totally cerebellum dependent. By contrast, change in VSM time constant represented by H consists of cerebellar and non-cerebellar contributions. These results suggest that OKR adaptation, specifically the changes in the VSM contains cerebellar and non-cerebellar adaptable elements.

**References**

1. Cohen J. *Statistical power analysis for the behavioral sciences* (Rev. ed.). Hillsdale, NJ, US: Lawrence Erlbaum Associates, Inc.
2. Kodama T, du Lac S. Adaptive Acceleration of Visually Evoked Smooth Eye Movements in Mice. *Journal of Neuroscience* 2016, 36(25), 6836–6849.

## P286 Differential functions of calcium dynamics in synaptic plasticity

### Yinyun Li, Zhong Zhang

#### Beijing Normal University, Department of Management, Beijing, China

##### **Correspondence:** Yinyun Li (leeyinyun@gmail.com)

*BMC Neuroscience* 2018, **19(suppl 2):**P286

Synaptic plasticity is intrinsically determined by calcium signalling in spines. In addition to the calcium influx into synapse through voltage gated calcium channels (VGCCs) and N-methyl-D-aspartate (NNMDA) receptors, the function of calcium released from internal store in mediating inter-synaptic cross-talk has barely been modeled. This work investigates how different sources of calcium contribute to inter-synaptic cross-talk and synaptic clustering. Based on experimental observations, we developed an abstract mathematical model in one-dimensional system with uniform distribution of spines with the connected dendrite. We modeled the biophysical process of calcium induced calcium release (CICR) in the dendritic smooth endoplasmic reticulum (SER). Our model compared distinct roles of calcium diffusion, back propagated action potentials (bAPs) and CICR played in synaptic clustering and inter-synaptic cross-talk. The simulation result demonstrated that calcium signal extruded from spine into dendrite requires amplification by CICR before invading neighboring spines to induce plasticity. Our model predicted that initial calcium concentration in SER may discriminate between different types of neuronal activity and induce completely different synaptic potentiation and depression.

## P287 Multilevel Monte Carlo for spiking neuronal networks

### Kevin Lin, Zhuocheng Xiao

#### University of Arizona, Department of Applied Mathematics, Tucson, AZ, United States

##### **Correspondence:** Kevin Lin (klin@math.arizona.edu)

*BMC Neuroscience* 2018, **19(suppl 2):**P287

A common task in computer modeling of large networks is to collect dynamical statistics like firing rates and correlations elicited by stimuli. This can be computationally expensive if the system at hand is sufficiently complex; the expense is amplified in tasks like parameter estimation and sensitivity analysis, which are necessary when dealing with data and intrinsically involve repeated model runs. This is especially the case for spiking network models, which typically involve interactions over a wide range of scales.

Multilevel Monte Carlo (MLMC) is a class of numerical methods invented to accelerate simulation-based statistical estimation. Originally developed for stochastic differential equation (SDE) models commonly used in, e.g., physics and finance, it has been extended to a variety of settings, including models of stochastic chemical kinetics. The basic idea behind MLMC is to make a fast but potentially biased estimate using large timesteps, then make a correction using smaller numbers of more expensive, small-timestep runs. MLMC is not universally applicable: its effectiveness depends on the underlying dynamics. But for certain types of systems, it can offer great speed-up. In this study, we assess the utility of MLMC for networks of spiking neurons, using a combination of mathematical analysis and numerical tests on prototypical models. Focusing on networks of leaky-integrate-and-fire (LIF) neurons, we have studied MLMC both by analyzing an associated Fokker–Planck equation and by numerical tests. Our main findings are 1) By studying a Fokker–Planck equation for coupling single cells, we found that MLMC is effective under broad conditions. By induction, MLMC is also effective for feed-forward networks. Since efficiency various continuously with parameters, MLMC can be effective for predominantly feed-forward networks. 2) Numerical studies of randomly-connected recurrent networks have shown that the effectiveness MLMC depends strongly on the parameter regime. In particular, for systems operating in a homogeneous, “mean-field”-like regime in which cells are only weakly correlated, we found MLMC to be rather efective. In contrast, for networks operating in partially-synchrnoous regimes, MLMC is less effective. Our results suggest that MLMC may offer significant speed-up for collecting statistics from spiking network models, particularly for predominantly feed-forward networks and for recurrent networks operating in a homogeneous regime. However, in situations where a recurrent network exhibits partial or full synchrony, straightforward extensions of MLMC may not be effective, and more work is required to develop efficient algorithms.

**Publisher’s Note**

Springer Nature remains neutral with regard to jurisdictional claims in published maps and institutional affiliations.

