# Correction to: Computing reward prediction errors and learning valence in the insect mushroom body

**DOI:** 10.1186/s12868-019-0486-8

**Published:** 2019-01-17

**Authors:** James Bennett, Thomas Nowotny

**Affiliations:** School of Engineering and Informatics, University of Sussex, Brighton, UK

## Correction to: BMC Neuroscience 2018, 19(suppl 2):P252 10.1186/s12868-018-0451-y

Following the publication of the original article [1], it was highlighted that an old version of the text for abstract P252 was published, thereby causing the text to no longer correspond with Fig. 1. The updated abstract text is included in this Correction article together with the figure.

**Fig. 1:**
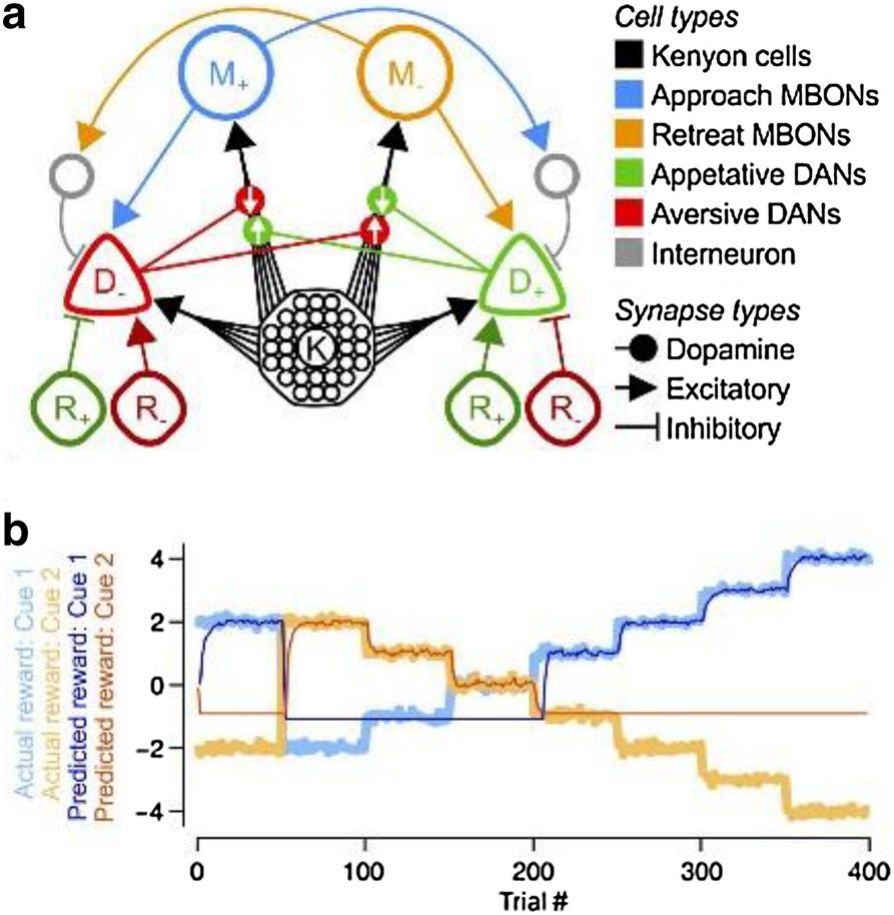
(**a**) Schematic of the Signed Reward Prediction Error Circuit. White arrows on the dopamine synapses indicate the relative change in synaptic weight with increases in dopamine released by the respective DAN. (**b**) Reward contingencies, and reward predictions computed from the MBON firing rates, in a two-alternative forced choice task. Reward predictions track the actual rewards associated with each option and become highly erroneous for options that are repeatedly not chosen

Decision making in the insect brain utilizes learned valence to bias particular actions in response to the animal’s environment. A key site for learning in insects is the mushroom body (MB) [1], where environmental cues are encoded by Kenyon cells (KCs) and assigned valence by MB output neurons (MBONs). Valence memories are learned via reward-modulated synaptic plasticity and stored in KC-MBON synapses, at which rewards are signaled by dopaminergic neurons (DANs). Recent studies in Drosophila have revealed intricate connections between these three cell types, which are necessary for learning appropriate actions [2,3]. Here, we present a MB model that captures these data to compute reward prediction errors (RPEs) for learning, thus implementing the Rescorla–Wagner model.

Current models posit that the alpha− (A) and beta− (B) lobes of the MB encode the signed valence of reward information and actions [1]: DANs in the A-lobe (hereafter called D−) are excited by negative (−ve) rewards, and depress active KC synapses onto MBONs that bias actions toward approach (M+); DANs in the B-lobe (D+) are excited by positive (+ve) rewards, depressing active KC synapses onto MBONs that bias actions toward retreat (M-). If MBONs provide excitatory feedback to their respective DANs, the learned reduction in MBON firing can offset the excitatory reward signal arriving at that DAN. Thus, D+ and D− may both encode RPEs in the signed (+ve or −ve) reward valence. Moreover, the difference in MBON firing rates, m_+_-m_−_, signals a reward prediction, i.e., the learned net valence associated with a sensory cue.

We first show two problems with this model: (1) It cannot learn reward magnitudes above an upper bound; (2) it learns only when KC-DAN excitation is minimal or absent, in contrast to experiments [2]. We propose a solution, in which D+/D− neurons are instead inhibited by −ve/+ve reward signals, and in which KC-DAN excitation is required. We also derive a plasticity rule for KC-MBON synapses that performs gradient descent on the RPE, and that resembles experimentally observed rules [4]. We call this model the Signed Valence Circuit (SVC). As before, DANs encode RPEs in the signed reward valence, and the difference in DAN firing rates, d_+_-d_−_, yields the net RPE.

In the SVC, D+/D−, respectively, signal RPEs for –ve/+ ve rewards, so do not actually contribute to learning +ve/-ve valences, counter to experimental evidence [1]. However, in a dual version of this circuit, in which D+/D− are driven by +ve/−ve rewards, D+ no longer signals decrements in –ve rewards, again in contrast with experiments [5]. We therefore combine the SVC and its dual to produce the Signed RPE Circuit (SRC; Fig. 1a), in which the lobes encode the signed RPE of both +ve and –ve reward signals. Both the SVC and SRC are able to learn rapid changes to reward contingencies (Fig. 1b).

Lastly, the SRC performs well in a traplining task, repeating learned routes and minimizing the distance traveled between feeding areas, a behavior exhibited by bees [6] and other species, and a foraging analog of the traveling salesman problem.

**References**

1. Owald & Waddell, Curr. Opin. Neurobiol. 2015, 35:178–184
2. Cervantes-Sandoval et al., eLife, 2017, 6:e23789
3. Felsenberg et al. Nature 2017, 544:240-244
4. Hige et al. Neuron 2015, 88:985–998
5. Perisse et al. Neuron 2013, 79:945–956
6. Lihoreau et al. Biol. Lett. 2012, 8:13–16

## References

[1] BMC Neurosci 2018, 19(Suppl 2):65. 10.1186/s12868-018-0451-y.

